# Age-related differences in network controllability are mitigated by redundancy in large-scale brain networks

**DOI:** 10.1038/s42003-024-06392-2

**Published:** 2024-06-07

**Authors:** William Stanford, Peter J. Mucha, Eran Dayan

**Affiliations:** 1 Biological and Biomedical Sciences Program, University of North Carolina at Chapel Hill, Chapel Hill, NC USA; 2 Department of Mathematics, Dartmouth College, Hanover, NH USA; 3 Department of Radiology and Biomedical Research Imaging Center, University of North Carolina at Chapel Hill, Chapel Hill, NC USA

**Keywords:** Cognitive ageing, Network models

## Abstract

The aging brain undergoes major changes in its topology. The mechanisms by which the brain mitigates age-associated changes in topology to maintain robust control of brain networks are unknown. Here we use diffusion MRI data from cognitively intact participants (n = 480, ages 40–90) to study age-associated differences in the average controllability of structural brain networks, topological features that could mitigate these differences, and the overall effect on cognitive function. We find age-associated declines in average controllability in control hubs and large-scale networks, particularly within the frontoparietal control and default mode networks. Further, we find that redundancy, a hypothesized mechanism of reserve, quantified via the assessment of multi-step paths within networks, mitigates the effects of topological differences on average network controllability. Lastly, we discover that average network controllability, redundancy, and grey matter volume, each uniquely contribute to predictive models of cognitive function. In sum, our results highlight the importance of redundancy for robust control of brain networks and in cognitive function in healthy-aging.

## Abstract (web summary)

A diffusion MRI study suggests that redundancy could mitigate the effects of age-associated changes in brain topology to support the average controllability of brain networks middle and old age.

## Introduction

As populations world-wide are aging^1^, dementia and other degenerative central nervous system diseases associated with cognitive decline are projected to increase in prevalence^2^. Cognitive decline is not restricted to pathological aging, but also occurs in healthy older adults. Yet healthy cognitive aging can vary greatly between individuals^3^. For those that resist cognitive decline, greater life-satisfaction, well-being, and higher levels of happiness are reported^4^. Several lifestyle factors have been found to contribute to successful cognitive aging, such as exercise^5^, and education^6,7^, yet the mechanisms that could support cognitive function late in life remain incompletely understood.

Studying the topological properties of macroscopic brain connectivity with tools from network science^8^ is one method by which the mechanisms that could promote cognitive function in aging were examined. Studies focused on measures of network topology that change throughout healthy^9–17^, and pathological aging^18–23^, and attempted to relate alterations in topology to cognition. One such central measure is network controllability^24^. Controllability is a concept that originated in engineering within the domain of control theory^25–27^. In networks, controllability examines the ability of key nodes to enable dynamic state transitions between an initial and target state^24^. The two most commonly studied forms of network controllability are average and modal controllability. Average controllability reflects a node’s ability to push the network into an easy to reach state^28^. In the brain the default mode network, a collection of brain regions more active at rest^29,30^, and believed to contain general priors for cognitive function^31^, has been observed to have several hubs of average controllability^28^. This positions the default mode network to easily direct the brain from a resting state towards activity relevant for behavioral tasks^30,32^. In contrast, modal controllability quantifies a node’s ability to push the network into difficult to reach states^33,34^, which has been shown to be important in brain networks associated with cognitive control^28,35^.

Network controllability has been postulated as a dual mechanism of brain and cognitive reserve in aging by combining structural connectivity, typically viewed as a mechanism of brain reserve, and brain dynamics believed to be necessary to support general cognitive processes to jointly measure the brain’s ability to respond and adapt to changing cognitive demands as a unified form of reserve^36^. In this framework, age-associated cognitive decline can be viewed as a breakdown of network control, in which individuals have difficulty transitioning to, or maintaining, specific brain states relevant for cognitive function. Controllability metrics enable researchers to estimate the ability of dynamic interactions between brain regions, mediated by the connections between them, to drive the brain towards brain states relevant for cognitive activity, and thus support the cognitive processes associated with cognitive reserve. Recent work has found that longitudinal changes in the modal controllability of cognitive control systems in aging could underlie age-associated declines in executive function^37,38^. Other work demonstrated that the ability of temporal-parietal regions to control other brain regions decreases with age, and is particularly vulnerable to simulated lesions^39^. However, it remains unknown how average controllability is influenced by aging, and whether these putative changes affect cognition. The properties of underlying network topology that could mitigate differences in average controllability in aging and thus facilitate reserve are also unknown.

A mechanism we hypothesize that the brain may use to mitigate age-associated alterations in average controllability is through increased redundancy^40–43^. Redundancy is a general principle ubiquitous in engineering that protects systems from the failure of individual components^44^. Redundancy is also evident in biological systems at many scales. Examples include at the level of genes^41,45^, organs^43^, and in population coding within neural networks^46^. In the context of brain networks, redundant paths are hypothesized to provide alternate routes for information transmission that could serve as a form of brain reserve^47–49^ supporting information transmission if some paths fail due to the effects of aging and/or disease. Redundant links have been previously identified as potential mechanisms that support robust control of complex networks during disconnections^24,50,51^, but this has not been investigated in the context of network control in aging brain networks. Furthermore, the compensatory effects of redundancy have been postulated as a neuroprotective mechanism^43^, but only recently studied within the context of healthy and pathological aging^12,19,23^. It was reported that functional hippocampal redundancy supports cognitive resilience in pathological aging^19,23^, and that network-wide functional redundancy mediates the relationships between age and executive function^12^. However, redundancy has yet to be investigated in the context of structural brain networks and the alterations in controllability they undergo in aging. We hypothesize that increased redundancy could mitigate the effect of age-associated topological degradations to enable robust average controllability of brain networks.

It was recently hypothesized that network controllability and brain volume, a more traditional measure of brain reserve, should each explain unique variance related to cognitive status^36^. In particular, because it is unlikely that reserve related to volumetric properties of the brain is entirely explained by the organizational properties of structural networks, like network controllability, they should have additive predictive value as proxies of cognitive function. In the current study we investigated this aforementioned hypothesis, as well as the relevance of redundancy in structural brain networks as a potential mechanism of brain reserve. We chose processing speed as the cognitive function evaluated because it is believed to be heavily dependent on communication along white-matter tracts^52,53^. Relatedly, processing speed is known to exhibit age-associated declines^54^, that correspond with changing topological properties of structural brain networks^55,56^. Processing speed is also associated with commonly used measures of brain reserve, such as hippocampal volume^57,58^. We hypothesized that processing speed would be related to measures of regional influence on network dynamics, such as average controllability, particularly in functional networks that have been reported as important in age-related differences in processing speed (e.g., default mode and frontoparietal control networks^15,59^). We expected that redundancy could support rapid communication between task-relevant functional networks^52,53,60^, and thus be positively associated with processing speed.

**Fig. 1:**
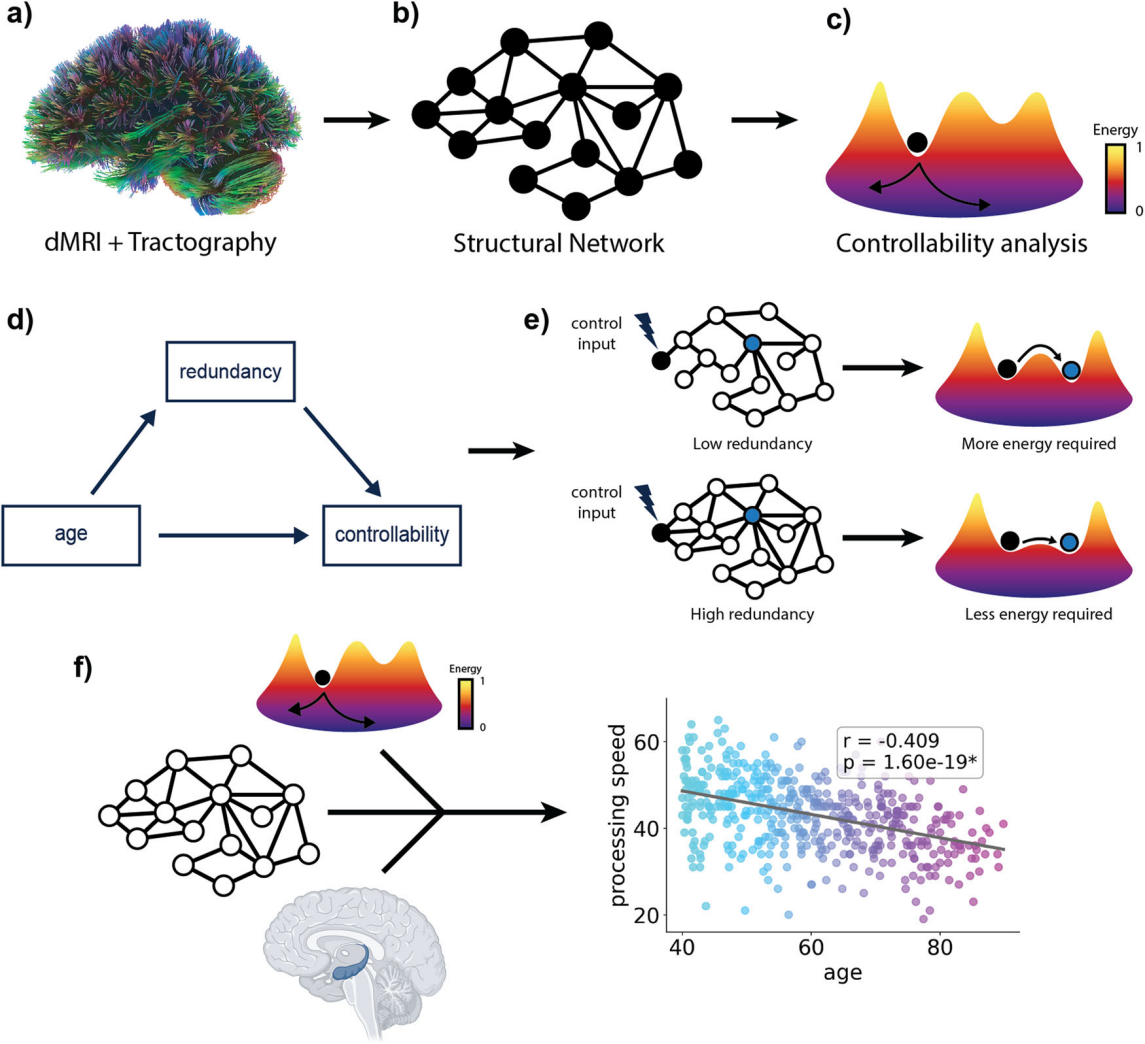
Study outline. **a** Diffusion MRI data from 480 subjects from the HCP Aging dataset were used in our study. **b** We constructed structural networks using the functional Schaefer local-global parcellation with 17 networks and 400 ROIs. **c** For each subject, we calculated network controllability, a measure of a node’s ability to steer the brain into easy to reach states. **d** We studied the relationship between controllability and network redundancy in aging, testing the extent to which redundancy influences the relationship between age and network controllability. **e** We hypothesized that redundancy would mitigate the effects of age-associated differences in topology on average controllability in brain networks. **f** Finally, we investigated the extent to which grey matter volume, network controllability, and redundancy, can jointly predict age-associated variance in cognitive function.

To test our hypotheses we used diffusion MRI (dMRI) data from 480 participants (female = 281, male = 199) between the ages of 40–90 from the HCP-aging dataset^61^ (Fig. 1a). We examined how average controllability, a region’s ability to push a network into easy to reach states, changes in aging. We constructed structural networks using the functional Schaefer local-global parcellation^62^ (Fig. 1b). For results presented in the main text, networks were thresholded by removing edges with streamline counts below 0.001 multiplied by the maximum streamline count per network. To investigate the robustness of our results, we repeated analyses with thresholds of 0.005, 0.010, and 0.015. After constructing structural networks, we computed average controllability for each brain region (Fig. 1c), and then identified age-related differences in average controllability of control hubs and large-scale networks (Fig. 1c). We hypothesized that the average controllability of control hubs and large-scale networks would be negatively related to participants’ age. Next, we investigated how redundancy, a measure of multi-step paths between nodes, supports average controllability in middle- and old-aged participants (Fig. 1d). Due to previous work that has highlighted the role of redundancy for control in complex systems^24,50,51,63^, we hypothesized that differences in network redundancy could mediate age-associated differences in in network controllability in brain networks (Fig. 1e). Finally, we investigated the extent to which grey matter volume, network controllability, and redundancy, could serve as partial proxies of age-associated variance in processing speed (Fig. 1f). We hypothesized that both redundancy and average controllability would serve as partial proxies of cognitive function that were complementary to the traditional measure of reserve in grey matter volume.

## Results

### Hubs of average controllability are largely consistent in middle- and old-aged adults

**Fig. 2:**
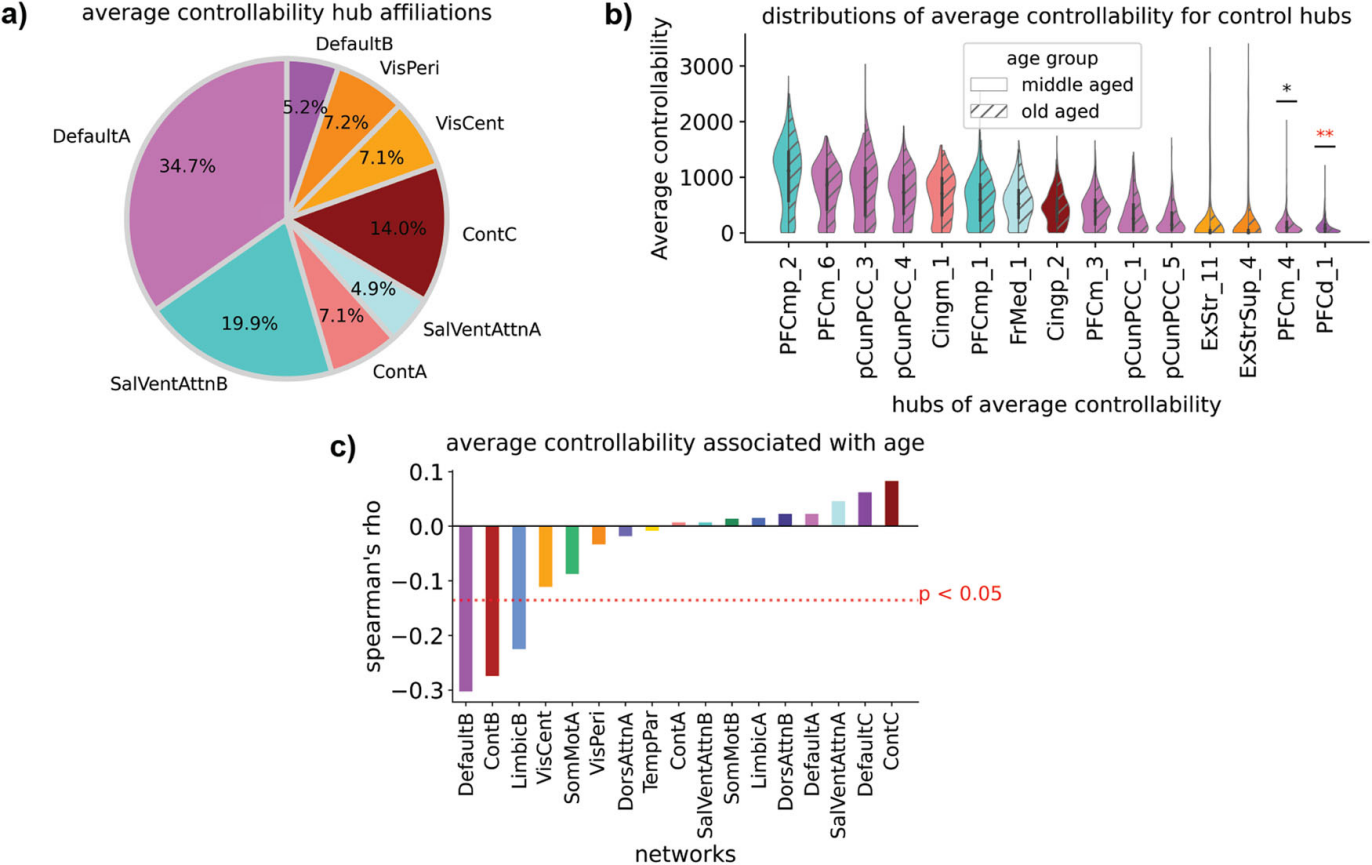
Hub and average network controllability are impacted by aging. **a** The affiliations of hubs of average controllability in middle-aged subjects (ages 40–65) were predominately within the default mode network. Percentages were corrected by network size, which equalizes the probability of hubs falling within each network. **b** Distributions of average controllability for each hub, for middle- and old-aged participants (ages 65–90). Two hubs in the default mode network exhibited less average controllability in old-aged participants. **c** Mean network average controllability was negatively associated with age in the default mode network (DefaultA), control network (ContB), and limbic network (LimbicB). In the group comparisons (Panel **b**) and the rank-correlations (Panel **c**), participant education was included as a covariate. The Bonferroni method for correction for multiple comparisons was applied to correct for the number of hubs analyzed (16) (Panel **b**). and the number of networks (17) (Panel **c**). *corrected *P* < 0.05, **corrected *P* < 0.001.

To begin our investigation of the relationship between network control and aging, we evaluated if the average controllability of control hubs in middle-aged participants (*n* = 305, ages 40–65) were different than in old-aged participants (*n* = 175, ages 65–90). We classified a node as a control hub if its average controllability was greater than one standard deviation above the mean average controllability for all nodes in middle-aged participants. This yielded 15 hubs, which were predominately within networks associated with cognitive function (Fig. 2a). These hubs were mostly consistent across global network thresholds of 0.001, 0.005, 0.010, 0.015 (Supplementary Tables S1–S4). However, the 15th hub DefaultB – PFCd_1 was only classified as a hub at lower global network thresholds of 0.001, and 0.005 (Supplementary Tables S1–S2). The distribution of hubs within the different large-scale networks (shown as percentages in Fig. 2a) were corrected by network size by normalizing the number of hubs in each network by their respective size^28^. Hubs of average controllability were most commonly within the default mode network (~40%), followed by the salience/ventral attention network (~25%), similar to previously reported results^28^. Next, we examined if average controllability for each of these identified hubs was different between middle- and old-aged participants (Fig. 2b). For the hubs with the greatest average controllability, the mean values were consistent across age groups. However, old-aged participants had less average controllability in two hubs within the default mode network (DefaultA – PFCm_4: *F* = 9.78, *p*_*bonf*._ = 0.028, DefaultB – PFCd_1: *F* = 28.13, *p*_*bonf*._ = 9.46e−08). The differences in average controllability between middle- and old-aged participants for the hub DefaultA – PFCm_4, remained significant at all thresholds tested (Supplementary Tables S1–S4), whereas the hub classification of DefaultB – PFCd_1 was sensitive to higher thresholds of 0.010, 0.015 (Supplementary Tables S3 and S4). To assess the extent to which the hubs were dependent on our choice of hub threshold, we performed an additional analysis identifying hubs as regions within the top 10% of average controllability, which corresponds to 40 nodes in our parcellation, and repeated the analysis with the global network thresholds of 0.001, 0.005, 0.010, 0.015 (Supplementary Tables S5–S8). The previously identified hub DefaultB – PFCd_1, was the only region to show significant differences between middle- and old-aged participants after correction for multiple comparisons.

### Declines in mean average controllability of large-scale networks are implicated in aging

Next, we investigated if average controllability showed age-associated differences at the level of large-scale networks. We calculated the mean average controllability for each of the 17 large-scale networks in our parcellation, and computed the ranked Spearman’s correlation with age. Age was negatively associated with mean average controllability in the default mode (DefaultB: Spearman’s ρ = −0.307, *p*_*bonf*._ = 1.15e−10), the frontoparietal control (ContB: Spearman’s ρ = −0.279, *p*_*bonf*._ = 8.92e−09), and the limbic (LimbicB: Spearman’s ρ = −0.223, *p*_*bonf*._ = 8.92e−09) networks (Fig. 2c and Supplementary Table S9). Significant associations between age and mean network average controllability were found for these networks across all global network thresholds tested (Supplementary Tables S9–S12). For the frontoparietal control network, this decline appeared to occur mostly before the age of 61 (Supplementary Fig. S1a), whereas for the default mode and limbic networks, these declines continued throughout the age range studied (Supplementary Fig. S1b, c, respectively).

### Minimal sex-related differences in mean network average controllability and relationships with age

Sex-related differences are crucial to understand when investigating the relationships between brain connectivity and aging. To evaluate the extent to which males and females differed in mean network average controllability, we performed ANCOVAs, with participant’s age and years of education included as covariates. In most networks, we did not observe sex-related differences. However, females showed lower mean network average controllability in the default mode (DefaultA: F = 13.68, *p*_*bonf*_. = 0.004) and the salience/ventral attention (SalVentAttnB: F = 10.79, *p*_*bonf*._ = 0.018) (Supplementary Table S13) networks. These results were consistent across thresholds assessed (Supplementary Tables S13–S16). Next, we assessed the degree to which associations between mean average network controllability and age differed between the sexes. We first computed sex-specific rank correlations between mean network average controllability and age for 17 networks. Then we computed the significance of these differences by computing the z-scored differences in correlation values using the Fischer’s z-transform. Males showed stronger negative relationships between age and mean network average controllability in the default mode (DefaultB) and frontoparietal control (ContB) networks, but no correlations were significantly different between the sexes across all thresholds assessed (Supplementary Tables S17–S20). Since we observed minimal sex-related differences in mean network average controllability, and no significant sex-related differences for the relationships between mean network average controllability and age for each of the 17 networks, we did not partition participants by sex in any of the following analyses.

### Nodal degree and redundancy show similar relationships to average controllability

To examine if multi-step connectivity was related to average controllability, we calculated network redundancy^42^, defined as number of non-circular paths between nodes up to a designated length *L* (*L* = 4 in our study*, see Methods*). We summed this measure at the node level, to get the total number of paths to and from each node for each participant’s structural brain network. Similarly, we calculated nodal degree for all nodes by summing the number of first-order edges that each node was involved in. Since redundancy is typically calculated on binary networks^42^, we calculated both degree and redundancy on binarized adjacency matrices where *A*_*ij*_ = 1 if a streamline existed between nodes *i* and *j*, else *A*_*ij*_ = 0. After computing nodal degree and nodal redundancy for all brain regions in the parcellation, we averaged these values, as well as the previously computed average controllability values for each node, across participants to get group level nodal degree, nodal redundancy, and nodal average controllability values. Next, we performed rank correlations between these network features and average controllability (Supplementary Fig. S2a). Both degree and redundancy showed strong relationships with average controllability (degree: Spearman’s ρ = 0.823, *p* = 1e−99, Supplementary Fig. S2a.i, redundancy: Spearman’s ρ = 0.804, *p* = 7e−92, Supplementary Fig. S2a.ii). To investigate the relationship between multi-step pathways and average controllability, we then performed a rank-correlation between degree-regressed redundancy and degree-regressed average controllability. Degree regressed redundancy and degree-regressed average controllability still maintained a positive relationship (Spearman’s ρ = 0.444, *p* = 1.1e−20, Supplementary Fig. S2b).

### Degree mediates differences in average controllability with age

**Fig. 3:**
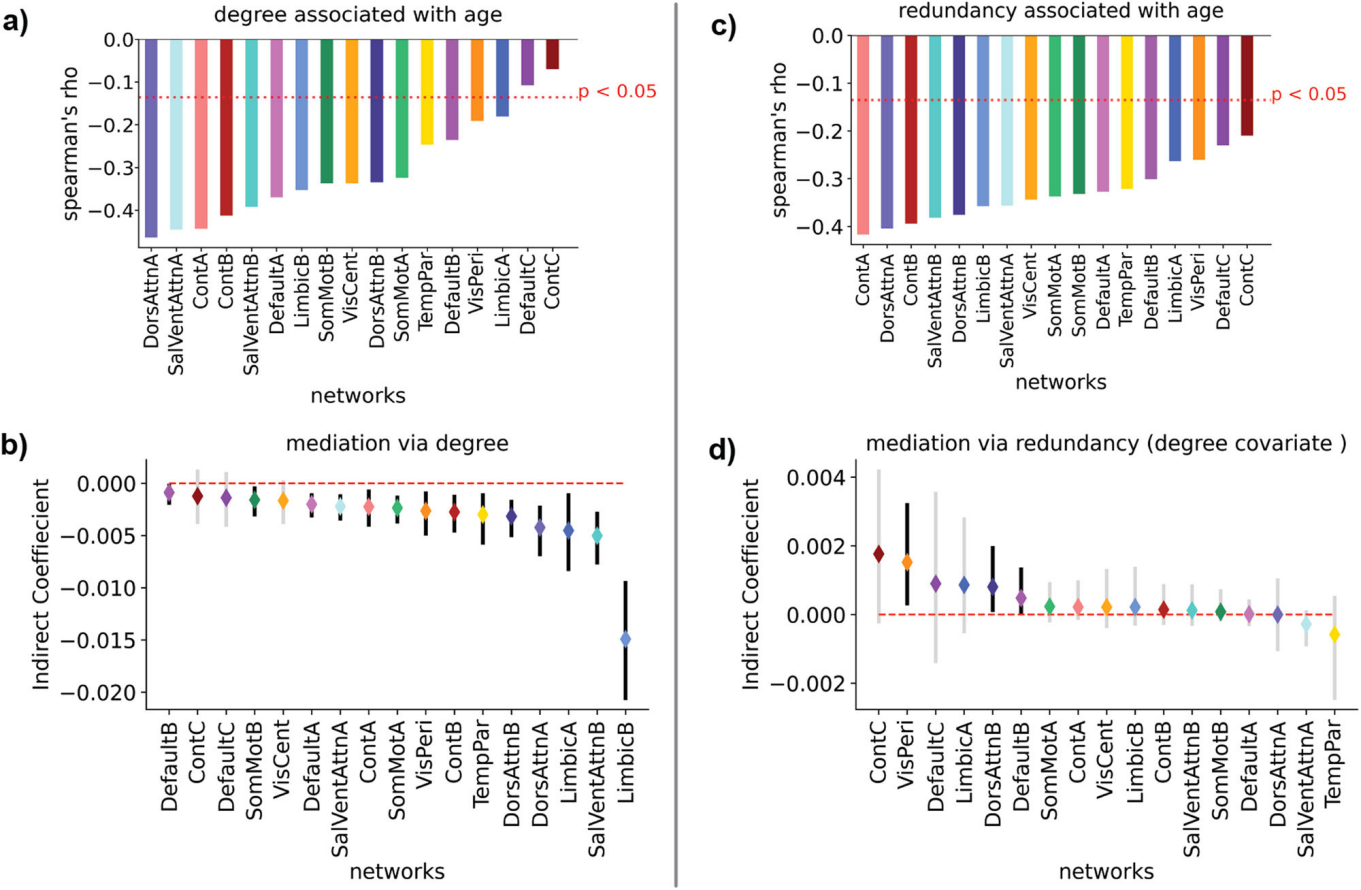
Multi-step connectivity (redundancy) mediates relationships between age and mean network average controllability over and above the effects of degree. **a** Average network degree was negatively associated with age for 15 of 17 large-scale networks. **b** Changes in degree mediated the relationship between age and mean network average controllability for 14 of 17 networks. **c** Average network redundancy also showed age associated declines, but for all networks examined. **d** Average network redundancy mediated relationships between age and average controllability for 3 of 17 networks when including degree as a covariate. These networks included the visual (VisPeri), dorsal attention (DorsAttnB), and default mode (DefaultB) networks. In each analysis participant education was included as a covariate. We used the Bonferonni method to correct for multiple comparisons. In each panel we corrected for the number of networks analyzed (17). In panels **b** and **d**, the mediation was significant if the confidence intervals did not cross 0 when the α = 0.05/17 to correct for multiple comparisons. Significant mediations are indicated by black confidence intervals, while non-significant mediations are indicated by grey confidence intervals.

Before assessing the influence of redundancy in age-associated differences in average controllability, we began by examining the importance of edges immediately connecting to nodes via degree. The average degree in 15 of 17 networks showed negative rank-correlations with age across all thresholds tested (*p*_*bonf*_ < 0.05, see Supplementary Tables S21–S24). For the threshold of 0.001(Fig. 3a), the strongest of these associations were in the dorsal attention network (DorsAttnA: Spearman’s = −0.464, *p*_*bonf*._ = 9.90e−26) and the salience/ventral attention network (SalVentAttnA: Spearman’s ρ = −0.446, *p*_*bonf*._ = 1.44e−23) (Supplementary Table S21). With the strong relationships between degree and average controllability, we expected that average network degree should influence the association between age and average controllability. We assessed this putative relationship by testing if degree mediated age-related differences in mean network average controllability. We performed mediation analyses for each of the 17 networks, and found that degree influenced the relationship between age and average controllability for 14 of 17 networks (all *p*_*bonf*._’s < 0.05, see Supplementary Table S17) (Fig. 3b), with the degree of the limbic network having the strongest impact on the relationship between its mean network average controllability and age (LimbicB, *β* = 0.0149, *p*_*bonf*._ < 1e−20). These 14 networks consistently mediated the relationship between mean network average controllability and age across thresholds (Supplementary Tables S25–S28).

### Redundancy mediates differences in average controllability with age over and above the effects of degree

We next turned towards examining the effects of redundancy in the relationship between age and average controllability. First, we calculated the average nodal redundancy for each network within our parcellation, and calculated a Spearman’s rank correlation with age. Similar to degree, average network redundancy shows widespread negative relationships with age (Fig. 3c). Redundancy in the frontoparietal control network showed the strongest negative relationship with age for the global threshold of 0.001 (ContA: Spearman’s *ρ* = −0.414, *p*_*bonf*._ = 5.26e−20, Supplementary Table S29), but all negative associations were significant across all thresholds (all *p*_*bonf*._’s < 0.05; see Supplementary Tables S29–32). Next, we investigated if the multi-step connectivity indexed by redundancy influenced the relationship between mean network average controllability and age, over and above the effects of degree. For each large-scale network in our parcellation, we performed a mediation analysis between age and average controllability with redundancy as the mediator, and included average degree of the respective large-scale networks as covariates. Redundancy mediated the relationship between age and mean network average controllability in 3 of 17 networks, over and above the effects of degree (all *p*_*bonf*._’s < 0.05, see Supplementary Table S5) (Fig. 3d). This included the default mode network (DefaultB: *β* = 0.0005, *p*_*bonf*._ < 0.014), which exhibited an age-associated decline in average controllability (Fig. 2c), the dorsal attention network (DorsAttnB: *β* = 0.0008, *p*_*bonf*._ < 0.010), and the visual network (VisPeri: *β* = 0.002, *p*_*bonf*._ < 1e−20). Of these networks, the mediations performed by redundancy were significant across all thresholds for the dorsal attention (DorsAttnB) and default model (DefaultB) networks (Supplementary Tables S33–S36).

### Average controllability and redundancy show moderate relationships in brain networks when controlling for participant age

Due to similarities in the computation of redundancy and average controllability, it is possible that the results from our mediation analyses are primarily driven by correlations between redundancy and average controllability, rather than empirical observations specific to age-associated variance in brain networks. To investigate this possibility, we performed partial correlations between mean network average controllability and average network redundancy, while controlling for average network degree, participant age, and education, across global network thresholds of 0.001, 0.005, 0.010, 0.015 (Supplementary Tables S37–S40). For the lowest threshold tested, 0.001 (Supplementary Table S37), we did not see significant relationships for any of the subnetworks that were identified in our mediation analyses (DefaultB, DorsAttnB, Supplementary Tables S33–S36). Moreover, only the default mode network (DefaultA) showed a relationship between mean network average controllability and average network redundancy, and this relationship was negative. For the second threshold, 0.005 (Supplementary Table S38), there were positive associations between redundancy and mean network average controllability in 4 of the 17 networks, and a negative association with the default mode network identified in the previous mediation analyses (DefaultB). For the remaining thresholds we observed positive relationships between mean network average controllability and average network redundancy in the dorsal attention network (DorsAttnB), but not in the aforementioned default mode network (DefaultB) (Supplementary Tables S39 and S40).

### Average controllability and redundancy are associated with processing speed

**Fig. 4:**
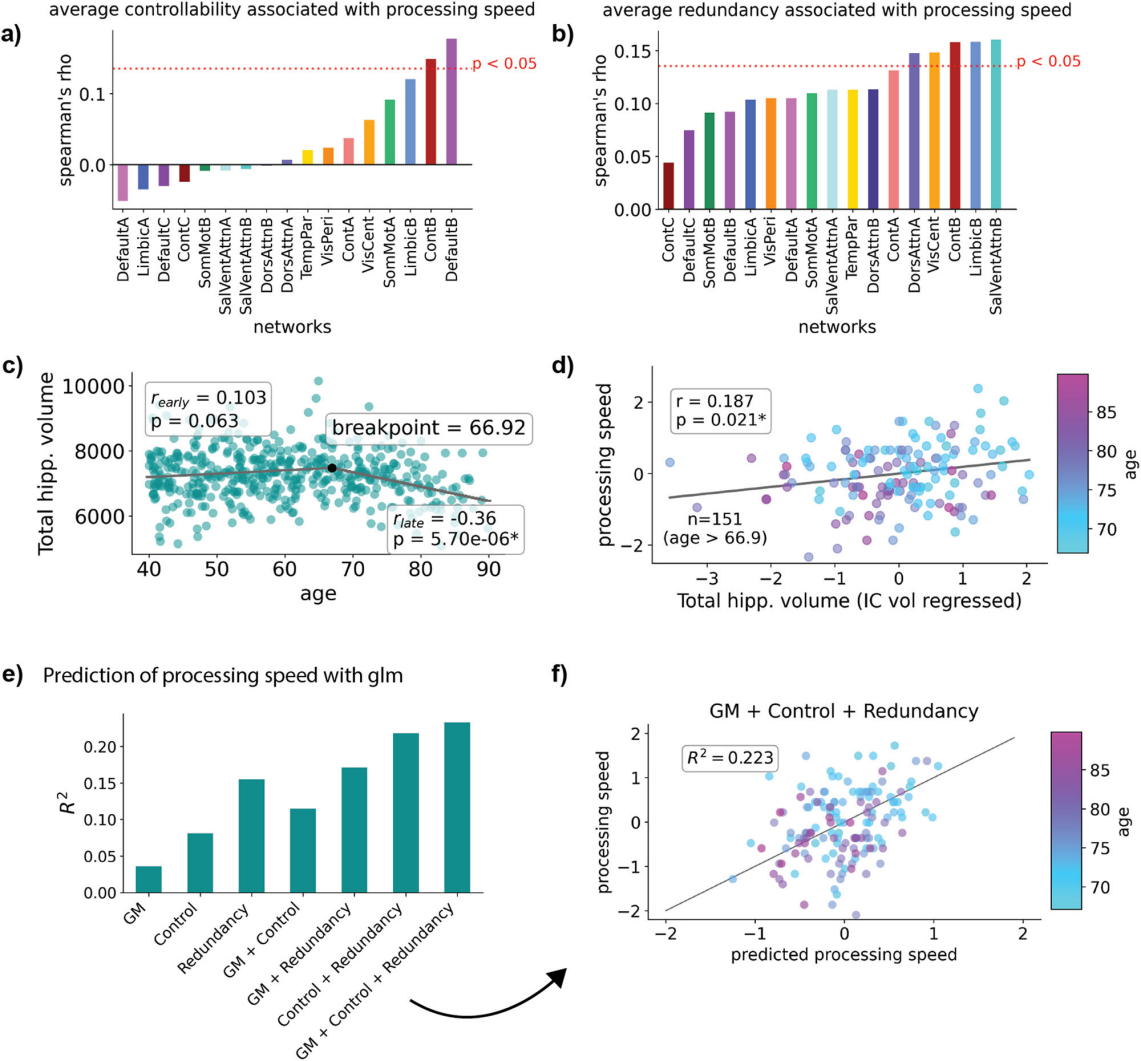
Associations between grey matter volume (GM), mean network average controllability, and redundancy, and processing speed. **a** Mean average controllability in the frontoparietal control (ContB), and default mode (DefaultB) networks was positively associated with processing speed. **b** Processing speed was positively associated with redundancy in 5 of 17 networks (all *p*_*bonf*._’s < 0.05). **c** Total hippocampal volume did not significantly change until around the age of 67, after which it showed a negative association with age. **d** For participants older than 66.92, IC volume-adjusted total hippocampal volume was positively associated with processing speed. **e** Performance of a general linear models when predicting processing speed with measures of GM volume, average controllability, and redundancy. For GM, we used IC-volume-adjusted measures of hippocampal volume, subcortical volume, and cortical volume. **f** The z-scored predicted processing speed versus real z-scored processing speed for the best model shown in **e**. In panels **a** and **b**, we used the Bonferroni method to correct for multiple comparisons based on the number of networks analyzed (17). For panels **d** and **e**, measures of processing speed and GM volume were z-scored. In the rank-correlations performed in panels **a** and **b**, participant education was included as a covariate.

After focusing on age-associated variance in average controllability and network properties that contribute to it, we studied the relationships between average controllability, redundancy, and cognitive function. We first associated the mean network average controllability for each large-scale network with processing speed, assessed by the Pattern Comparison Processing Speed Test^64^. We hypothesized that processing speed would be related to measures of influence on overall network dynamics, such as average controllability. We found that processing speed was positively associated with average controllability in the frontoparietal control (ContB: Spearman’s *ρ* = 0.144, *p*_*bonf*._ = 0.026) and the default mode (DefaultB: Spearman’s *ρ* = 0.174, *p*_*bonf*._ = 0.002) networks (Fig. 4a). These relationships were consistent across all thresholds examined (Supplementary Tables S41–S44). Next, we associated average network redundancy with processing speed. Redundancy in 6 of 17 large-scale networks was positively related to processing speed (all *p*_*bonf*._’s < 0.05) (Fig. 4b). This also included the frontoparietal control network (ContB: Spearman’s *ρ* = 0.168, *p*_*bonf*._ = 0.004), but redundancy in other networks was also positively associated with processing speed, including the salience/ventral attention (SalVentAttnB: Spearman’s *ρ* = 0.169, *p*_*bonf*._ = 0.004), limbic (LimbicB: Spearman’s *ρ* = 0.166, *p*_*bonf*._ = 0.005), visual (VisCent: Spearman’s *ρ* = 0.156, *p*_*bonf*._ = 0.01), and dorsal attention (DorsAttnA: Spearman’s *ρ* = 0.15, *p*_*bonf*._ = 0.008) networks. These networks exhibited significant positive relationships across all thresholds tested (Supplementary Tables S45–S48). However, other networks including the temporal parietal (TempPar), default mode (DefaultA), salience/ventral attention (SalVentAttnA) and somatomotor (SomMotA) networks also showed significant positive relationships at higher thresholds of 0.005, 0.010, and 0.015 (Supplementary Tables S46–S48).

### Hippocampal grey matter volume is positively associated with processing speed in older participants

Next, we investigated the association between hippocampal grey matter (GM) volume, one of the most commonly used measures of brain reserve, and processing speed. We only expected hippocampal volume to be a mechanism of brain reserve when declines in volume began in normal aging. To determine when decline starts within our participants, we used a piece-wise linear regression that identifies breakpoints in a data-driven manner. We found that hippocampal volume experiences a non-significant but positive trend between the ages of 40–66.92 (*r* = 0.103, *p* = 0.063), after which hippocampal volume in our participants showed significant age-associated decline (*r* = −0.36, *p* = 5.70e−06) (Fig. 4c). Total subcortical GM volume showed a similar trajectory, with a breakpoint at age 65 (Supplementary Fig. S3a), and total cortical GM volume showed continual decline throughout ages 40–90, with the most rapid decline occurring after age 75.33 (Supplementary Fig. S3b). Then, we used the residual method^65^ to determine if total hippocampal volume was a marker of cognitive reserve for participants with age >66.92. We found that total hippocampal volume, when adjusting for total intracranial volume^66^, was positively associated with processing speed in this older subset of subjects (*r* = 0.187, *p* = 0.021) (Fig. 4d).

### Controllability, redundancy, and grey matter volume are synergistically associated with cognitive performance

Finally, we examined if mean network average controllability, and GM volume served as complementary predictors of cognitive function in our participants. With the subset of participants older than the previously identified breakpoint for hippocampal volume (ages >66.92, *n* = 151, M/F = 67/84) we trained GLMs to predict processing speed using various combinations of GM volume, mean network average controllability, and average network redundancy for each of the 17 functional networks (Fig. 4e). For GM volume, we used total hippocampal volume, as well as subcortical and cortical volume. GM volume and average controllability did appear to have an almost entirely complementary effect on predicting cognition, yielding an *R*^2^ = 0.115, versus an *R*^2^ = 0.036 for GM alone, and *R*^2^ = 0.081 for average controllability alone. However, GM volume and redundancy showed better performance (*R*^2^ = 0.171), although there was more overlap in the predictive power between these features (redundancy: *R*^2^ = 0.155). The best model included all three sets of features (*R*^2^ = 0.233) (Fig. 4f and Table 1). These results were similar across all global network thresholds tested (Table 1, and Supplementary Tables S49–S51), for 0.001, 0.005, 0.010, and 0.015, respectively).

**Table 1:** GM, mean network average controllability (Control), and average network redundancy (Redundancy), each aid in the prediction of processing speed in older adults

Features	r^2^	Log likelihood	AIC	BIC
GM	0.036	−208.72	425.447	−581.872
Control	0.081	−205.1	446.191	−518.647
Redundancy	0.155	−198.87	433.747	−529.614
GM + Control	0.115	−202.33	446.654	−508.624
GM + Redundancy	0.171	−197.46	436.920	−516.969
Control + Redundancy	0.218	−193.14	459.279	−453.874
GM + Control + Redundancy	0.233	−191.66	459.320	−441.154

The R^2^, log-likelihood, AIC, and BIC for each GLM trained to predict processing speed in older participants (ages > 66.92, *n* = 151, M/F = 67/84) shown in Fig. 4e. Each set of features provided highly additive effects in the overall goodness-of-fit (R^2^) for these models. GM volume includes total hippocampal, cortical, and subcortical volume.

## Discussion

In this study we examined whether age-associated differences in the average controllability of brain networks is mitigated by redundancy. We found age-associated differences in the average controllability of structural networks within our functional parcellation in the default mode (DefaultB), frontoparietal control (ContB), and limbic (LimbicB) networks. Additionally, two control hubs within the default mode network showed declines in average controllability among old-aged participants. Furthermore, we investigated the extent to which these differences were influenced by the presence of single-step and multi-step pathways between brain regions. Degree, our measure of single-step connectivity, influenced age-associated differences in average controllability in 14 of the 17 functional networks. However, multi -step paths indicative of redundancy in the system^19,23^, mediated the relationships between age and average controllability in 3 of 17 networks, these included the visual (VisPeri), dorsal attention (DorsAttnB, SalVentAttnB), and default mode (DefaultB) networks. Finally, we investigated a previously posed hypothesis, that network controllability and GM volume, a more traditional measure of brain reserve, should each be partial proxies of cognitive function^36^. When using simple linear models, our results were consistent with this hypothesis. However, both redundancy and average controllability appeared to provide complementary predictive power when predicting the processing speed abilities of healthy older adults.

### Age related differences in average controllability

Structural networks reorganize in brain aging^16^. Despite these differences in network organization associated with age, mechanisms that mitigate these differences to maintain average controllability have been relatively understudied. In the present study, we evaluated differences in average controllability associated with aging in control hubs and in the structural connectivity of large-scale brain networks. We identified 15 hubs of average controllability, the first 14 of which were robust to threshold selection. The top 13 exhibited similar levels of average controllability between middle-aged and old-aged participants. While the 14th and 15th hubs, which were within the default mode network, exhibited less average controllability in old-aged participants. Many of these hubs were in the precuneus, and posterior cingulate, overlapping with previously identified average control hubs^28^, and regions identified as the structural core^67^. However, for two hubs in the default mode network, old-aged participants showed less average controllability than middle-aged participants. Both of these hubs were in the prefrontal cortex (PFC), one in the medial prefrontal cortex (PFCm), and the other in the dorsal prefrontal cortex (PFCd). The PFC experiences age-related declines in brain volume^68,69^ and white matter integrity^68^. Increased task-based brain activity in the PFC is commonly reported as a potential mechanism to compensate for declines in brain volume and white matter^44^ (for a review see refs. ^48,70^). Our results suggest that increased compensatory PFC activation could also be related to declines in the average controllability of hubs within the default mode network, providing further support to the possibility of network controllability as a measure linking brain and cognitive reserve^36^.

### Multi-step connectivity (redundancy) influences age-associated differences in average controllability

Nodal degree, a measure of the number edges connected to a particular node, has been shown to strongly predict nodal controllability within subjects^24,28,71–73^. However, the additional properties that influence controllability are largely unknown. In this study we investigated the relevance of redundant multi-step paths to brain network controllability^40–42^. We found that redundancy was positively associated with average controllability, when adjusted for degree, suggesting that multi-step pathways could play a crucial role in the control profiles of complex networks. Furthermore, our mediation analyses indicated that redundancy, while holding degree as a covariate, supported the average controllability of several key networks for cognitive function in aging. These included the default mode (DefaultB), dorsal attention DorsAttnB), and visual (VisPeri) networks. Of these networks, the default and limbic networks identified showed age-associated declines in average controllability, which indicates that redundancy could be a neuroprotective mechanism to mitigate these declines^43^. This work aligns with findings in other complex systems suggesting that edge redundancy can promote robust average controllability^24^, particularly in the context of changing network topologies, such as edge removal^50,51^, which is similar to weakening white matter connectivity observed in aging^74,75^. Our work suggests that the existence of multi-step pathways in brain networks could provide bridges of connectivity that preserve average network controllability^63^, to support dynamic brain activity in aging.

### Average controllability, redundancy, and processing speed

Processing speed in our study was assessed via the speed of pattern comparison^64^. This task requires several cognitive processes, such as visual search, working memory, and decision making. For both average controllability and redundancy, we found a positive association within the same subnetwork of the frontoparietal control network (ContB). The frontoparietal control network is important for allocation of attention, flexible goal-driven behavior, working memory, and decision making^76,77^. Furthermore, the global connectivity of the frontoparietal control network may allow it to influence brain-wide dynamics^78^. Our results suggest that the average controllability, and redundancy, of edges within the frontoparietal network could be important for enabling the diverse cognitive functions relevant in processing speed and other similar tasks. Furthermore, we found that processing speed was positively associated with redundancy in the dorsal attention (DorsAttnA), visual (VisCent), salience/ventral attention (SalVentAttnB) and limbic (LimbicB) networks, suggesting that increased number of communication pathways involving each of these networks could support guidance of top-down attention and discrimination in this visually-based processing speed task^79–82^.

### Complementary effects of grey matter volume, average controllability, and redundancy on cognitive performance

In support of the hypothesis that GM volume and network controllability could each be partial proxies of cognitive function^36^, we found that GM volume and average controllability had almost complementary effects on the goodness of fit for our model’s prediction of processing speed. GM volume and redundancy also improved performance together versus when considering either of them alone, but the additive effect on the goodness of fit was not as dramatic. This is not surprising, as hippocampal volume has been previously associated with redundancy in brain networks^23^. Additionally, we found that average controllability and redundancy showed highly complementary effects in predicting processing speed, despite similarities in their calculation. We propose that this is primarily due to the within-subject normalization performed when calculating average controllability which could mask age-associated variance between subjects. However, model performance was the best when including GM volume, average controllability, and redundancy in a single model, suggesting that they each could play an important role in cognitive function in healthy-aging.

### Mathematical considerations regarding the similarity between average controllability and redundancy

There are similarities in the computation of redundancy^42^ and average controllability^28^ that should be considered while interpreting our results. In particular, redundancy and average controllability can both be viewed as measuring the number of indirect paths associated with a node^83,84^. From this perspective, it is possible that redundancy mediating the relationship between age and mean network average controllability in several networks could be fundamentally a mathematical, rather than an empirical observation driven by age-associated variance in brain networks. However, when controlling for age within our participants, we did not find consistent relationships between average controllability and redundancy within the default mode network (DefaultB), suggesting that the observed indirect effect of DefaultB redundancy on the relationship between age and average controllability in this network is dependent on age-associated differences. Furthermore, if redundancy and average controllability indexed extremely similar features, their ability to predict processing speed should not have been as complementary as observed. There are two key differences in the computation of redundancy and average controllability. The first is that during the computation of average controllability, adjacency matrices are normalized by dividing by a constant plus the largest eigenvector. Second, average controllability approximates the diffusion of information across an infinite time scale, whereas redundancy is finite. Infinite time-scale diffusion on other message-passing systems has been proven to cause over-smoothing^85^, leading to a steady state that primarily consists of information about the number of connected components^86^ and the loss of information related node-specific features^85,87,88^. This over-smoothing problem is present in controllability measures, as well as measures of communicability^89^, and other metrics that consider paths of infinite length on a network.

### Greater mean network average controllability in males within the default mode and salience/ventral attention networks

Males and females exhibit different white matter network connectivity topologies in development^90,91^, adulthood^92,93^, and in aging^94^. In development, white matter differences contribute to differing network controllability profiles that are predictive of poorer executive function in males^90^. In adulthood, differing hemispheric connectivity contributes to males having higher average controllability in regions involving motor and auditory function, which could facilitate motor activation ^93^. In our study, males had higher mean network average controllability of the default mode (DefaultA), and salience/ventral attention (SalVentAttnB) networks of middle- and old-aged adults. We did not find that average controllability in these networks was related to processing speed, the only cognitive function assessed in this study. However, future studies could investigate if these differences are associated with measures of executive function in aging, where females tend to show more initial reserve, but faster decline after a tipping point is reached^95,96^.

### Limitations and future directions

The goals of our study included assessing the extent to which redundancy could mitigate age-associated differences in network control, and evaluate network control in the context of traditional measures of brain reserve. We used mediation analyses within our study which relied on a cross-sectional sample. Cross-sectional age-associated variance does not always hold in longitudinal settings^97^, thus replication of our findings in a longitudinal setting would be ideal. When studying a traditional measure of reserve, we used the residual method^65^ to assess if increased hippocampal volume was positively associated with processing speed. While we did observe hippocampal atrophy (reduction in GM volume) in older participants, this method is primarily used in the context of neurodegenerative diseases^65^. Future studies may consider including participants with later stages of dementia-associated atrophy to further evaluate network controllability in the context of reserve. Another limitation of our study is that we performed limited investigation into sex-related differences in our results. Our initial analyses in this direction yielded minimal sex-related differences in mean network average controllability, and no significant differences in the relationships between mean network controllability and age for each of the 17 networks. Future work could expand on these results to consider if differences in network controllability drive observed sex-related differences in the rates of cognitive decline^95,96^, particularly for executive function, which has been associated with faster degradation of white matter tracts in females^94^. Finally, while we focused on average controllability in this study, future work could expand upon existing studies of modal controllability in aging, to further our understanding of how age-associated changes in network topology influence network controllability, and the relevance of these changes in cognitive and brain reserve.

## Methods

### Dataset and participants

We used preprocessed dMRI data obtained from the 2.0 release of the Human Connectome Project – Aging database^61^. Ages of participants ranged from 40 to 90 (*n* = 646), of these we used data from a subset of 545 participants with valid cognitive assessments for the cognitive scores we used in our study. We restricted participants to those with normal cognitive function as assessed by the Montreal Cognitive Assessment (MoCA)^98^. For subjects older than 65, we used a cutoff of 23/30, which has been found to limit false diagnosis of mild cognitive impairment^99^. There are forms of dementia that the MoCA can be insensitive to, such as vascular dementia^100^, semantic dementia^101^ and frontotemporal dementia^102^, each with differing patterns of cognitive impairment. To reduce the likelihood of inclusion of participants with early stages of these forms of dementia, we also excluded subjects between the ages of 65–90 with poor performance on measures of cognitive flexibility^103,104^, vocabulary comprehension^103,105,106^, and executive function^17,103,104,107^. Poor performance was defined as a performance level worse than two standard deviations below the mean. These restrictions removed another 65 participants from eligibility, leaving us with data from 480 (281 females, 199 males) participants for this study. All participants gave written informed consent and all procedures had been pre-approved by local Institutional Review Boards. All ethical regulations relevant to human research participants were followed.

### Image acquisition and processing

T1-weighted structural images were acquired in a 3 Tesla Siemens Prisma Scanner. A multi-echo magnetization prepared rapid gradient echo (MPRAGE) sequence (voxel size: 0.8 × 0.8 × 0.8 mm, TE = 1.8/3.6/5.4/7.2 ms, TR = 2500 ms, flip angle = 8 degrees) was used. Diffusion MRI (dMRI) images were generated from multi-shell diffusion with b-values of 1500 and 3000 s/mm^2^, with 93 and 92 sampling directions, a slice thickness of 1.5 mm, and an in-plane resolution of 1.5 mm. We used preprocessed dMRI data for our study. For details on the preprocessing pipeline see: https://brain.labsolver.org/hcp_a.html. Briefly, the pipeline involved susceptibility artifact detection with the TOPOP, from the Tiny FSL package (http://github.com/frankyeh/TinyFSL), alignment with the AC-PC line, restricted diffusion imaging^108^, and generalized q-sampling^109^. These analyses were conducted at Extreme Science and Engineering Discovery Environment (XSEDE)^110^ resources using the allocation TG-CIS200026.

### Network construction

Preprocessed dMRI data was reconstructed in DSI Studio (http://dsi-studio.labsolver.org). We performed whole-brain fiber tracking with 5,000,000 streamlines. (Fig. 1a). Structural networks were constructed according to the Schaefer Local-Global cortical parcellation with 400 cortical regions^62^, which subdivides the human cortex into 17 large-scale (functional) networks (Fig. 1b). Each brain parcel was considered a node, with the number of streamlines between any pair of parcels used as the weighted edge. Edges were removed if they were below 0.001 of the maximum edge weight per network. Additionally, we repeated major analyses with thresholds of 0.005, 0.01, 0.015, to assess the robustness of our results.

### Average controllability calculations

Average controllability, defined as the average energy from a set of control nodes on dynamic state trajectory over all possible states, was calculated using the trace of the finite time controllability Gramian^111^. The finite time controllability Gramian is computed via:

1 $${W}_{k}={\sum }_{t=0}^{\infty }{A}^{t}{B}_{k}{B}_{k}^{t}{A}^{t}$$

Where *A* is the adjacency matrix, normalized by dividing my one-plus the largest absolute eigenvector, ${B}_{k}$ is an input matrix of dimension 1 × *nROIs*, and *k* represents the set of nodes specified as control nodes. In our study we calculated the average controllability of each of the nodes within the 400 node parcellation. In several analyses we averaged these values at the level of large-scale networks, which we referred to as the *mean average controllability* of the respective large-scale network.

### Redundancy calculations

Redundancy was calculated as the number of simple (non-circular) paths between a pair of nodes up to a specified length (here we used *L* = 4)^42^, according to the equation:

2 $${R}_{i,j}={\sum }_{k=1}^{L}P\left(i,j,k\right).$$

Where *P*(*i, j, k*) was the number of paths non-circular paths between nodes *i*, and *j*, calculated with the *all_simple_paths* function in NetworkX^112^. To get nodal redundancy, we summed the total number of paths from each node to all other nodes. After calculation of nodal redundancy for all nodes in each subject’s structural networks, we calculated the average redundancy in the structural connectivity of each of the 17 large-scale networks per subject. We used the binarized structural connectivity matrices for these calculations.

### Cognitive measures

We focused on the cognitive measure of processing speed within our study because processing speed is believed to be limited by communication along white-matter tracts^52,53^. Processing speed was assessed via the Pattern Comparison Processing Speed Test^64^. Subjects were shown pairs of objects and asked to judge whether two objects, presented simultaneously, were the same or different. They were given 85 s to judge as many objects as possible. We used participant’s MoCA scores to determine if they were healthy (score >= 23/30). Additionally, we used measures of cognitive flexibility, assessed via the Dimensional Card Sort Test^113^, executive control, assessed via the Flanker Inhibitory Control and Attention Test^114^, and vocabulary comprehension, assessed via the Picture Vocabulary Test^107^, to exclude subjects with forms of dementia that the MoCA is insensitive to^17^.

### Grey matter volume extraction

From the T1-weighted images, we extracted grey matter volume using the *run_first_all* command within Freesurfer^115^. This included extraction of hippocampal volume, and the volume of subcortical structures, in the Aseg atlas^116^, as well cortical grey matter volume and estimated total intracranial volume.

### Statistics and reproducibility

We performed group comparisons of average controllability in middle-aged (40 ≤ age < 65, *n* = 305, M/F = 120/185) and old-aged (65 ≤ age < 90, *n* = 175, M/F = 79/96) adults. We performed rank-correlations between features of network controllability and redundancy with age and processing speed, as well as rank-correlations between redundancy, degree, and network controllability measures. We then performed parallel mediation analyses to investigate the effects of degree and redundancy on the relationships between age and average controllability. In the mediation analyses with redundancy, ranked-degree was included as a covariate to highlight influence of multi-step pathways on the relationships between age and average controllability. For all mediation analyses, participant education was included as a covariate. Inferential statistics were estimated with 10,000 bootstraps. The correlations and mediation analyses were performed using the entire sample of participants (*n* = 480, M/F = 199/281). Following these experiments, we performed a breakpoint analysis using a piece-wise linear regression to determine the starting point of hippocampal atrophy in our healthy cross-sectional sample. Using participants older than the discovered breakpoint (age >66.92, *n* = 151, M/F = 67/84), we used general linear models (GLMs) to investigate the extent to which linear combinations of GM and network features aided in the prediction of processing speed. ANCOVAs, Spearman’s correlations, Pearson’s correlations, and mediation analysis, were performed using the python package Pingouin^117^. Welch’s ANOVAs were used to compare average controllability in 15 identified hubs in middle-aged and old-aged adults. We used the Bonferroni method to correct for multiple comparisons which set the p-value necessary for significance to *p* < 0.05/15. For Spearman and Pearson correlations, we required *p* < 0.05/17 to correct for the number of functional networks analyzed. Mediation analyses were performed with the *α* = 0.05/17 for the confidence intervals, with significance determined by whether or not the confidence intervals for each coefficient crossed the value of zero. Piece-wise linear regression to determine breakpoints in rates of change for grey matter volume was performed using the pwlf python package^118^. Our GLMs were constructed using the python package Statsmodels^119^. Additional stats derived from these models (*R*^2^, log-likelihood, AIC^120^, BIC^121^) were also computed using the Statsmodels package.

### Plotting

We used custom python scripts for plotting and data visualization based on the Matplotlib^122^, Pandas^123^, and Seaborn^124^, packages.

### Supplementary information

Supplementary Information

ReportingSummary

## Data Availability

All data used in this study is publicly available via the Human Connectome Project – Aging dataset^61^.

## Code availability

Supporting code for statistical analyses can be found here: https://github.com/WilliamStanford/ControllabilityInAging/.
