# Supplementary material for: Age-related differences in network controllability are mitigated by redundancy in large-scale brain networks
Source: Commun Biol. 2024 Jun 7;7:701. doi: 10.1038/s42003-024-06392-2 (PMC11161655; doi:10.1038/s42003-024-06392-2)
Supplement: Supplementary file 1 — Supplementary Information [file 42003_2024_6392_MOESM1_ESM.docx]

Supplementary Information for

**Age-related differences in network controllability are mitigated by redundancy in large-scale brain networks**

William Stanford *et al.*

Corresponding author. Email: [eran_dayan@med.unc.edu](mailto:eran_dayan@med.unc.edu)

**This PDF file includes:**

Figures. S1 to S3

Tables S1 to S51


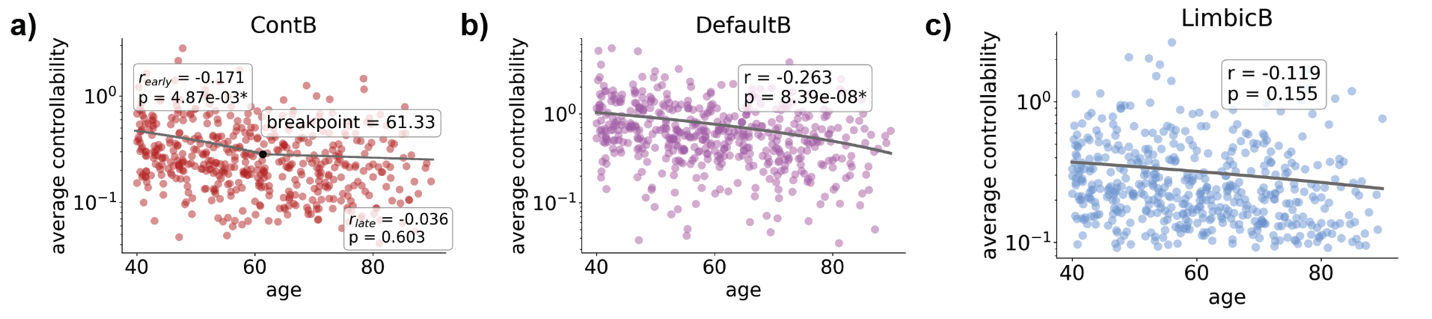


**Fig. S1. Scatter plots of age versus the mean average controllability for networks that showed negative rank correlations with age.** For the purpose of illustrating the raw values, we report Pearson correlations here, whereas the rank correlations are reported in the main paper. **a)** The frontoparietal control network (ContB) only showed decline between the ages of 40-61, afterwards the rate of decline was not significant from zero. **b)** The default mode network (DefaultB) showed a negative relationship with age throughout the entire age-range studied. **c)** The limbic network (LimbicB), did not show a significant linear correlation, despite the significant rank correlation observed in the main text.


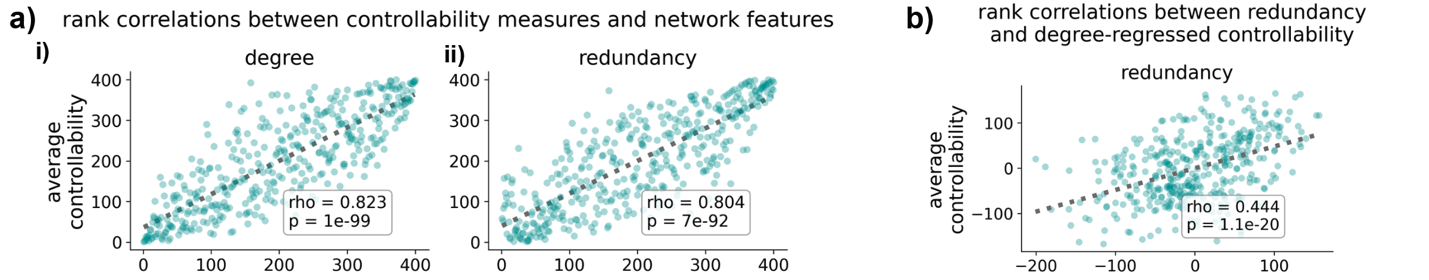


**Fig. S2. Redundancy relates to average controllability over and above the effects of degree.** **a)** Nodal degree and redundancy showed similar relationships with nodal measures of average controllability. **b)** Redundancy was still positively associated with average controllability when regressing out the effects of degree from nodal average controllability and nodal redundancy.


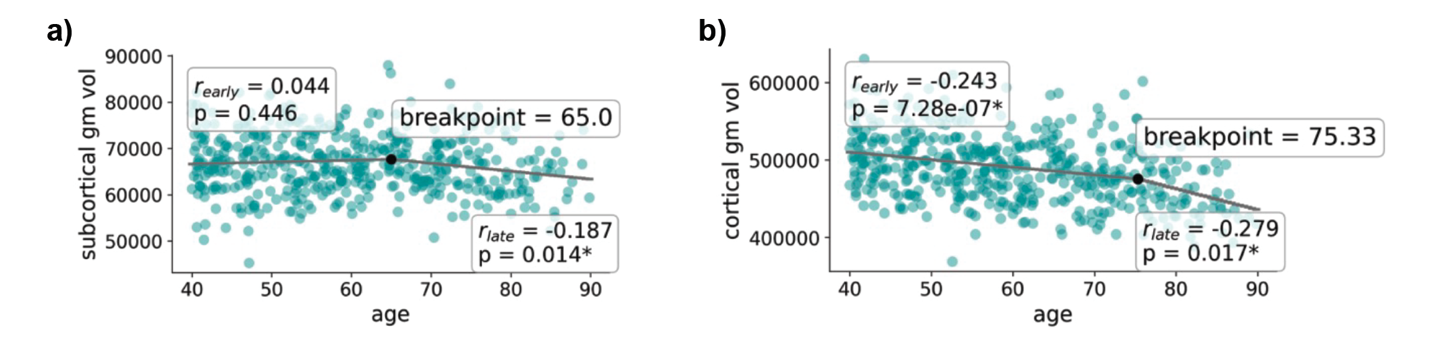


**Fig S3. Age-associated changes in subcortical and cortical grey matter volume.** **a)** Subcortical grey matter volume showed no age-associated declines until age 65, afterwards it was negatively associated with age. **b)** Cortical grey matter volume experienced age-associated decline across the entire age-range studied, but declined more quickly after the age of ~75. We used a piece-wise regression that determines the break-point in a data-driven manner.

**Supplementary tables S1-S51**

**
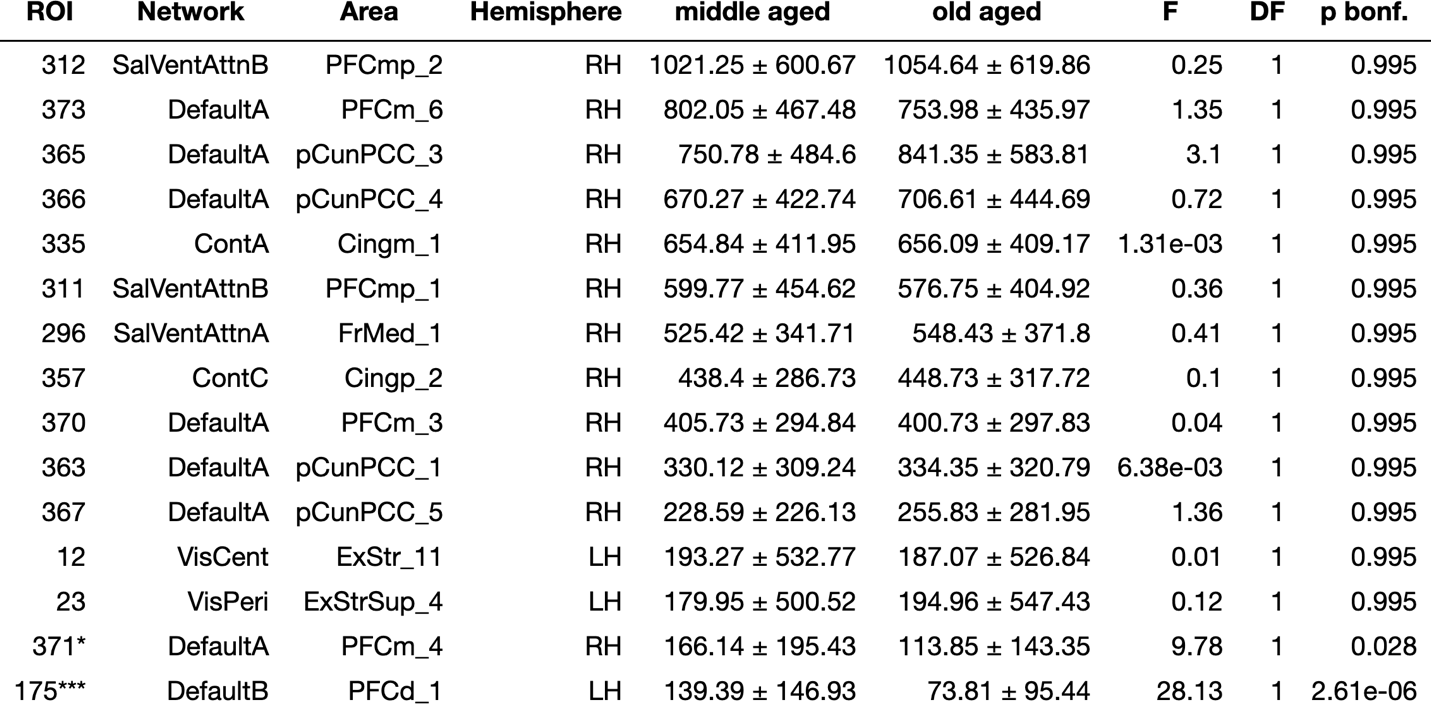
**

**Table S1. Differences in average controllability of hubs between middle and old aged adults, with global network thresholds of 0.001, and hubs determined by mean average controllability > mean ± std. of average controllability for all ROIs.** For each ROI, the means in the average controllability for the two groups are indicated, as well as their standard deviations (µ ± σ). Significance was assessed with ANCOVAs, with years of education included as a covariate. The Bonferroni method was applied to correct for the number of hubs compared (15), the corrected p-value is indicated by “p bonf.”, *corrected p bonf. < 0.05, **p bonf*.*  < 0.001, ***p bonf. < 1e-05.


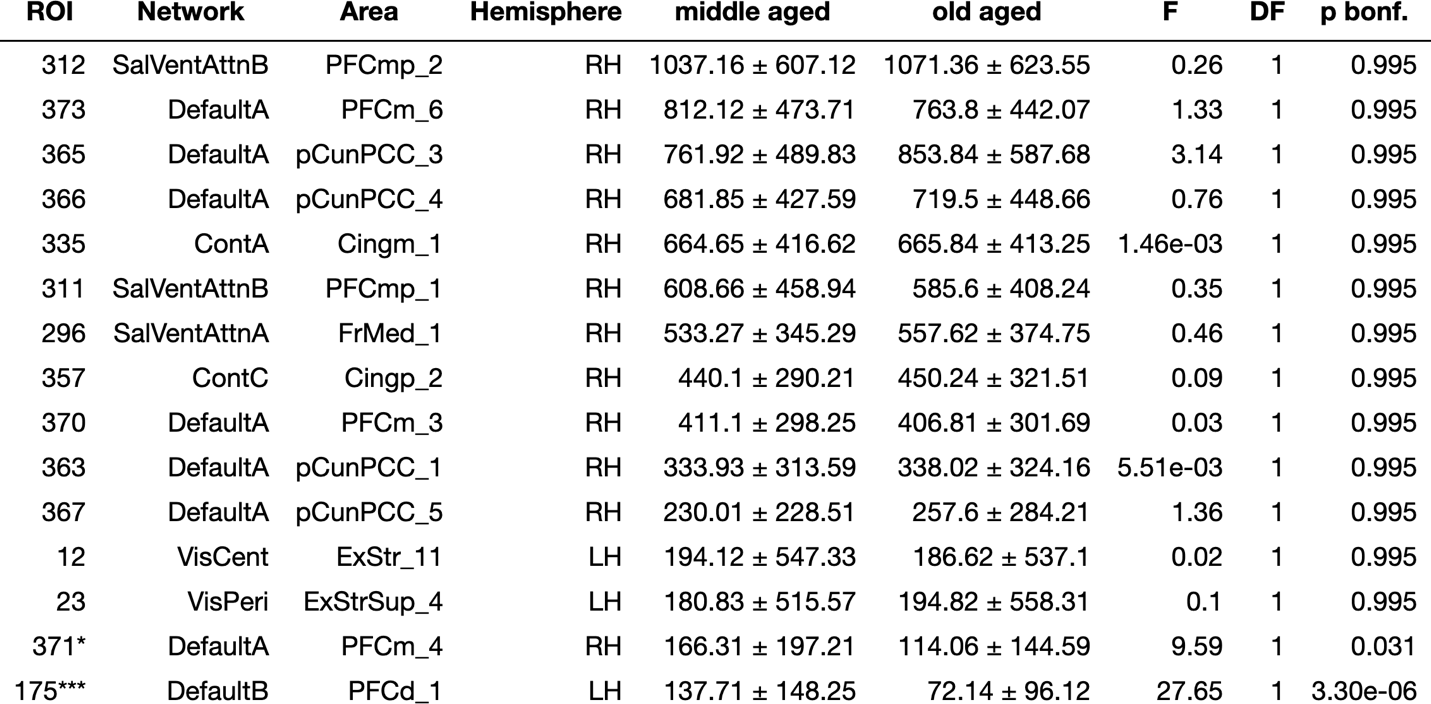


**Table S2. Differences in average controllability of hubs between middle and old aged adults, with global network thresholds of 0.005, and hubs determined by mean average controllability > mean ± std. of average controllability for all ROIs.** For each ROI, the means in the average controllability for the two groups are indicated, as well as their standard deviations (µ ± σ). Significance was assessed with ANCOVAs, with years of education included as a covariate. The Bonferroni method was applied to correct for the number of hubs compared (15), the corrected p-value is indicated by “p bonf.”, *corrected p bonf. < 0.05, **p bonf*.*  < 0.001, ***p bonf. < 1e-05.


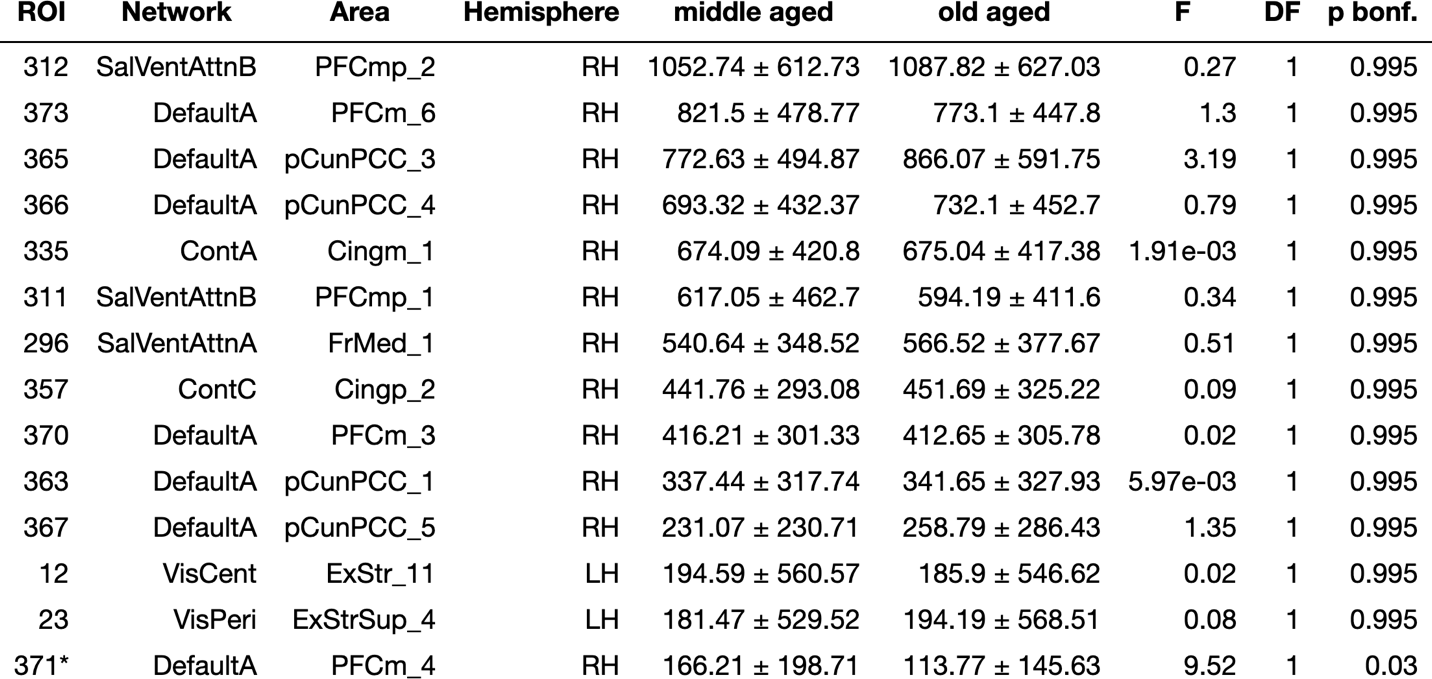


**Table S3. Differences in average controllability of hubs between middle and old aged adults, with global network thresholds of 0.010, and hubs determined by mean average controllability > mean ± std. of average controllability for all ROIs.** For each ROI, the means in the average controllability for the two groups are indicated, as well as their standard deviations (µ ± σ). Significance was assessed with ANCOVAs, with years of education included as a covariate. The Bonferroni method was applied to correct for the number of hubs compared (14), the corrected p-value is indicated by “p bonf.”, *corrected p bonf. < 0.05, **p bonf*.*  < 0.001, ***p bonf. < 1e-05.


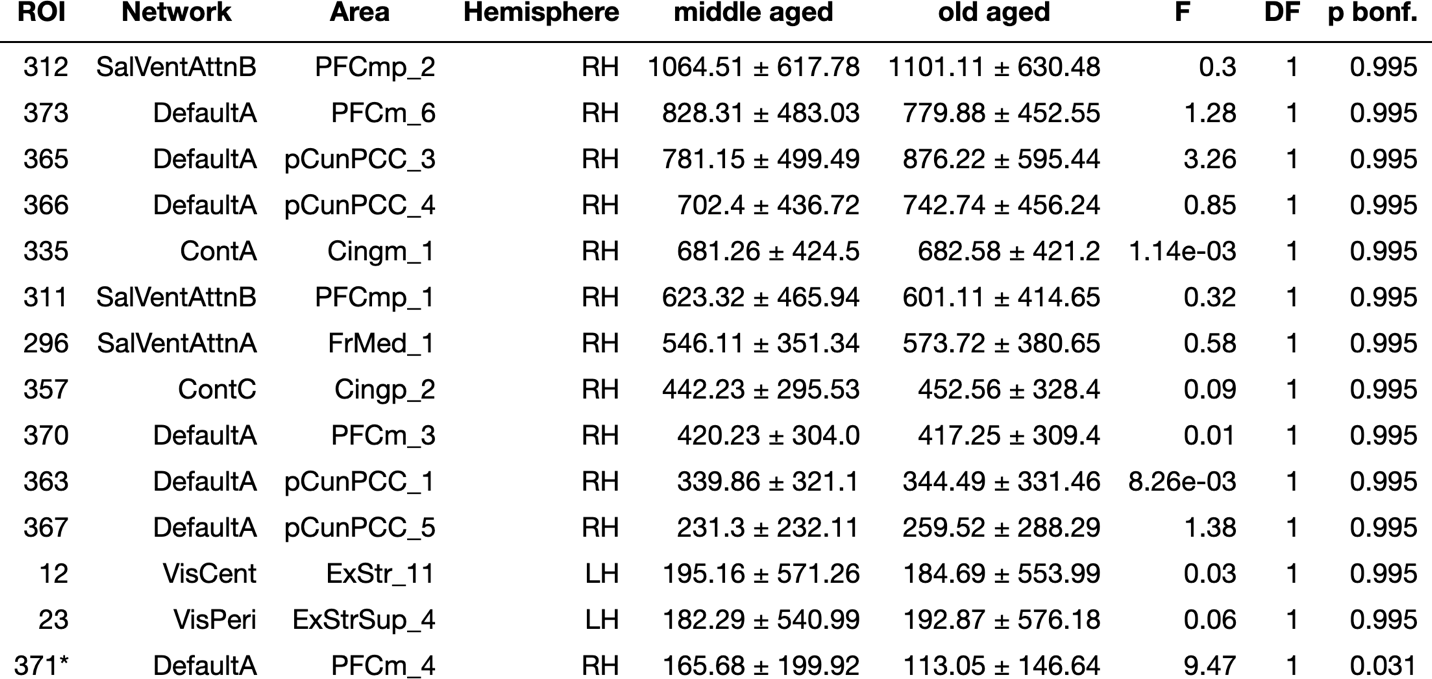


**Table S4. Differences in average controllability of hubs between middle and old aged adults, with global network thresholds of 0.015, and hubs determined by mean average controllability > mean ± std. of average controllability for all ROIs.** For each ROI, the means in the average controllability for the two groups are indicated, as well as their standard deviations (µ ± σ). Significance was assessed with ANCOVAs, with years of education included as a covariate. The Bonferroni method was applied to correct for the number of hubs compared (14), the corrected p-value is indicated by “p bonf.”, *corrected p bonf. < 0.05, **p bonf*.*  < 0.001, ***p bonf. < 1e-05.


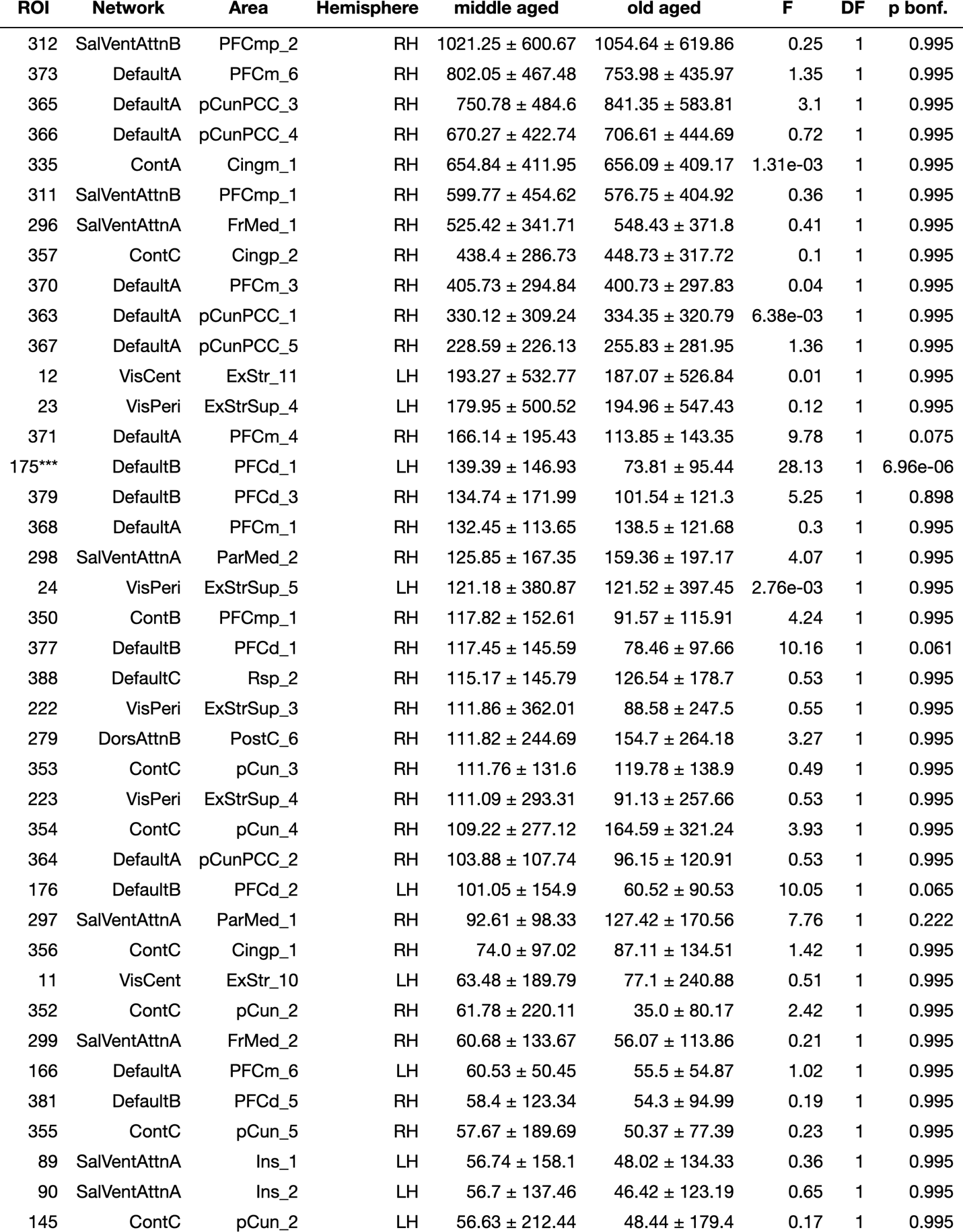


**Table S5. Differences in average controllability of hubs between middle and old aged adults, with global network thresholds of 0.001, and hubs determined if mean average controllability of an ROI was in the top 10% mean average controllability for all ROIs.** For each ROI, the mean average controllability for each group is indicated, as well as their standard deviations (µ ± σ). Significance was assessed with ANCOVAs, with years of education included as a covariate. The Bonferroni method was applied to correct for the number of hubs compared (40), the corrected p-value is indicated by “p bonf.”, *corrected p bonf. < 0.05, **p bonf*.*  < 0.001, ***p bonf. < 1e-05.

**
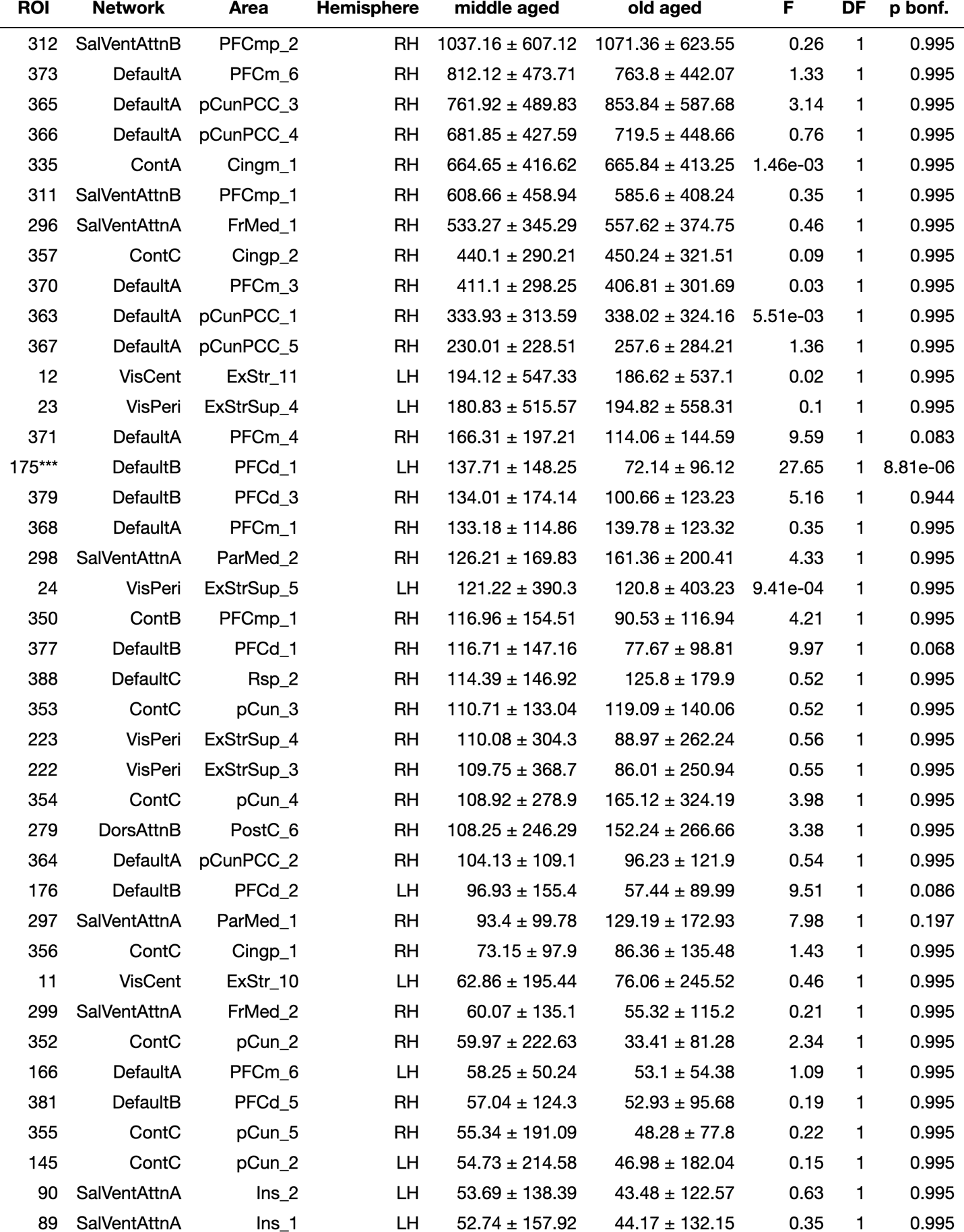
**

**Table S6. Differences in average controllability of hubs between middle and old aged adults, with global network thresholds of 0.005, and hubs determined if mean average controllability of an ROI was in the top 10% mean average controllability for all ROIs.** For each ROI, the means in the average controllability for the two groups are indicated, as well as their standard deviations (µ ± σ). Significance was assessed with ANCOVAs, with years of education included as a covariate. The Bonferroni method was applied to correct for the number of hubs compared (40), the corrected p-value is indicated by “p bonf.”, *corrected p bonf. < 0.05, **p bonf*.*  < 0.001, ***p bonf. < 1e-05.

**
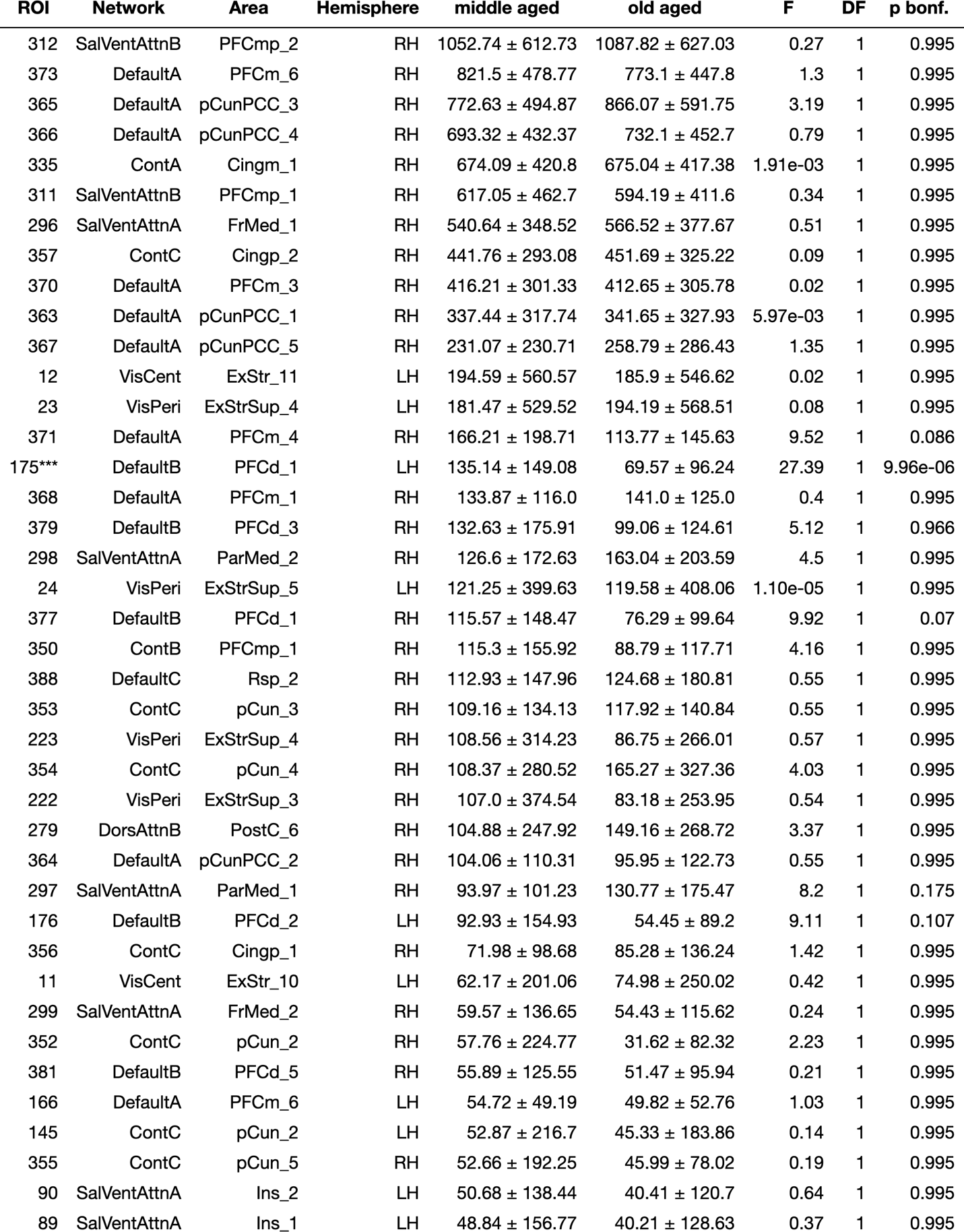
**

**Table S7. Differences in average controllability of hubs between middle and old aged adults, with global network thresholds of 0.010, and hubs determined if mean average controllability of an ROI was in the top 10% mean average controllability for all ROIs.** For each ROI, the means in the average controllability for the two groups are indicated, as well as their standard deviations (µ ± σ). Significance was assessed with ANCOVAs, with years of education included as a covariate. The Bonferroni method was applied to correct for the number of hubs compared (40), the corrected p-value is indicated by “p bonf.”, *corrected p bonf. < 0.05, **p bonf*.*  < 0.001, ***p bonf. < 1e-05.


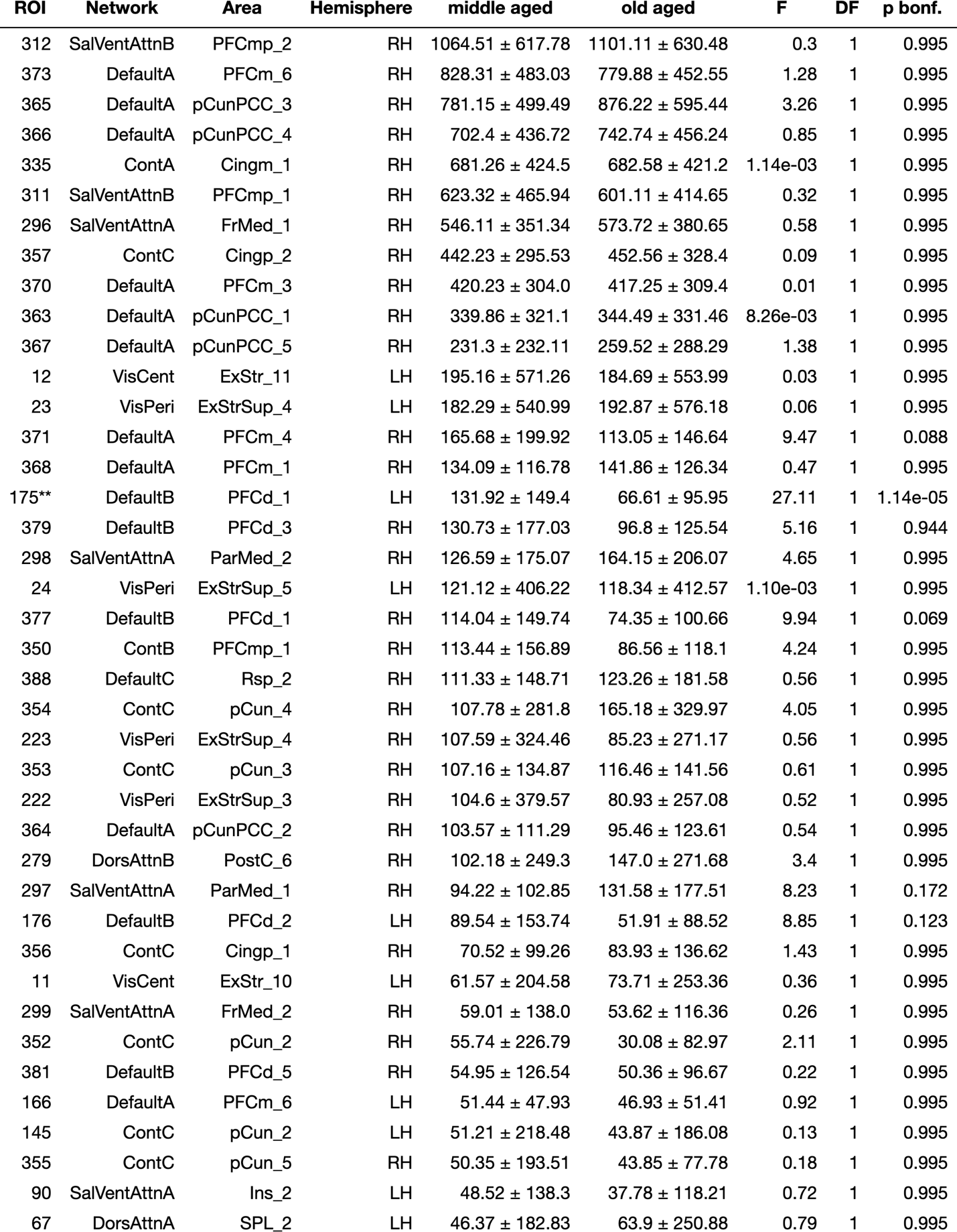


**Table S8. Differences in average controllability of hubs between middle and old aged adults, with global network thresholds of 0.015, and hubs determined if mean average controllability of an ROI was in the top 10% mean average controllability for all ROIs.** For each ROI, the means in the average controllability for the two groups are indicated, as well as their standard deviations (µ ± σ). Significance was assessed with ANCOVAs, with years of education included as a covariate. The Bonferroni method was applied to correct for the number of hubs compared (40), the corrected p-value is indicated by “p bonf.”, *corrected p bonf. < 0.05, **p bonf*.*  < 0.001, ***p bonf. < 1e-05.


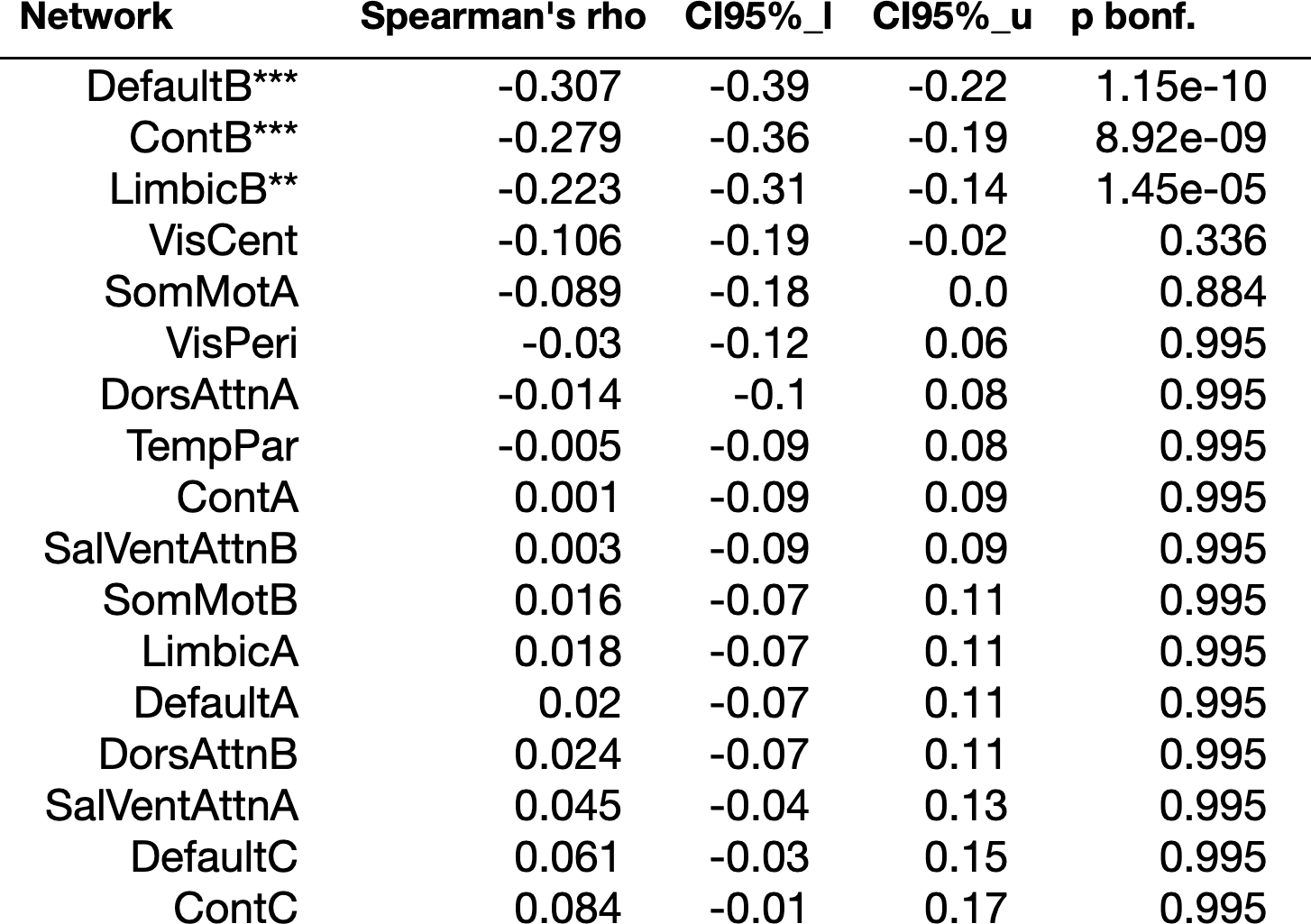


**Table S9. Mean network average controllability associated with age, with global network threshold of 0.001 the maximum streamline count per subject.** The mean network average controllability of the default mode (DefaultB), frontoparietal control (ContB), and Limbic (LimbicB) networks was negative associated with age. Education was included as a covariate for all associations. Networks are sorted in ascending order by the calculated Spearman’s ρ’s. The Bonferroni method was used to correct for multiple comparisons. *corrected p bonf. < 0.05, **p bonf*.*  < 0.001, ***p bonf. < 1e-05.

**
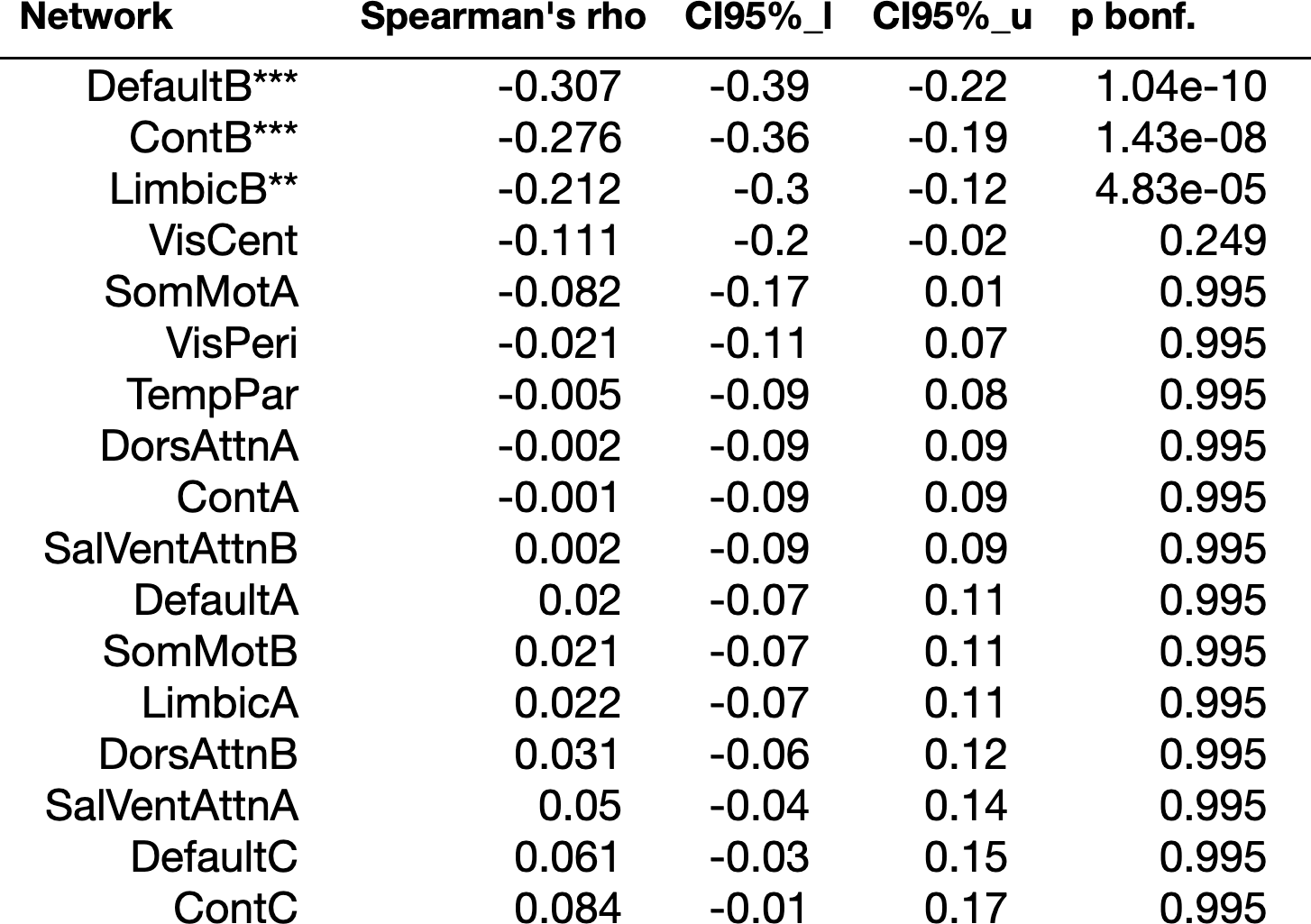
**

**Table S10. Mean network average controllability associated with age, with global network threshold of 0.005 the maximum streamline count per subject.** The mean network average controllability of the default mode (DefaultB), frontoparietal control (ContB), and Limbic (LimbicB) networks was negative associated with age. Education was included as a covariate for all associations. Networks are sorted in ascending order by the calculated Spearman’s ρ’s. The Bonferroni method was used to correct for multiple comparisons. *corrected p bonf. < 0.05, **p bonf*.*  < 0.001, ***p bonf. < 1e-05.

**
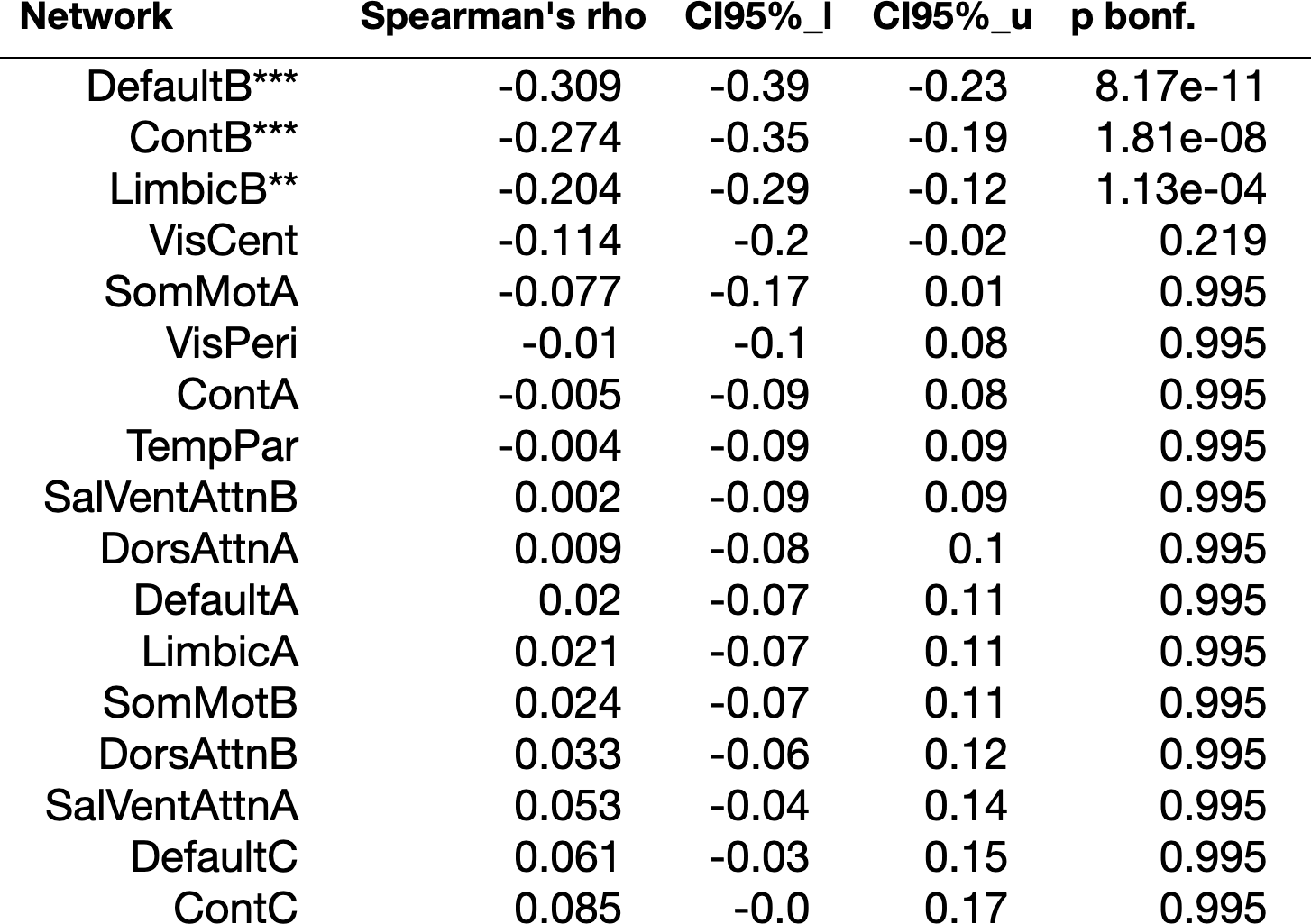
**

**Table S11. Mean network average controllability associated with age, with global network threshold of 0.010 the maximum streamline count per subject.** The mean network average controllability of the default mode (DefaultB), frontoparietal control (ContB), and Limbic (LimbicB) networks was negative associated with age. Education was included as a covariate for all associations. Networks are sorted in ascending order by the calculated Spearman’s ρ’s. The Bonferroni method was used to correct for multiple comparisons. *corrected p bonf. < 0.05, **p bonf*.*  < 0.001, ***p bonf. < 1e-05.

**
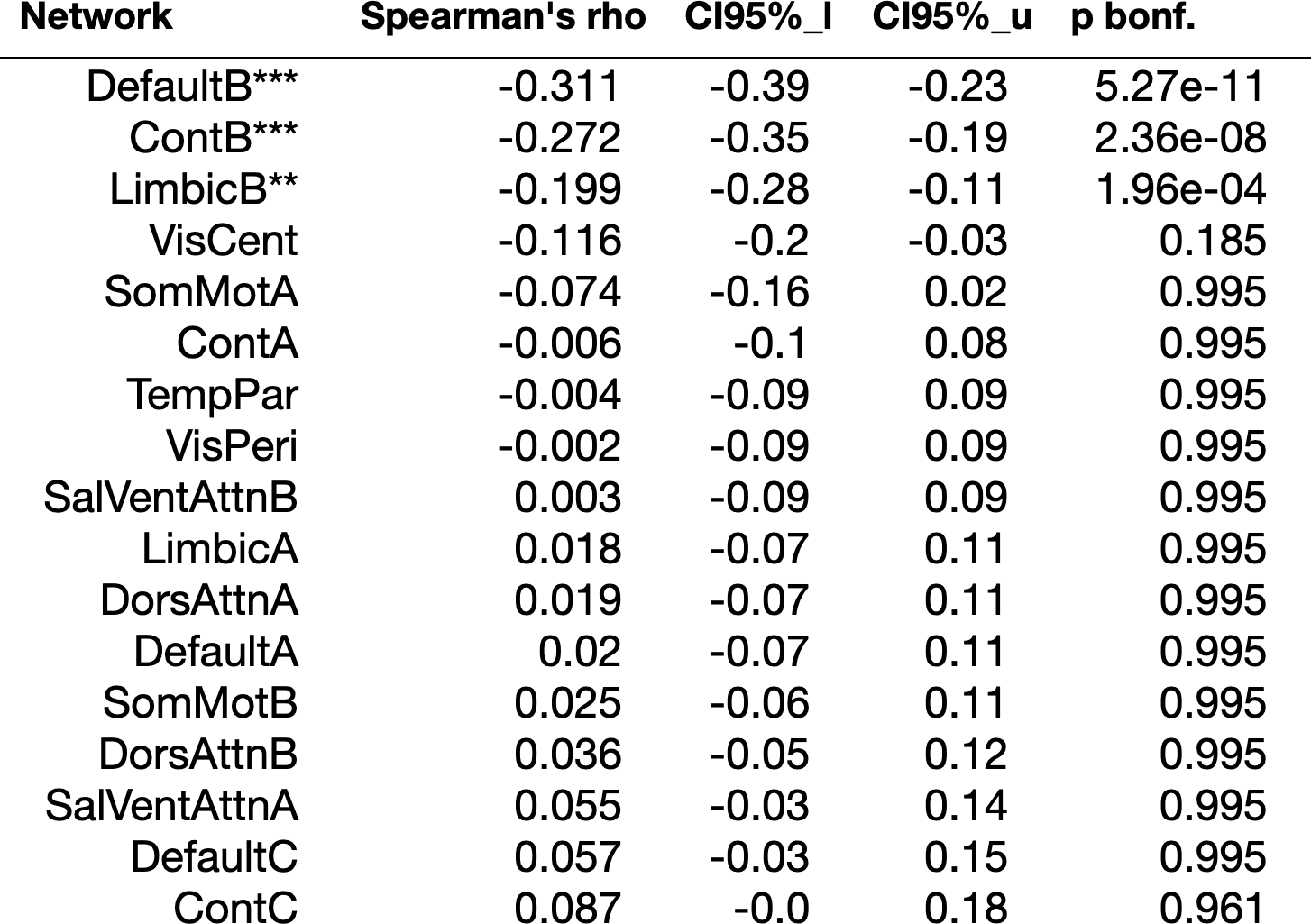
**

**Table S12. Mean network average controllability associated with age, with global network threshold of 0.015 the maximum streamline count per subject.** The mean network average controllability of the default mode (DefaultB), frontoparietal control (ContB), and Limbic (LimbicB) networks was negative associated with age. Education was included as a covariate for all associations. Networks are sorted in ascending order by the calculated Spearman’s ρ’s. The Bonferroni method was used to correct for multiple comparisons. *corrected p bonf. < 0.05, **p bonf*.*  < 0.001, ***p bonf. < 1e-05.

**
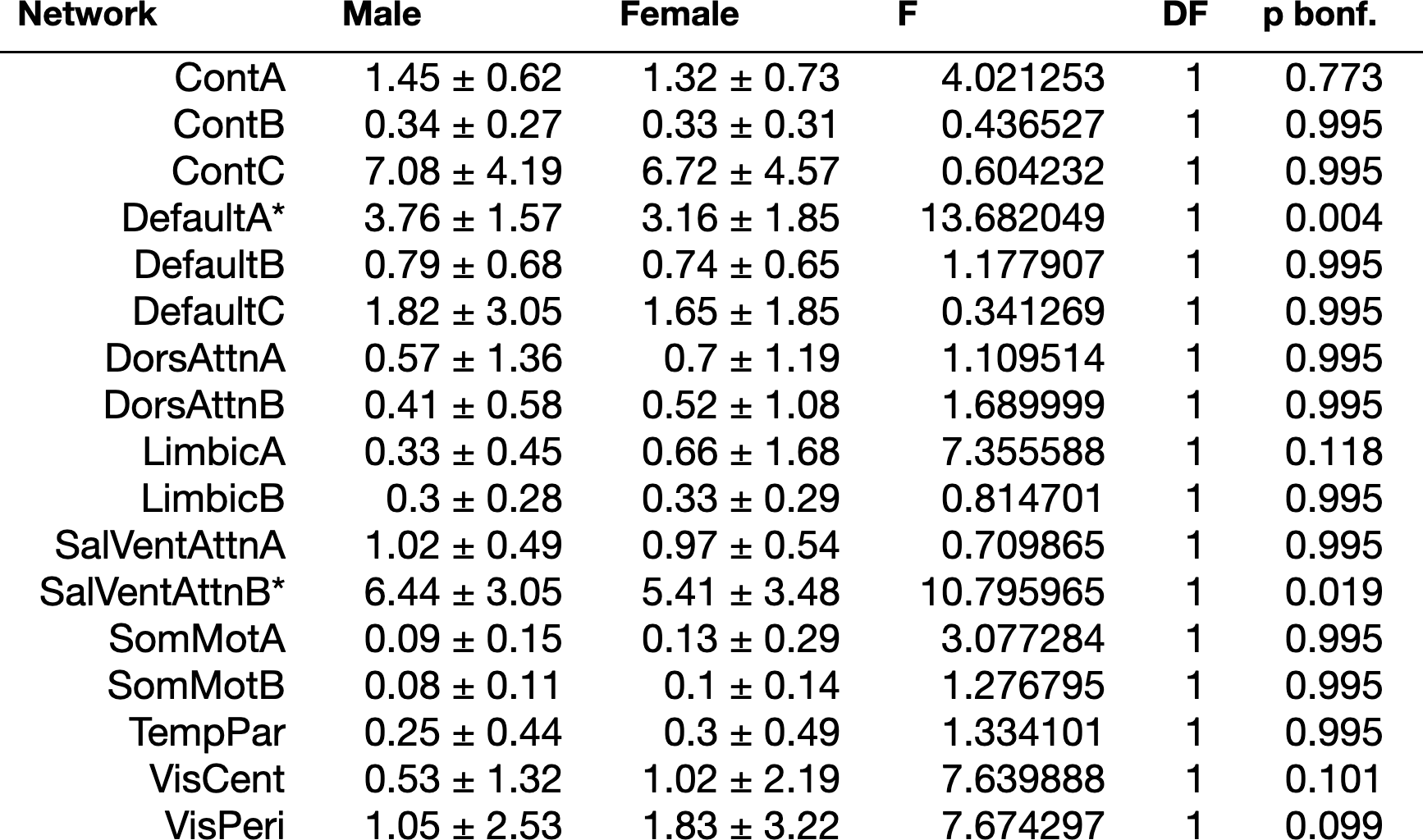
**

**Table S13**. **Sex-differences in mean network average controllability, with global network thresholds of 0.001 the maximum streamline count per subject.** For each network, the means in the average controllability for males and females are indicated, as well as their standard deviations (µ ± σ). Males had greater mean network average controllability of the salience/ventral attention (SalVentAttnB) and default mode (DefaultA) than females. Education and participant age were included as a covariate for all comparisons. The Bonferroni method was used to correct for multiple comparisons. *corrected p bonf. < 0.05, **p bonf*.*  < 0.001, ***p bonf. < 1e-05.

**
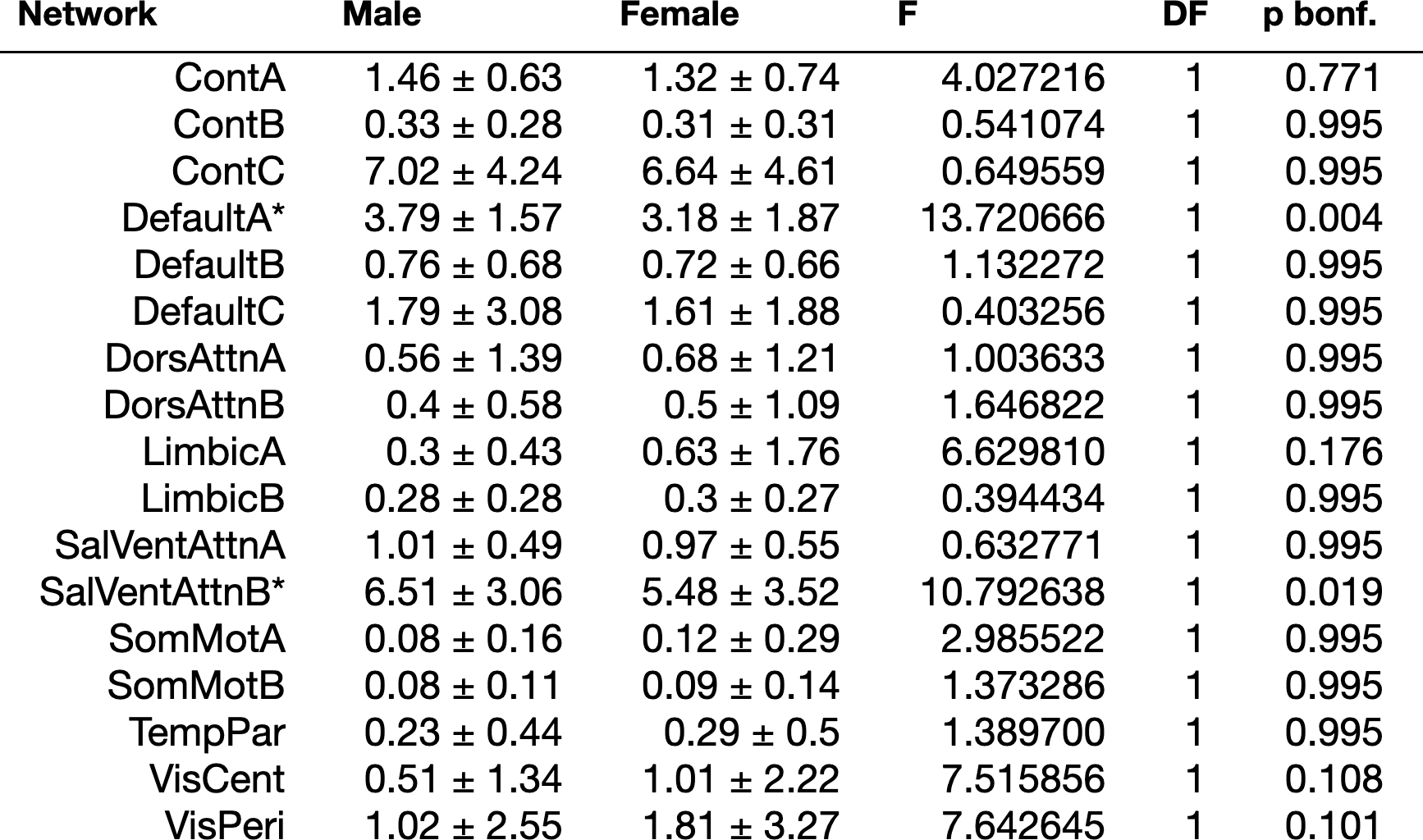
**

**Table S14**. **Sex-differences in mean network average controllability, with global network thresholds of 0.005 the maximum streamline count per subject.** For each network, the means in the average controllability for males and females are indicated, as well as their standard deviations (µ ± σ). Males had greater mean network average controllability of the salience/ventral attention (SalVentAttnB) and default mode (DefaultA) than females. Education and participant age were included as a covariate for all comparisons. The Bonferroni method was used to correct for multiple comparisons. *corrected p bonf. < 0.05, **p bonf*.*  < 0.001, ***p bonf. < 1e-05.


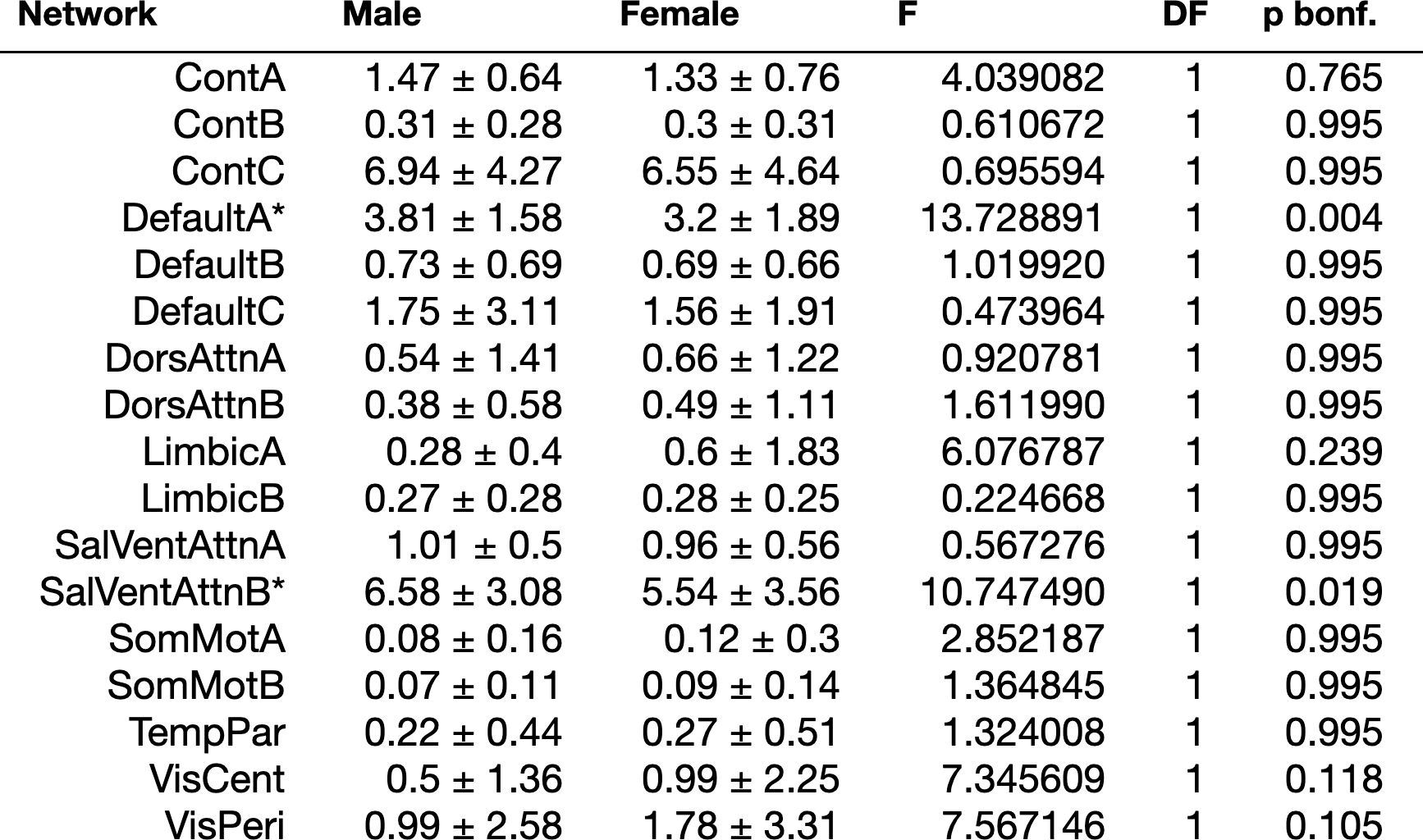


**Table S15**. **Sex-differences in mean network average controllability, with global network thresholds of 0.010 the maximum streamline count per subject.** For each network, the means in the average controllability for males and females are indicated, as well as their standard deviations (µ ± σ). Males had greater mean network average controllability of the salience/ventral attention (SalVentAttnB) and default mode (DefaultA) than females. Education and participant age were included as a covariate for all comparisons. The Bonferroni method was used to correct for multiple comparisons. *corrected p bonf. < 0.05, **p bonf*.*  < 0.001, ***p bonf. < 1e-05.


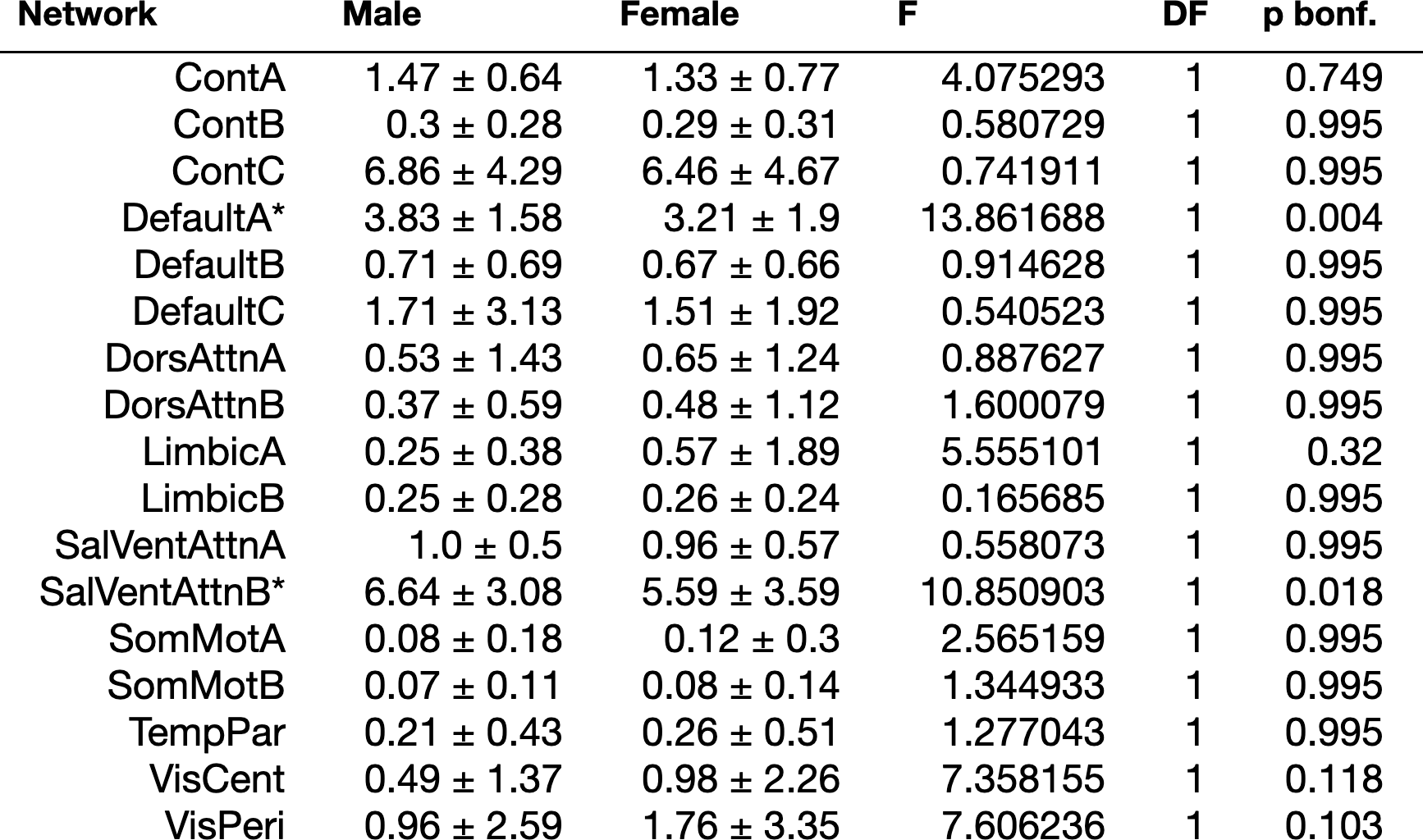


**Table S16**. **Sex-differences in mean network average controllability, with global network thresholds of 0.015 the maximum streamline count per subject.** For each network, the means in the average controllability for males and females are indicated, as well as their standard deviations (µ ± σ). Males had greater mean network average controllability of the salience/ventral attention (SalVentAttnB) and default mode (DefaultA) than females. Education and participant age were included as a covariate for all comparisons. The Bonferroni method was used to correct for multiple comparisons. *corrected p bonf. < 0.05, **p bonf*.*  < 0.001, ***p bonf. < 1e-05.


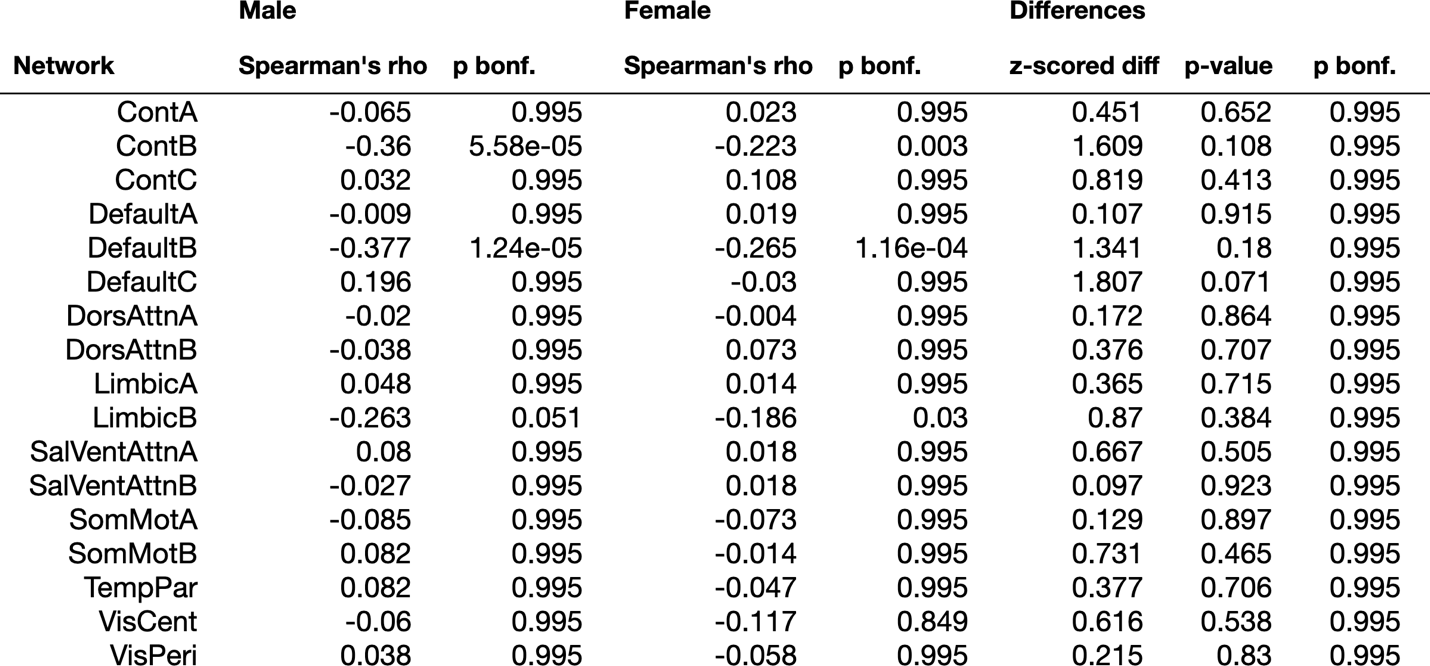


**Table S17**. **Sex-differences in the relationships between mean network average controllability and age, with global network thresholds of 0.001 the maximum streamline count per subject.** Rank correlations between age and mean network average controllability for each of the 17 networks were computed. Then we computed the z-scored difference of the z-transformed rank correlations, and its’ significance. The raw p-value of the z-scored difference is reported, as well as the p-value after correction (p bonf.) using the Bonferroni method to correct for multiple comparisons. We did not observe any significantly different correlations between sexes for any of the 17 networks assessed.


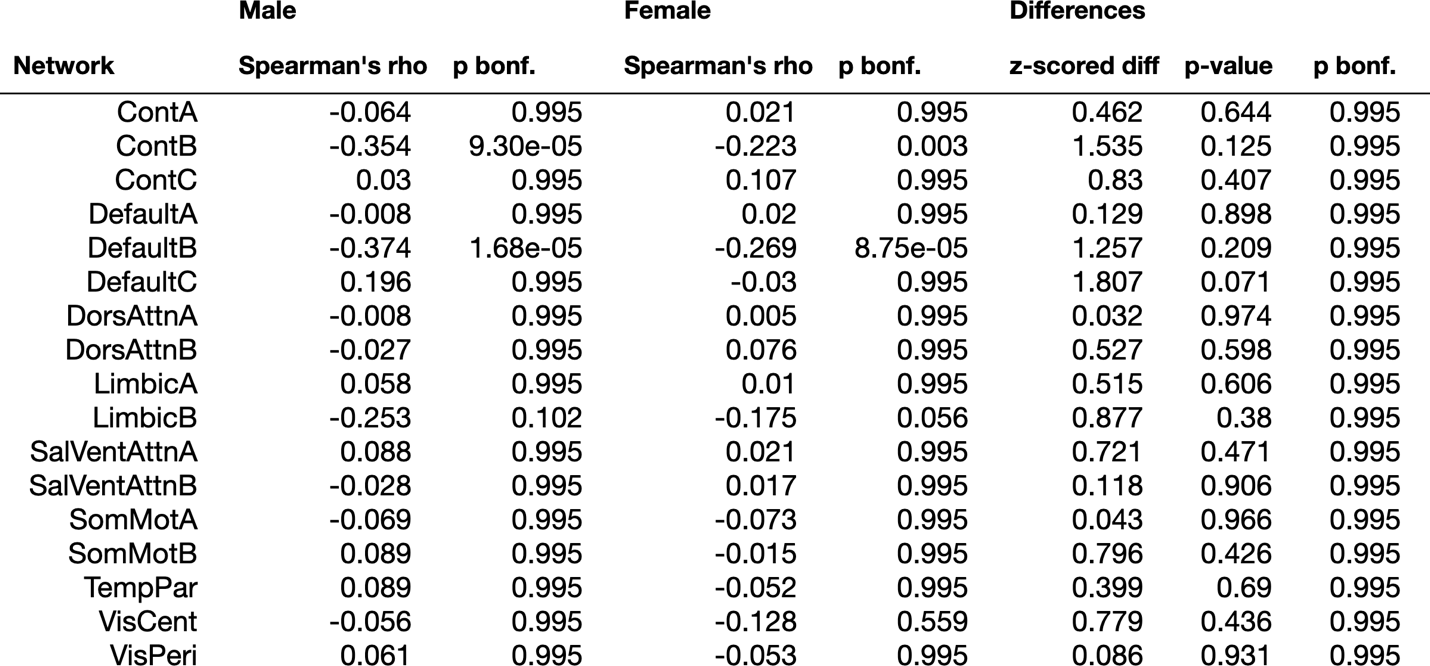


**Table S18**. **Sex-differences in the relationships between mean network average controllability and age, with global network thresholds of 0.005 the maximum streamline count per subject.** Rank correlations between age and mean network average controllability for each of the 17 networks were computed. Then we computed the z-scored difference of the z-transformed rank correlations, and its’ significance. The raw p-value of the z-scored difference is reported, as well as the p-value after correction (p bonf.) using the Bonferroni method to correct for multiple comparisons. We did not observe any significantly different correlations between sexes for any of the 17 networks assessed.


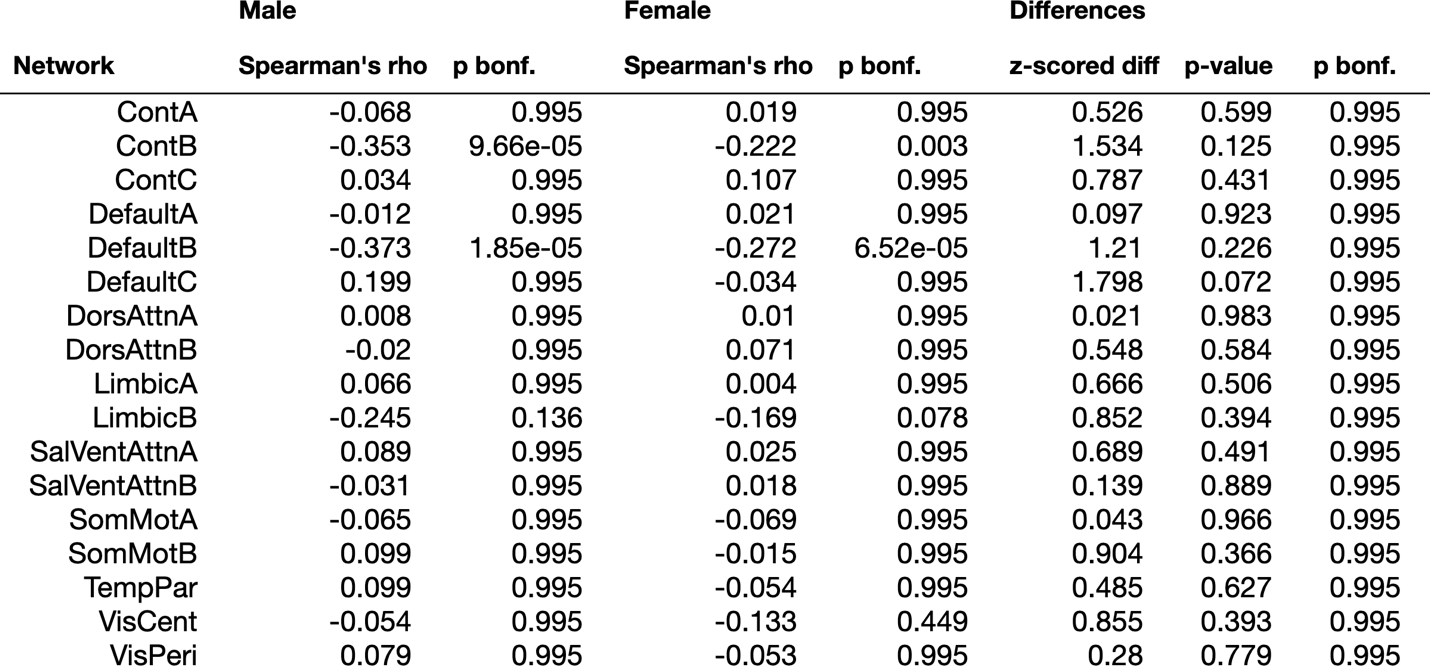


**Table S19**. **Sex-differences in the relationships between mean network average controllability and age, with global network thresholds of 0.010 the maximum streamline count per subject.** Rank correlations between age and mean network average controllability for each of the 17 networks were computed. Then we computed the z-scored difference of the z-transformed rank correlations, and its’ significance. The raw p-value of the z-scored difference is reported, as well as the p-value after correction (p bonf.) using the Bonferroni method to correct for multiple comparisons. We did not observe any significantly different correlations between sexes for any of the 17 networks assessed.


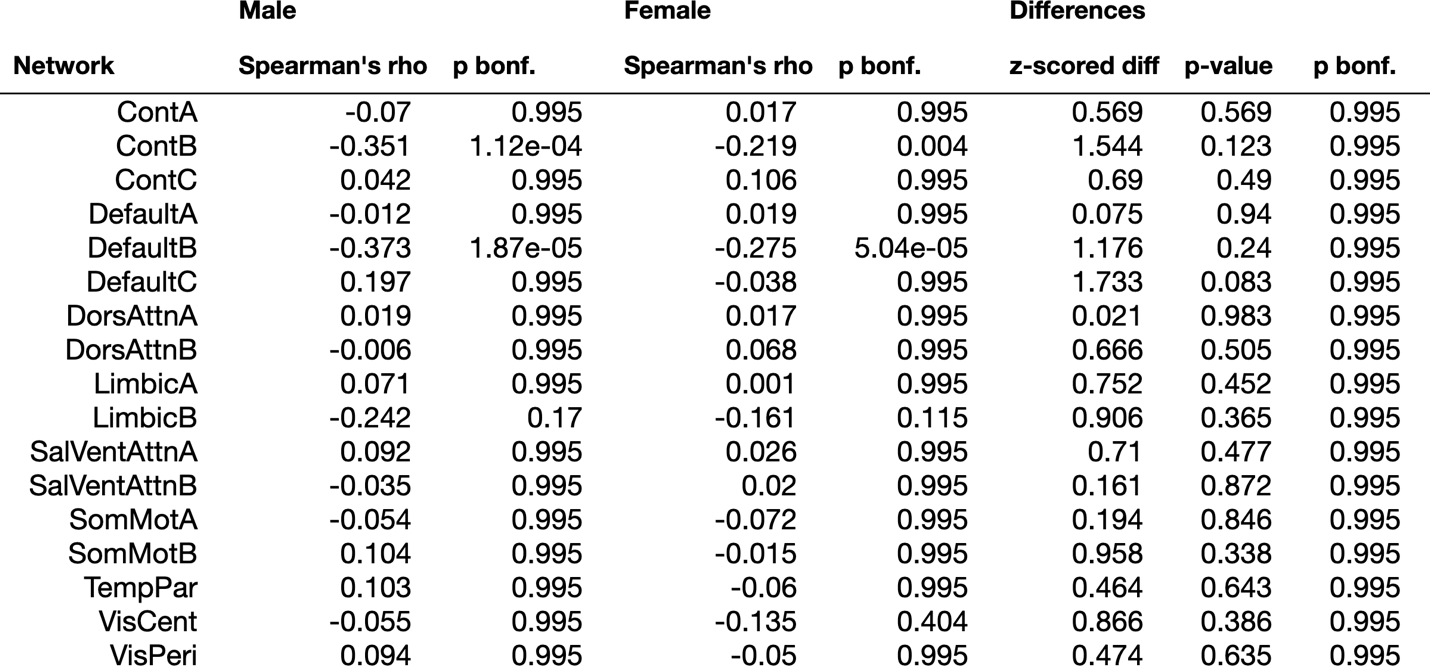


**Table S20**. **Sex-differences in the relationships between mean network average controllability and age, with global network thresholds of 0.015 the maximum streamline count per subject.** Rank correlations between age and mean network average controllability for each of the 17 networks were computed. Then we computed the z-scored difference of the z-transformed rank correlations, and its’ significance. The raw p-value of the z-scored difference is reported, as well as the p-value after correction (p bonf.) using the Bonferroni method to correct for multiple comparisons. We did not observe any significantly different correlations between sexes for any of the 17 networks assessed.

**
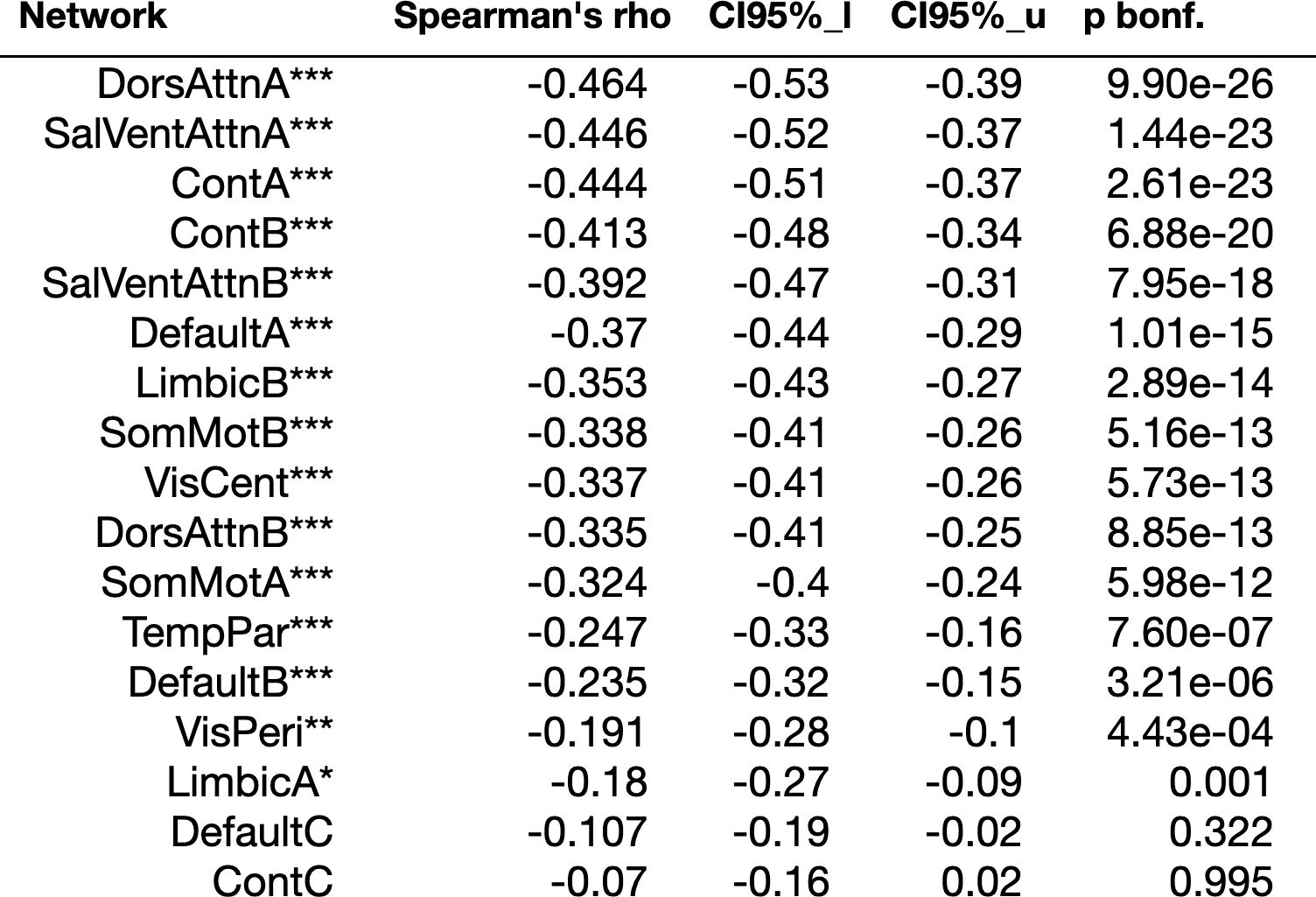
**

**Table S21. Average degree for each network associated with age, with global network threshold of 0.001 the maximum streamline count per subject.** For 15 of the 17 networks, average network degree was negatively associated with age. Education was included as a covariate for all associations. Networks are sorted in ascending order by the calculated Spearman’s ρ’s. The Bonferroni method was used to correct for multiple comparisons. *corrected p bonf. < 0.05, **p bonf*.*  < 0.001, ***p bonf. < 1e-05.


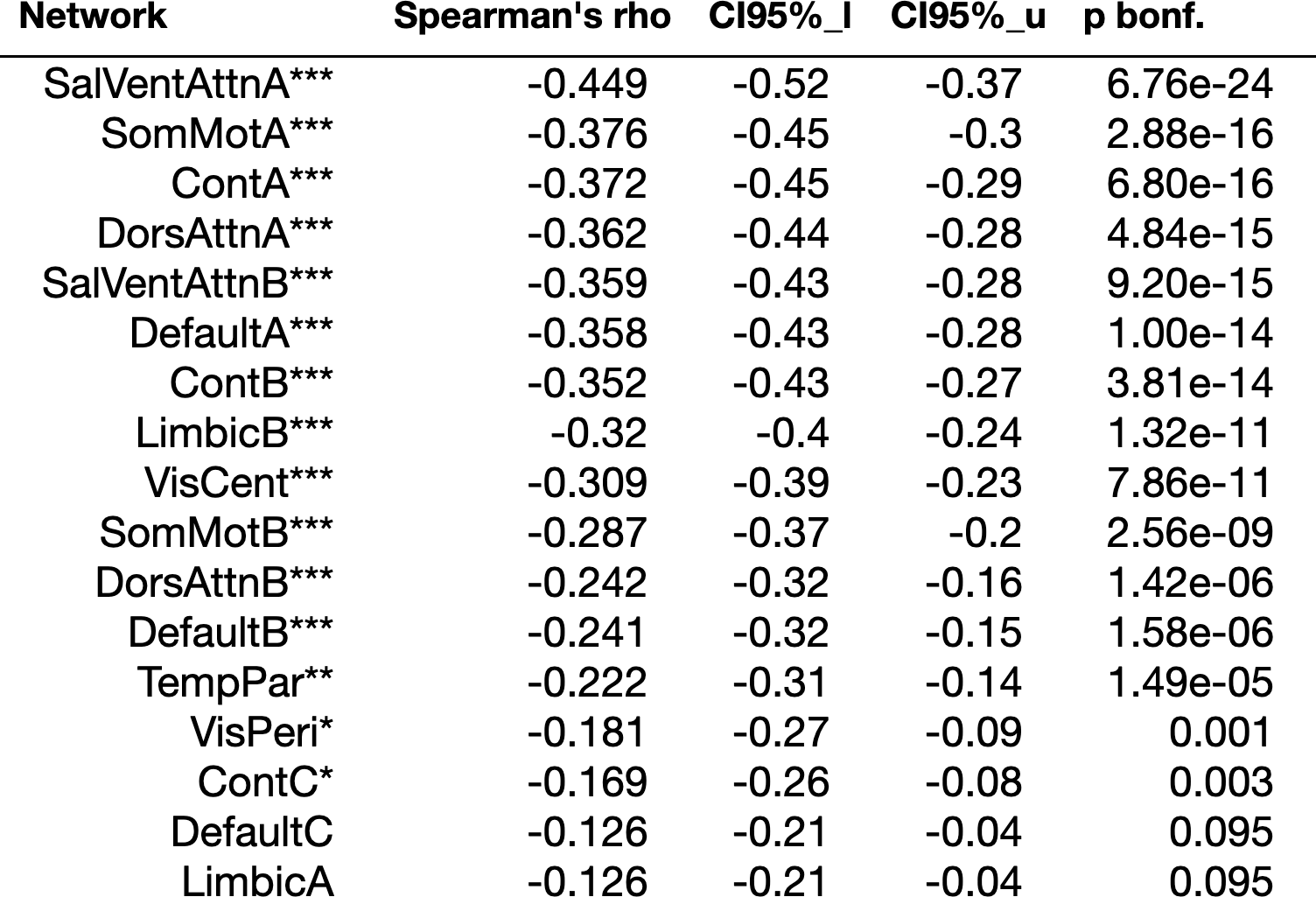


**Table S22. Average degree for each network associated with age, with global network threshold of 0.005 the maximum streamline count per subject.** For 15 of the 17 networks, average network degree was negatively associated with age. Education was included as a covariate for all associations. Networks are sorted in ascending order by the calculated Spearman’s ρ’s. The Bonferroni method was used to correct for multiple comparisons. *corrected p bonf. < 0.05, **p bonf*.*  < 0.001, ***p bonf. < 1e-05.


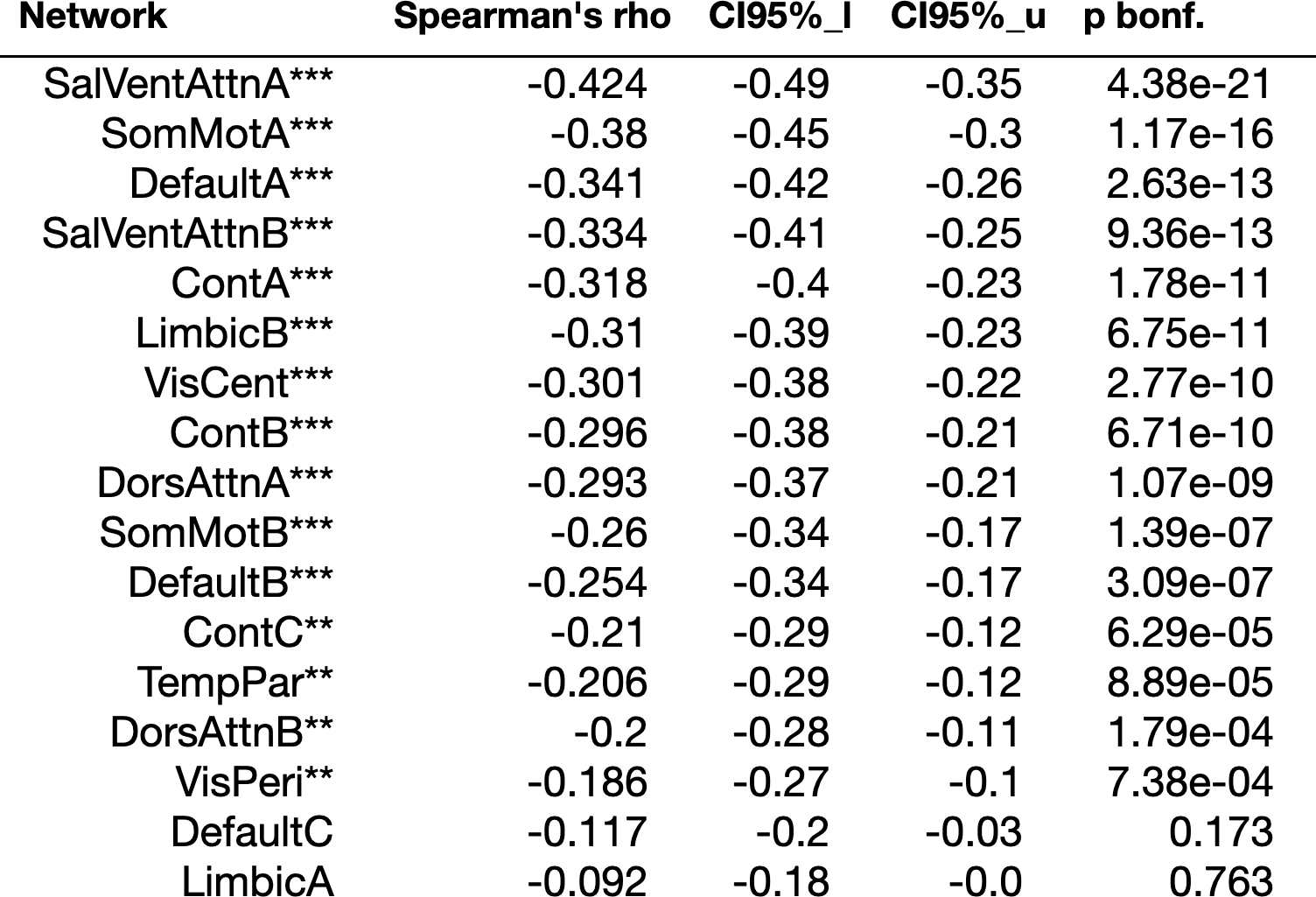


**Table S23. Average degree for each network associated with age, with global network threshold of 0.010 the maximum streamline count per subject.** For 15 of the 17 networks, average network degree was negatively associated with age. Networks are sorted in ascending order by the calculated Spearman’s ρ’s. Education was included as a covariate for all associations. The Bonferroni method was used to correct for multiple comparisons. *corrected p bonf. < 0.05, **p bonf*.*  < 0.001, ***p bonf. < 1e-05.


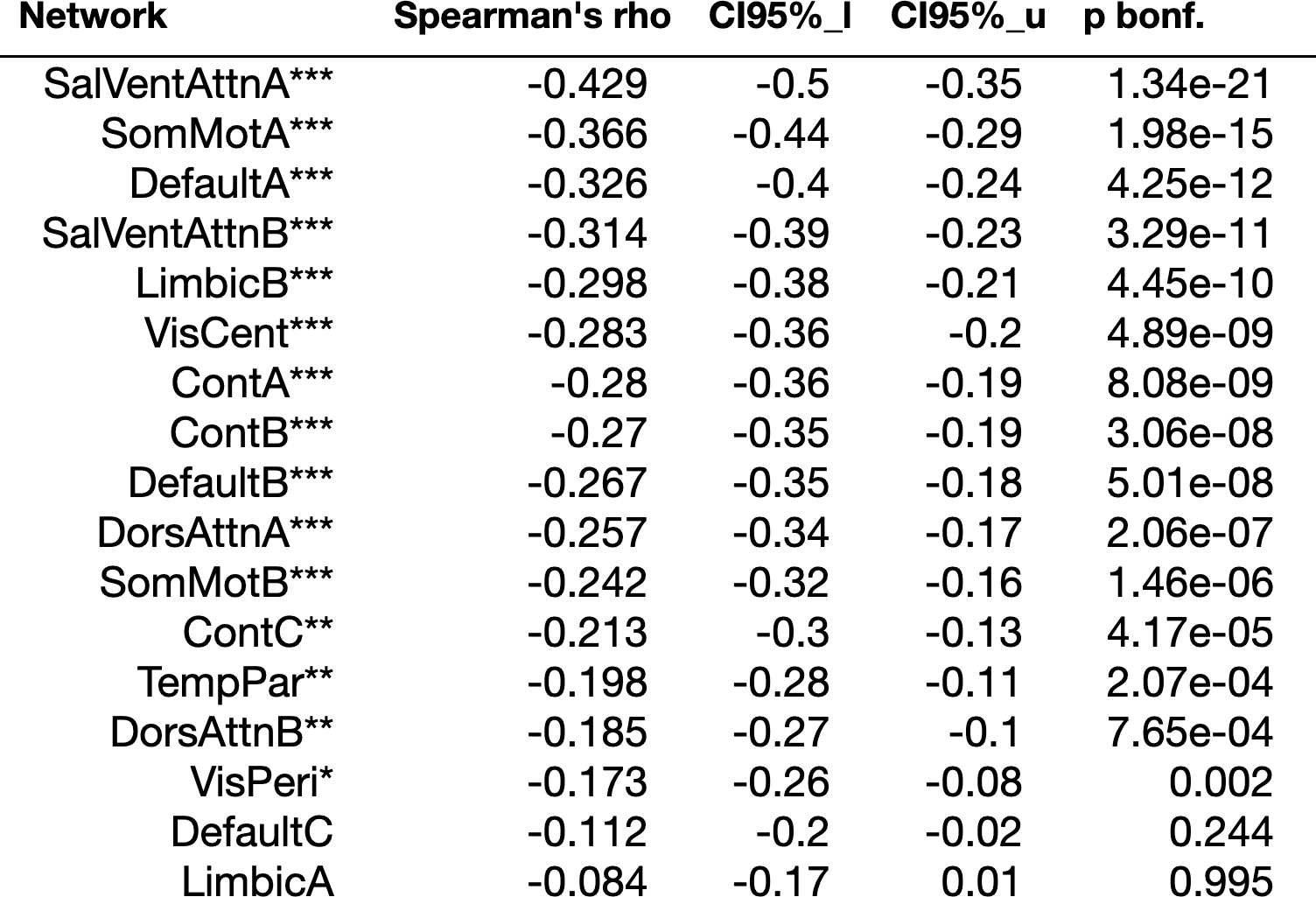


**Table S24. Average degree for each network associated with age, with global network threshold of 0.015 the maximum streamline count per subject.** For 15 of the 17 networks, average network degree was negatively associated with age. Education was included as a covariate for all associations. Networks are sorted in ascending order by the calculated Spearman’s ρ’s. The Bonferroni method was used to correct for multiple comparisons. *corrected p bonf. < 0.05, **p bonf*.*  < 0.001, ***p bonf. < 1e-05.

**
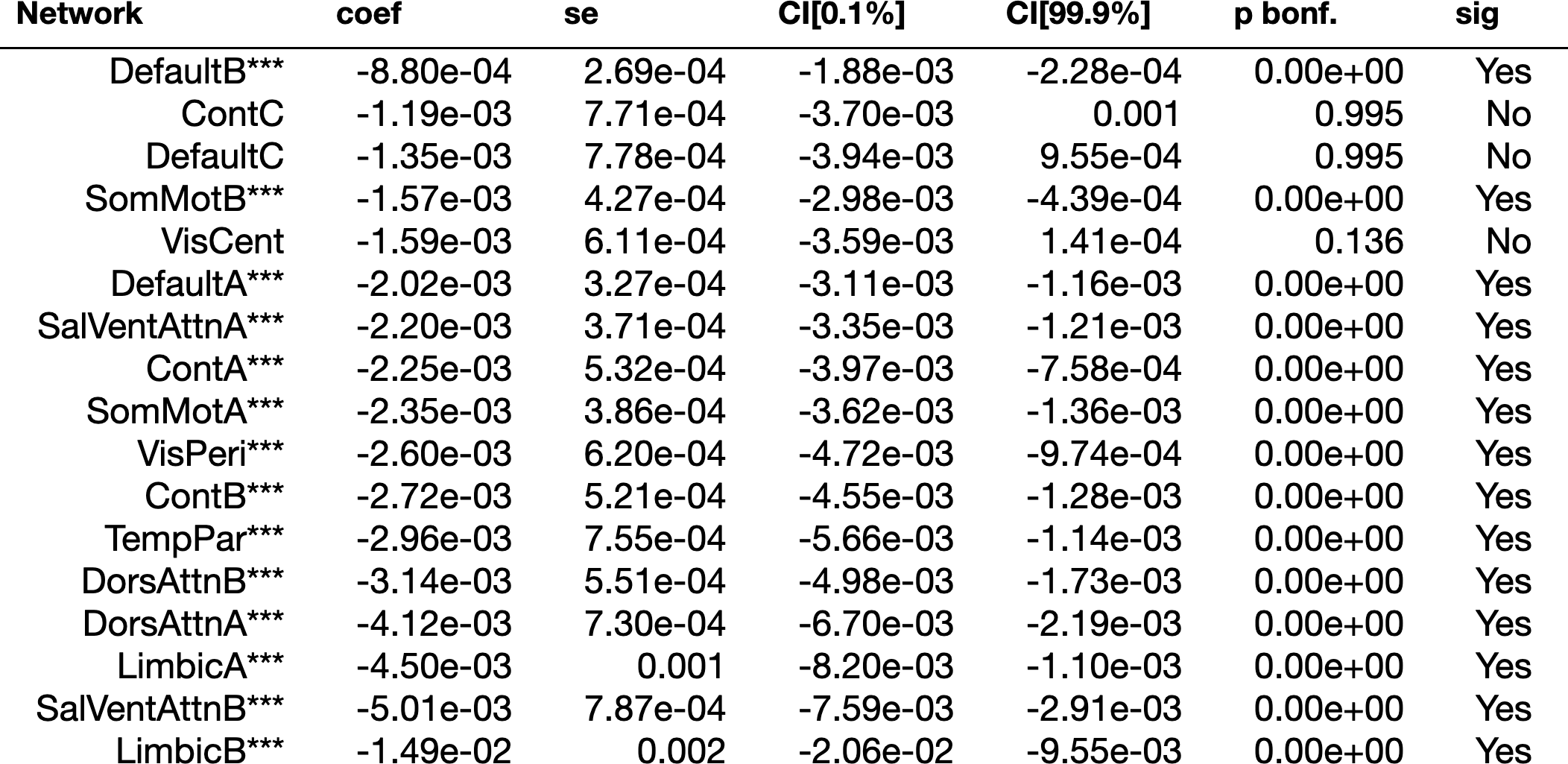
**

**Table S25. Statistics for the indirect mediation by degree on the relationship between age and average controllability for each of the 17 networks, with a global threshold of 0.001 the maximum streamline count per subject.** For all 14 of 17 networks there was a significant mediation by degree (*p_bonf_*_._ < 0.05). Education was included as a covariate for all mediations. Significance was determined if the confidence intervals for each coefficient did not cross zero after setting the α = 0.05/17 to correct for multiple comparisons. *corrected p bonf. < 0.05, **p bonf*.*  < 0.001, ***p bonf. < 1e-05.

**
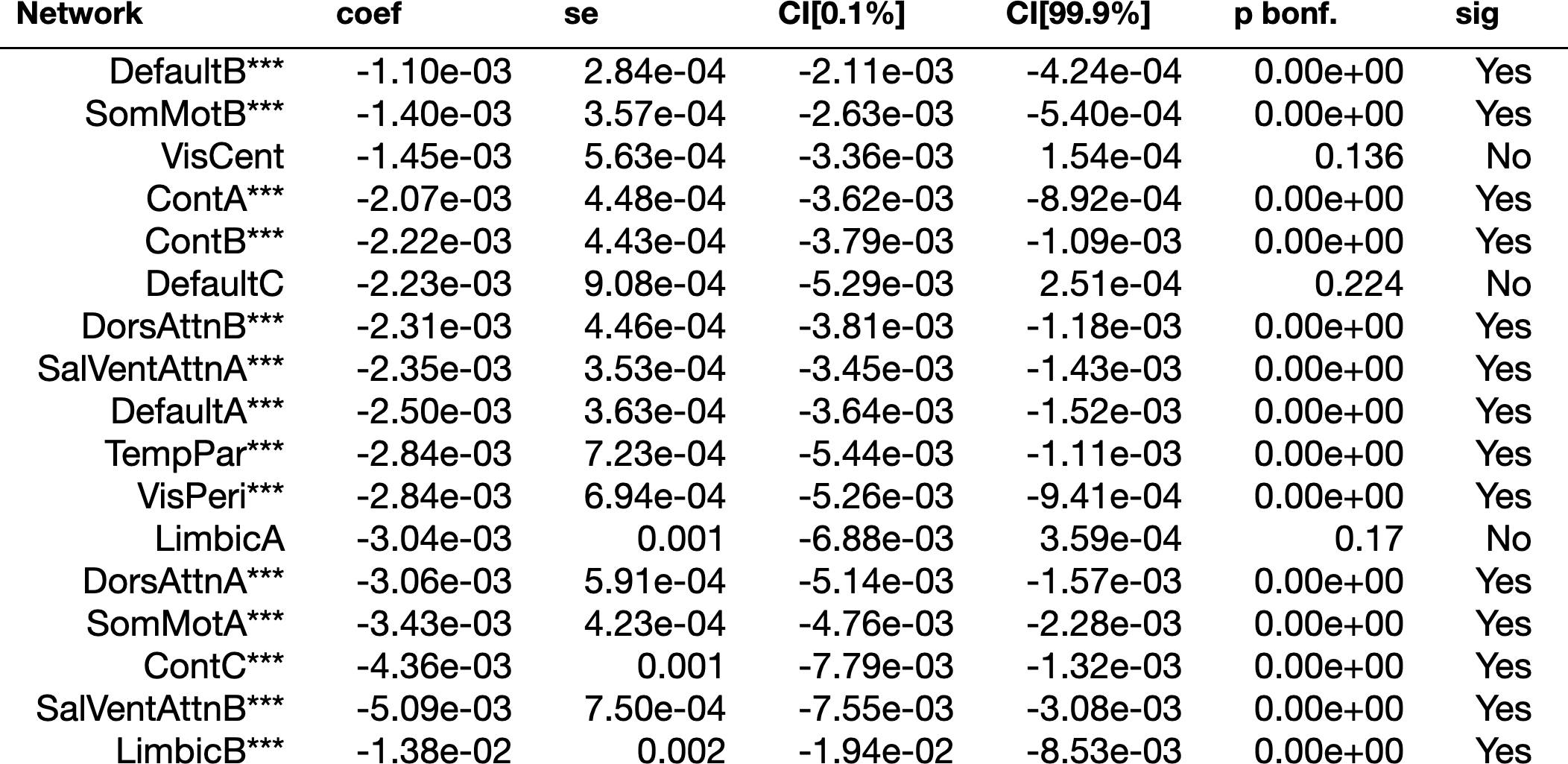
**

**Table S26. Statistics for the indirect mediation by degree on the relationship between age and average controllability for each of the 17 networks, with a global threshold of 0.005 the maximum streamline count per subject.** For 14 of 17 networks there was a significant mediation by degree (*p_bonf_*_._ < 0.05). Education was included as a covariate for all mediations. Significance was determined if the confidence intervals for each coefficient did not cross zero after setting the α = 0.05/17 to correct for multiple comparisons. *corrected p bonf. < 0.05, **p bonf*.*  < 0.001, ***p bonf. < 1e-05.

**
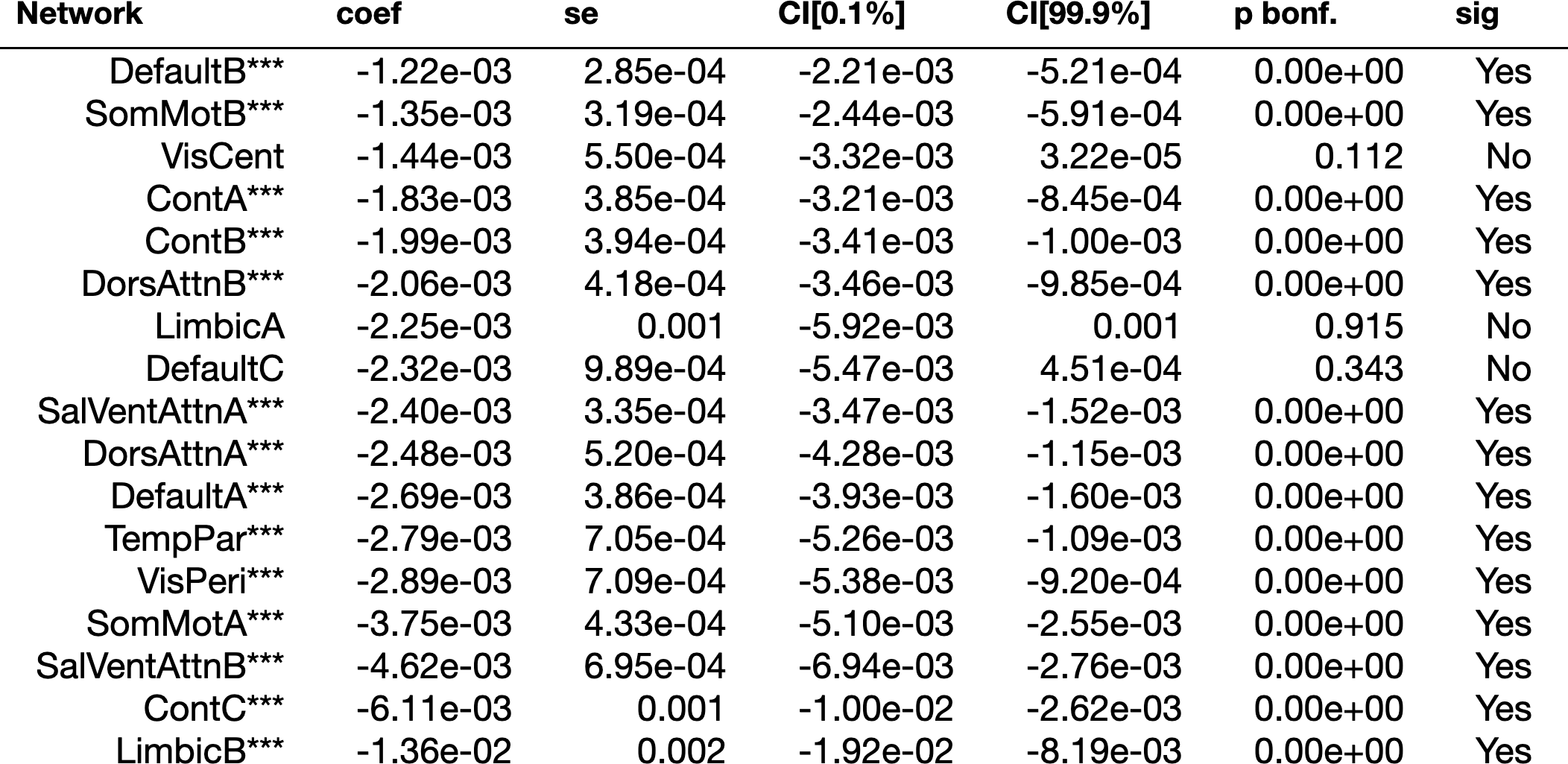
**

**Table S27. Statistics for the indirect mediation by degree on the relationship between age and average controllability for each of the 17 networks, with a global threshold of 0.010 the maximum streamline count per subject.** For all but 14 of 17 networks there was a significant mediation by degree (*p* < 0.05). Education was included as a covariate for all mediations. Significance was determined if the confidence intervals for each coefficient did not cross zero after setting the α = 0.05/17 to correct for multiple comparisons. *corrected p bonf. < 0.05, **p bonf*.*  < 0.001, ***p bonf. < 1e-05.

**
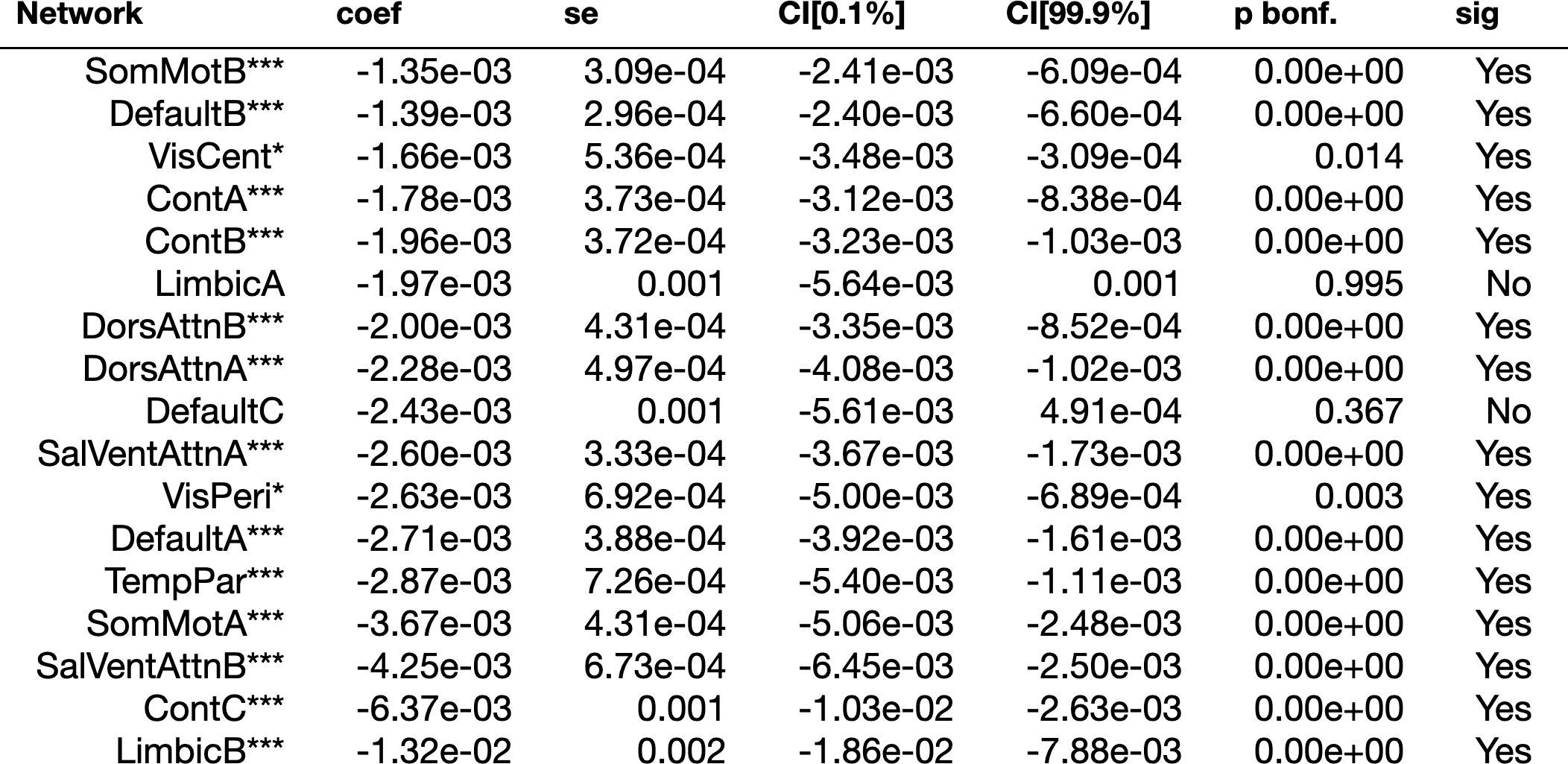
**

**Table S28. Statistics for the indirect mediation by degree on the relationship between age and average controllability for each of the 17 networks, with a global threshold of 0.015 the maximum streamline count per subject.** For 15 of 17 networks there was a significant mediation by degree (*p_bonf_*_._ < 0.05). Education was included as a covariate for all mediations. Significance was determined if the confidence intervals for each coefficient did not cross zero after setting the α = 0.05/17 to correct for multiple comparisons. *corrected p bonf. < 0.05, **p bonf*.*  < 0.001, ***p bonf. < 1e-05.

**
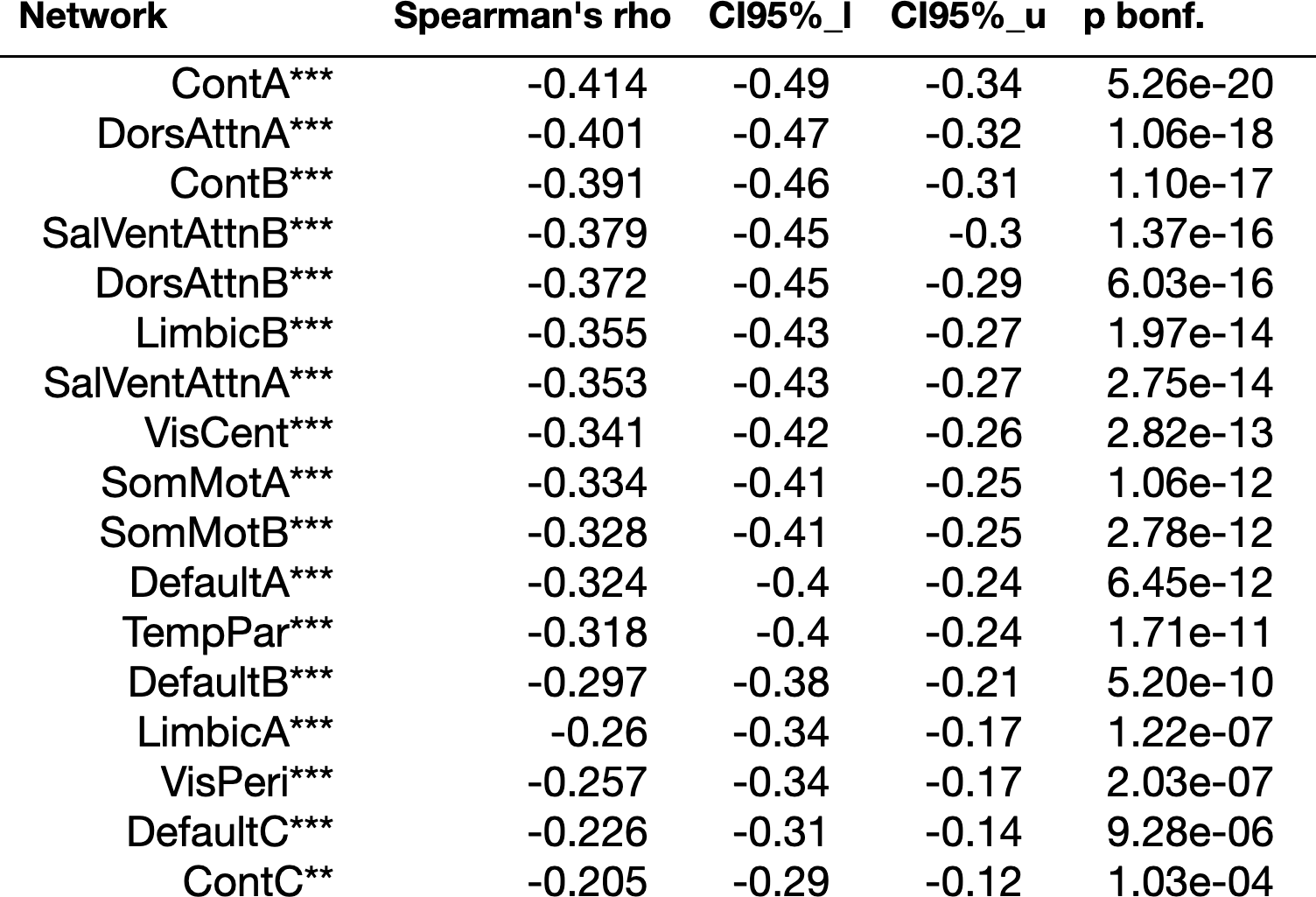
**

**Table S29. The statistics and confidence intervals for rank correlations of average network redundancy with age, with a global network threshold of 0.001 the maximum streamline count per subject.** Average redundancy of all networks showed a negative relationship with age. Education was included as a covariate for all associations. Networks are sorted in ascending order by the calculated Spearman’s ρ’s. The Bonferroni method was used to correct for multiple comparisons. *corrected p bonf. < 0.05, **p bonf*.*  < 0.001, ***p bonf. < 1e-05.


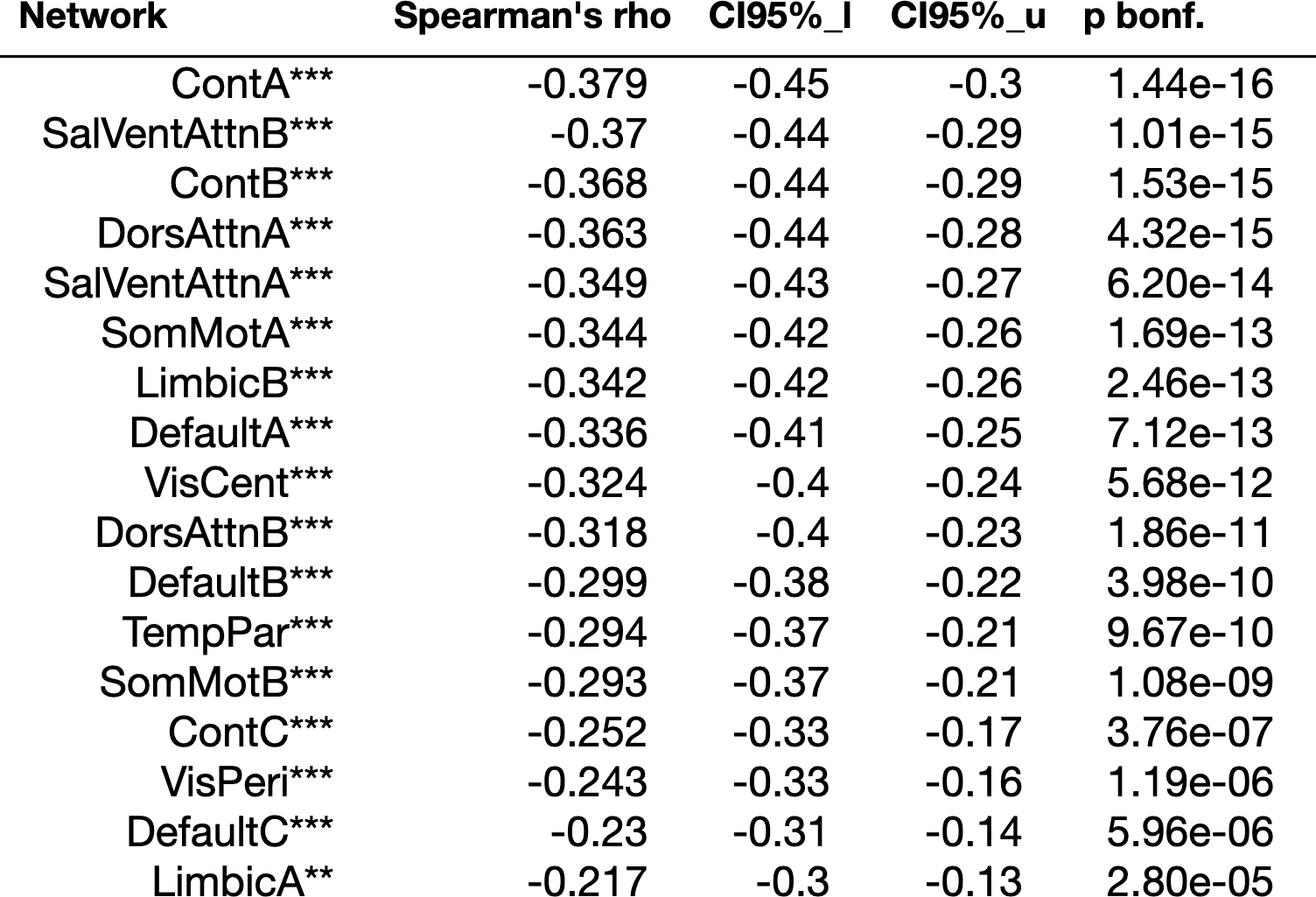


**Table S30. The statistics and confidence intervals for rank correlations of average network redundancy with age, with a global network threshold of 0.005 the maximum streamline count per subject.** Average redundancy of all networks showed a negative relationship with age. Education was included as a covariate for all associations. Networks are sorted in ascending order by the calculated Spearman’s ρ’s. The Bonferroni method was used to correct for multiple comparisons. *corrected p bonf. < 0.05, **p bonf*.*  < 0.001, ***p bonf. < 1e-05.

**
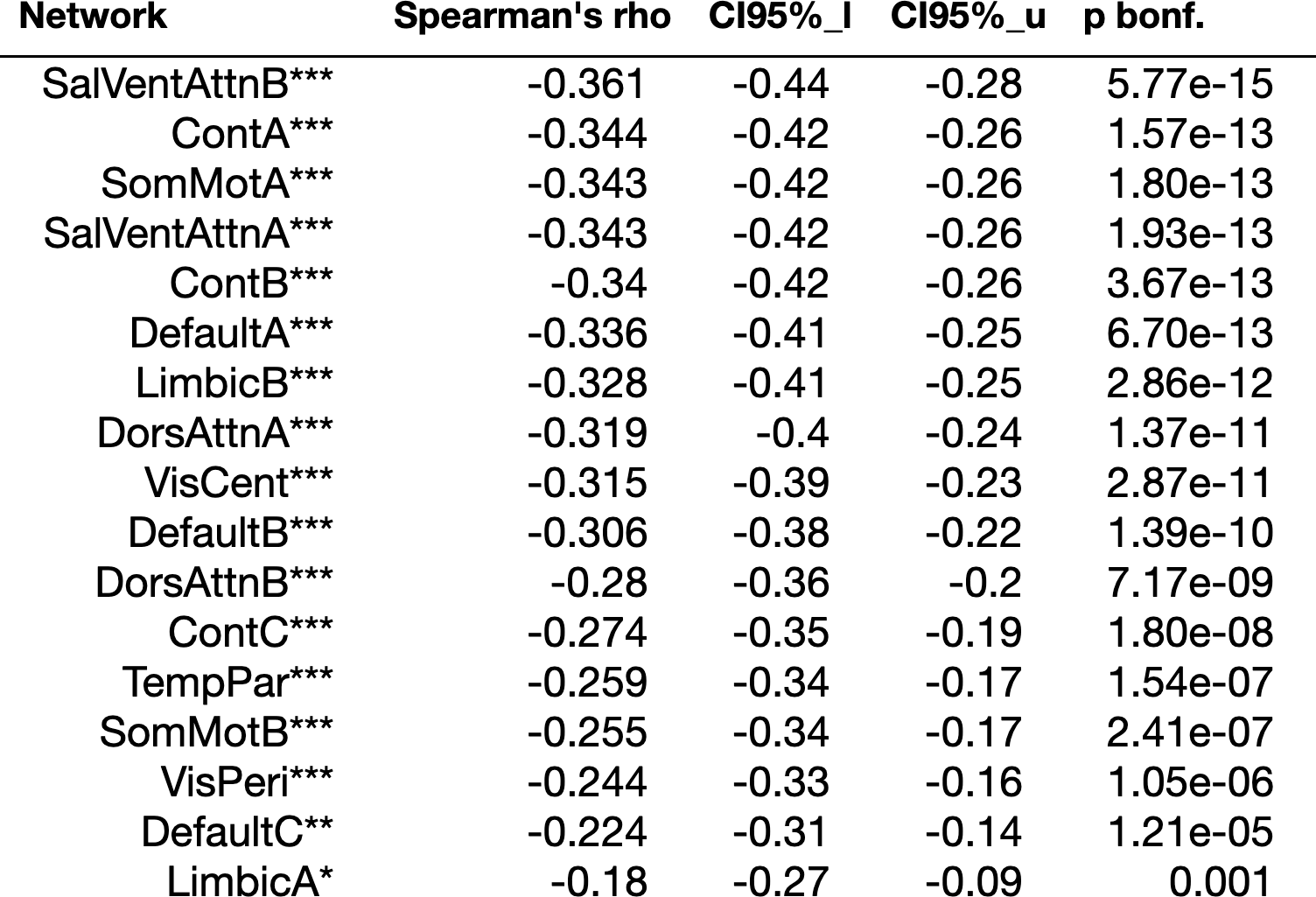
**

**Table S31. The statistics and confidence intervals for rank correlations of average network redundancy with age, with a global network threshold of 0.010 the maximum streamline count per subject.**\Average redundancy of all networks showed a negative relationship with age. Education was included as a covariate for all associations. Networks are sorted in ascending order by the calculated Spearman’s ρ’s. The Bonferroni method was used to correct for multiple comparisons. *corrected p bonf. < 0.05, **p bonf*.*  < 0.001, ***p bonf. < 1e-05.


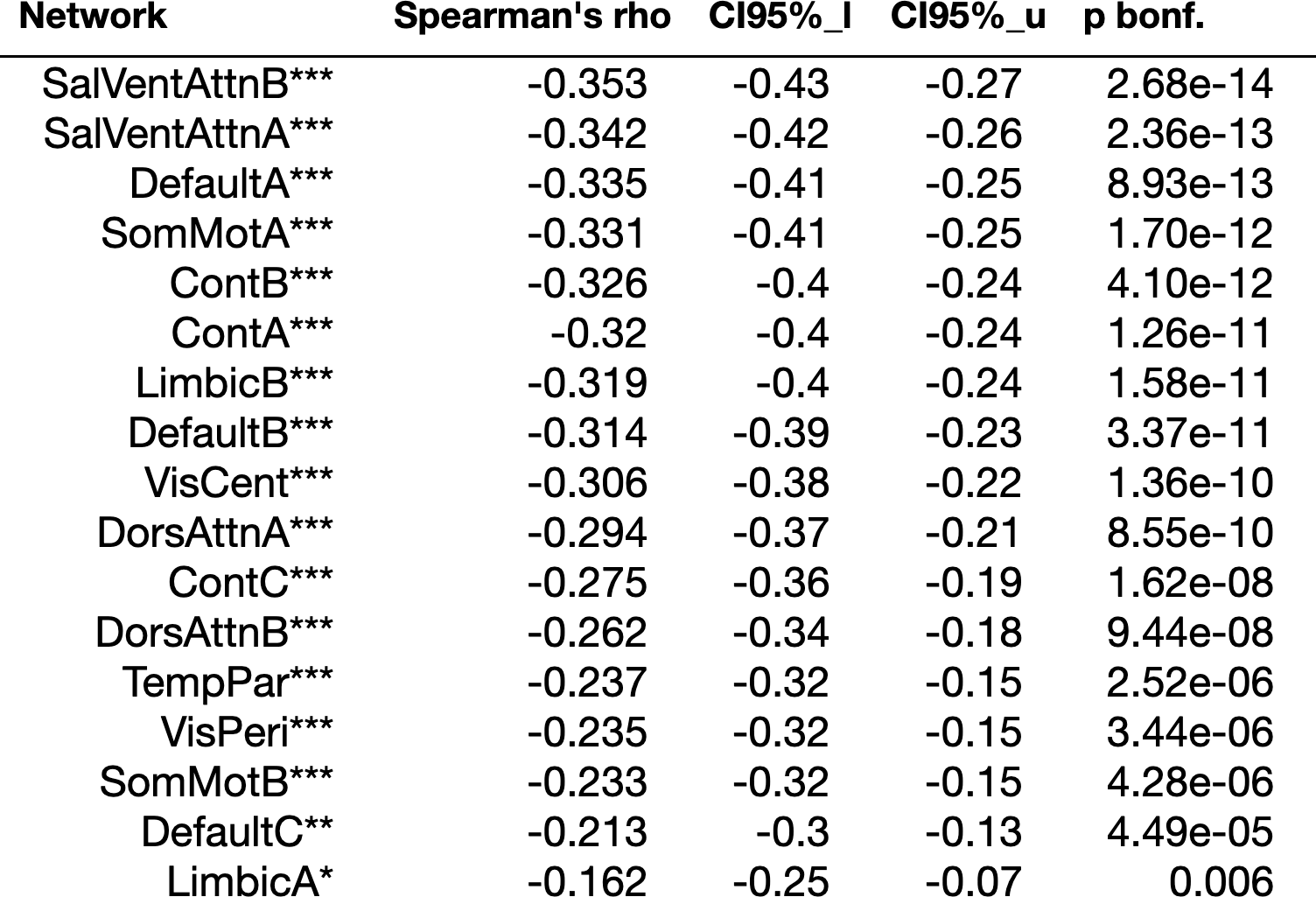


**Table S32. The statistics and confidence intervals for rank correlations of average network redundancy with age, with a global network threshold of 0.015 the maximum streamline count per subject.** Average redundancy of all networks showed a negative relationship with age. Education was included as a covariate for all associations. Networks are sorted in ascending order by the calculated Spearman’s ρ’s. The Bonferroni method was used to correct for multiple comparisons. *corrected p bonf. < 0.05, **p bonf*.*  < 0.001, ***p bonf. < 1e-05.

**
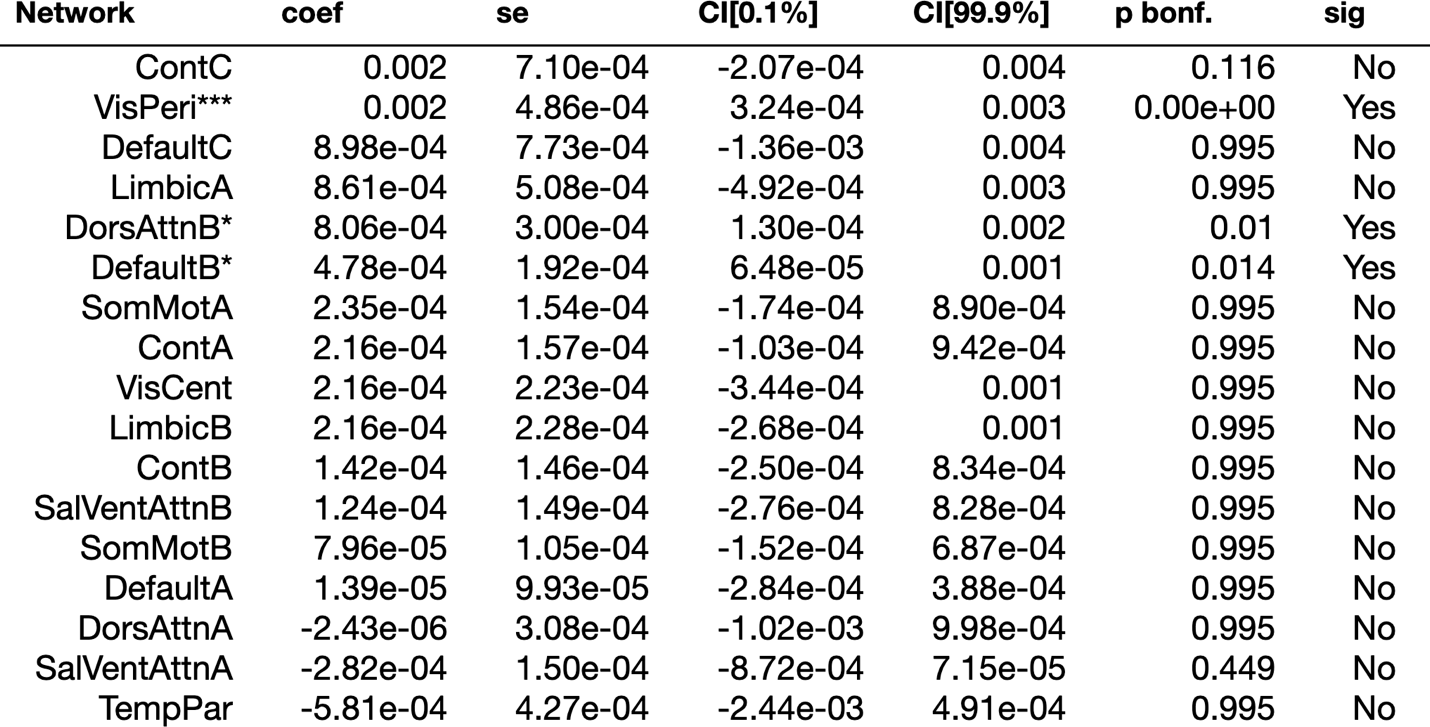
**

**Table S33. Statistics for the indirect mediation by redundancy with degree as a covariate on the relationship between age and average controllability for each of the 17 networks, with a global threshold of 0.001.** For 3 of 17 networks (VisPeri, DorsAttnB, DefaultB) there was a significant mediation by redundancy. Education was included as a covariate for all mediations. Significance was determined if the confidence intervals for each coefficient did not cross zero after setting the α = 0.05/17 to correct for multiple comparisons. *corrected p bonf. < 0.05, **p bonf*.*  < 0.001, ***p bonf. < 1e-05.

**
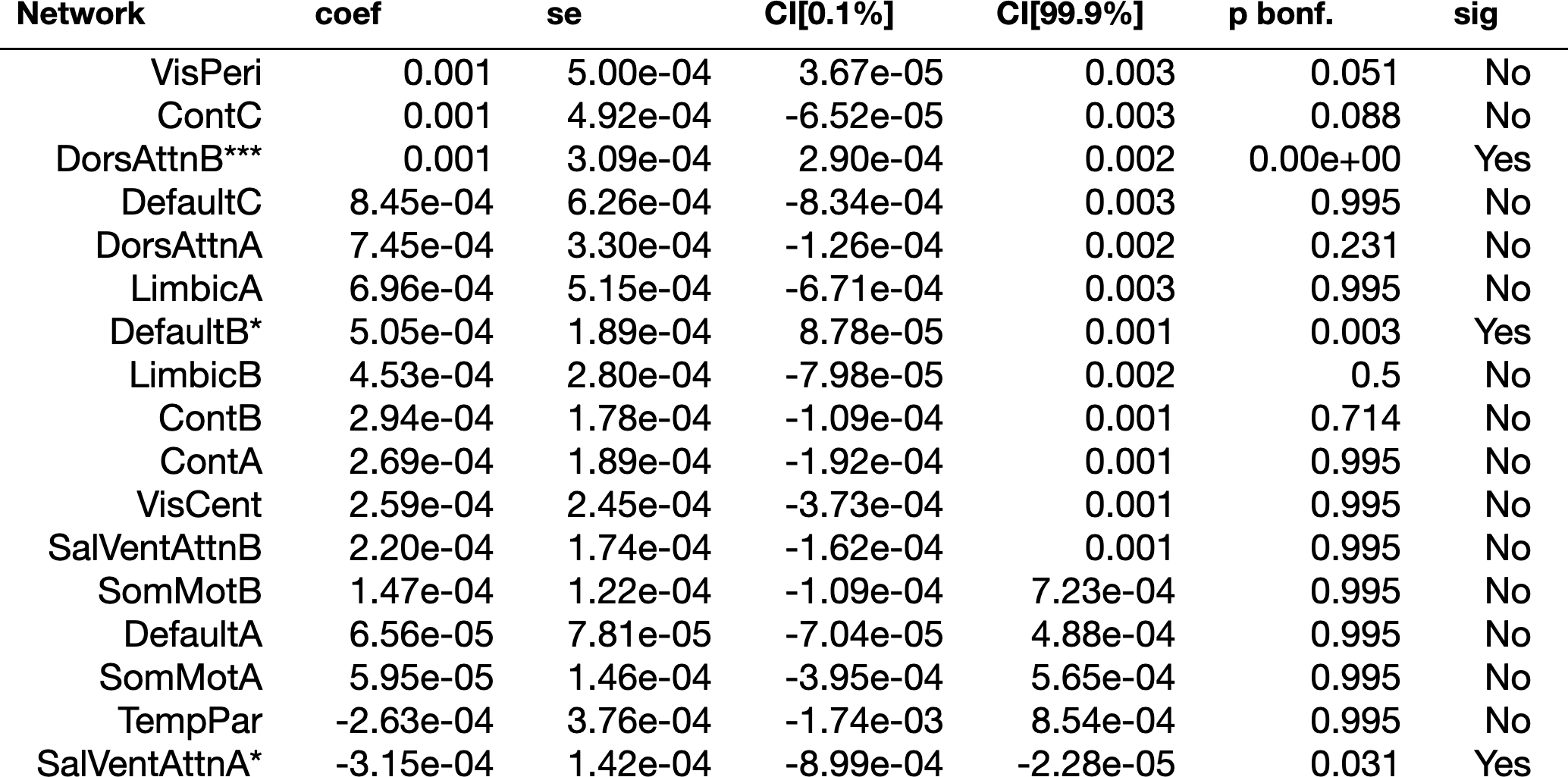
**

**Table S34. Statistics for the indirect mediation by redundancy with degree as a covariate on the relationship between age and average controllability for each of the 17 networks, with a global threshold of 0.005.** For 3 of 17 networks (DorsAttnB, DefaultB, SalVentAttnA) there was a significant mediation by redundancy. Education was included as a covariate for all mediations. Significance was determined if the confidence intervals for each coefficient did not cross zero after setting the α = 0.05/17 to correct for multiple comparisons. *corrected p bonf. < 0.05, **p bonf*.*  < 0.001, ***p bonf. < 1e-05.

**
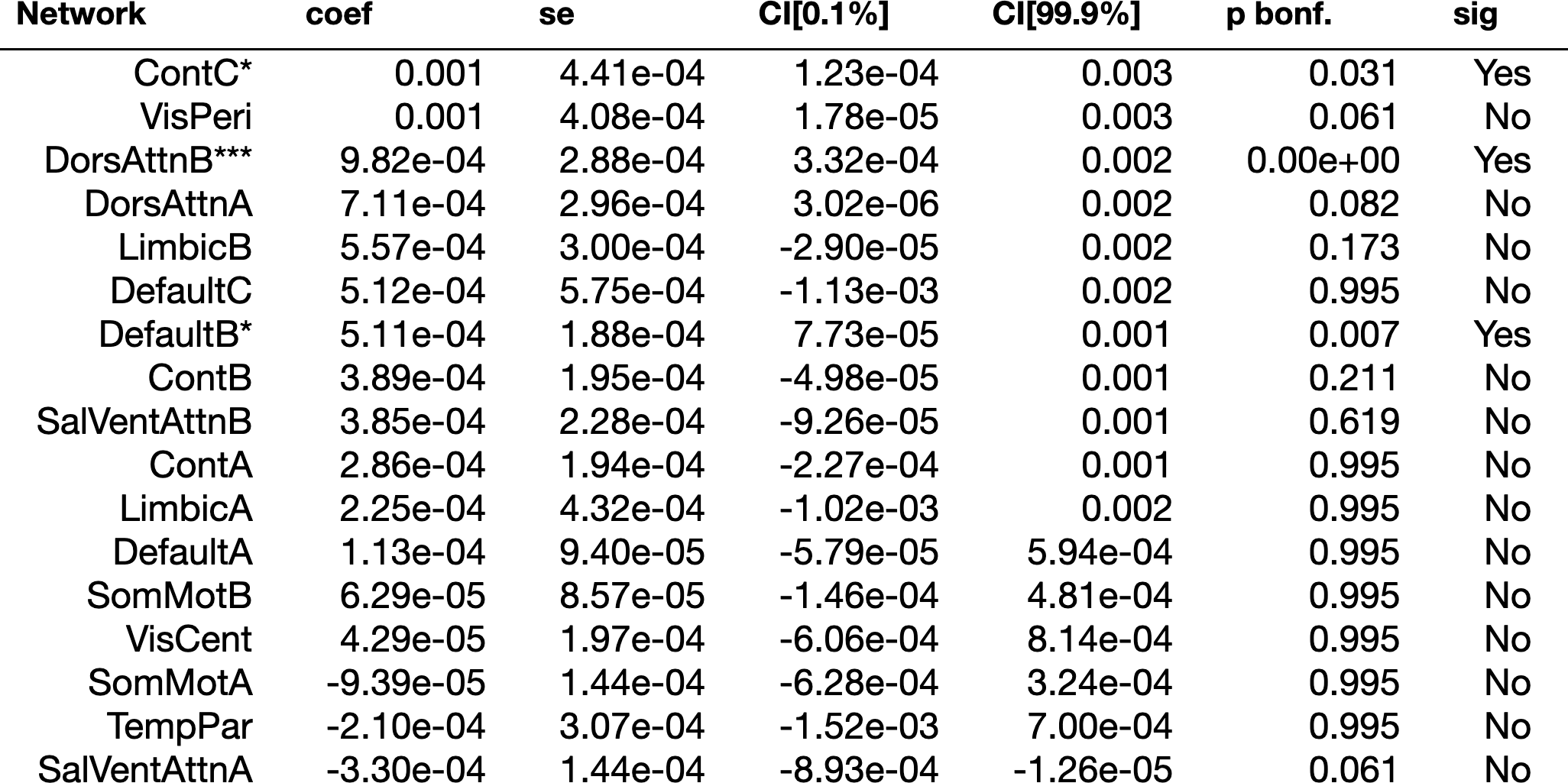
**

**Table S35. Statistics for the indirect mediation by redundancy with degree as a covariate on the relationship between age and average controllability for each of the 17 networks, with a global threshold of 0.010.** For 3 of 17 (ContC, DorsAttnB, DefaultB) networks there was a significant mediation by redundancy. Education was included as a covariate for all mediations. Significance was determined if the confidence intervals for each coefficient did not cross zero after setting the α = 0.05/17 to correct for multiple comparisons. *corrected p bonf. < 0.05, **p bonf*.*  < 0.001, ***p bonf. < 1e-05.

**
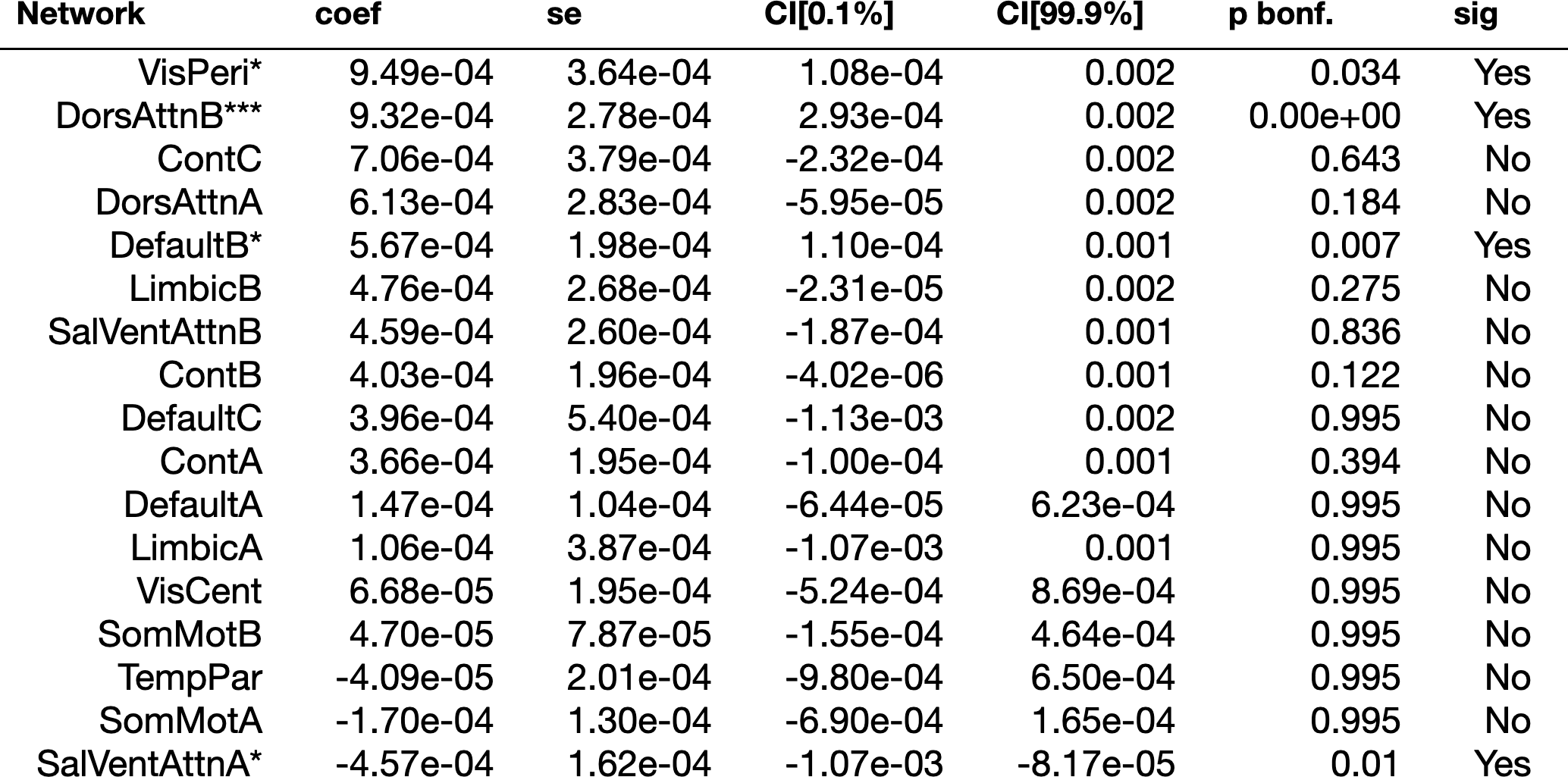
**

**Table S36. Statistics for the indirect mediation by redundancy with degree as a covariate on the relationship between age and average controllability for each of the 17 networks, with a global threshold of 0.015.** For 4 of 17 networks (VisPeri, DorsAttnB, DefaultB, SalVentAttnA) there was a significant mediation by redundancy. Education was included as a covariate for all mediations. Significance was determined if the confidence intervals for each coefficient did not cross zero after setting the α = 0.05/17 to correct for multiple comparisons. *corrected p bonf. < 0.05, **p bonf*.*  < 0.001, ***p bonf. < 1e-05.


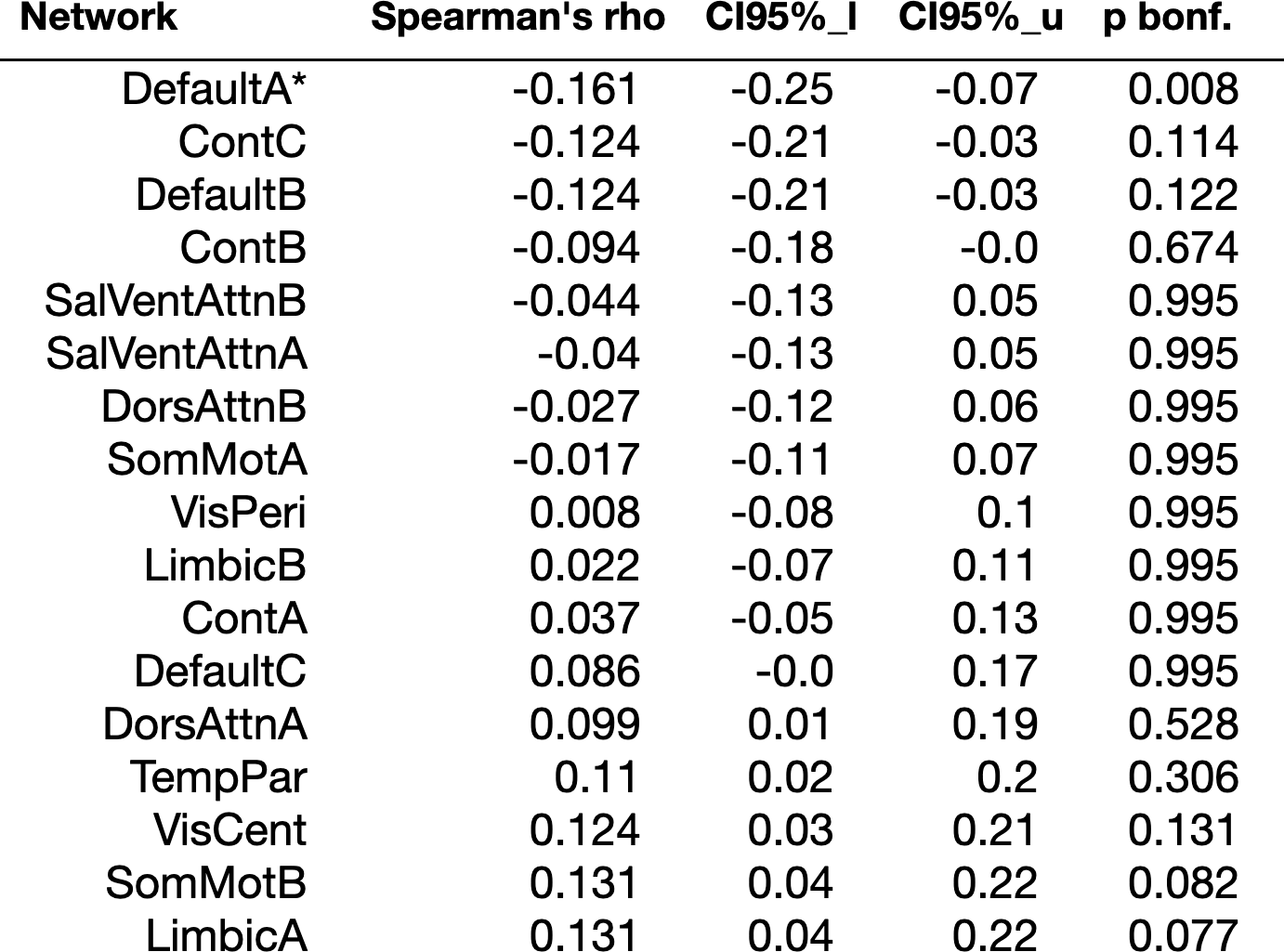


**Table S37. Mean network average controllability associated with average network redundancy, with global network thresholds of 0.001 the maximum streamline count per subject.** Mean network average controllability of only the default mode (DefaultA) networks showed a significant relationship with average network redundancy. Participant age and education was included as a covariate for all associations. Networks are sorted in ascending order by the calculated Spearman’s ρ’s. The Bonferroni method was used to correct for multiple comparisons. *corrected p bonf. < 0.05, **p bonf*.*  < 0.001, ***p bonf. < 1e-05.


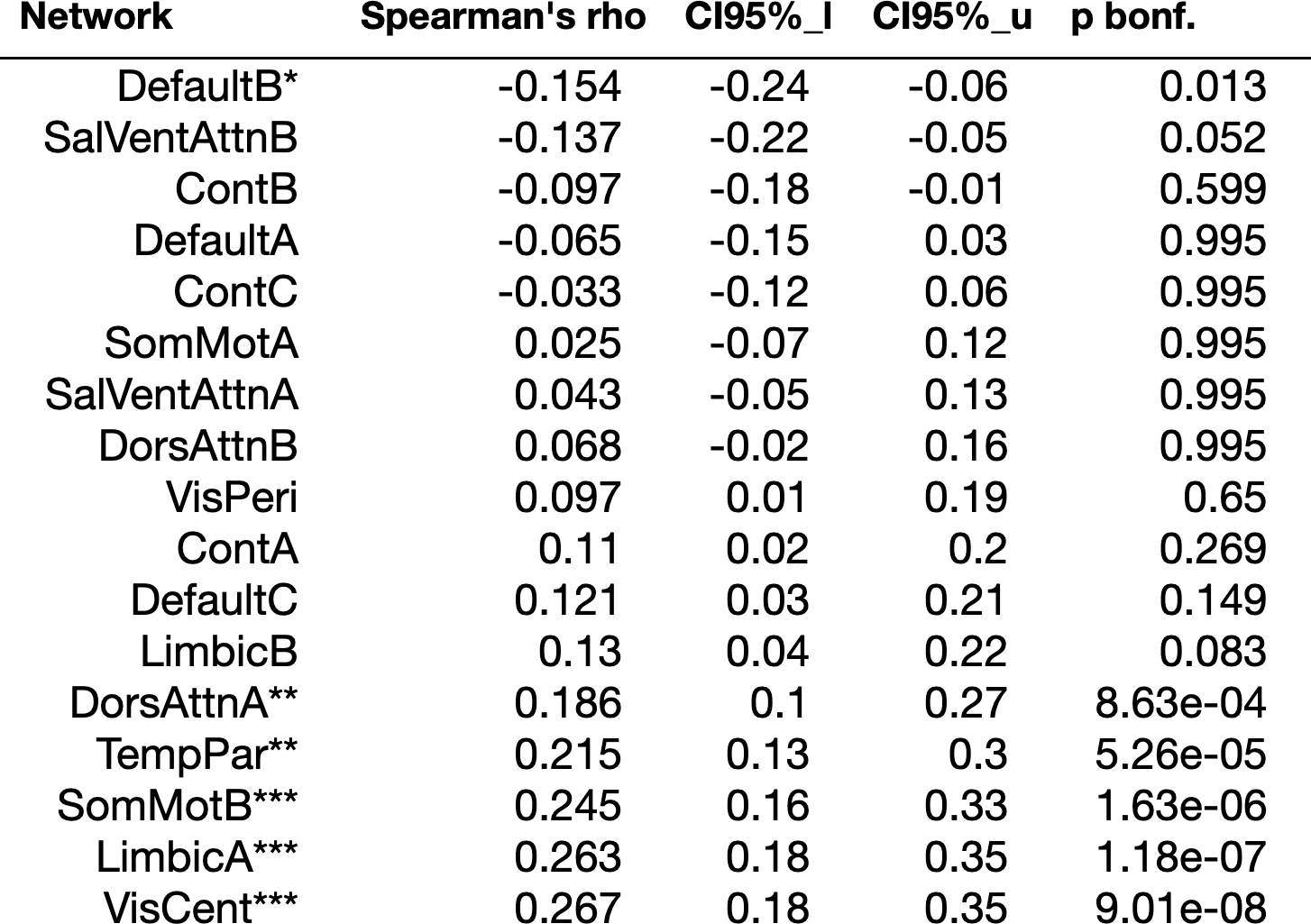


**Table S38. Mean network average controllability associated with average network redundancy, with global network thresholds of 0.005 the maximum streamline count per subject.** Mean network average controllability of 6 of the 17 networks showed significant relationships with average network redundancy. Participant age and education was included as a covariate for all associations. Networks are sorted in ascending order by the calculated Spearman’s ρ’s. The Bonferroni method was used to correct for multiple comparisons. *corrected p bonf. < 0.05, **p bonf*.*  < 0.001, ***p bonf. < 1e-05. Mean network degree was included as a covariate in all associations.


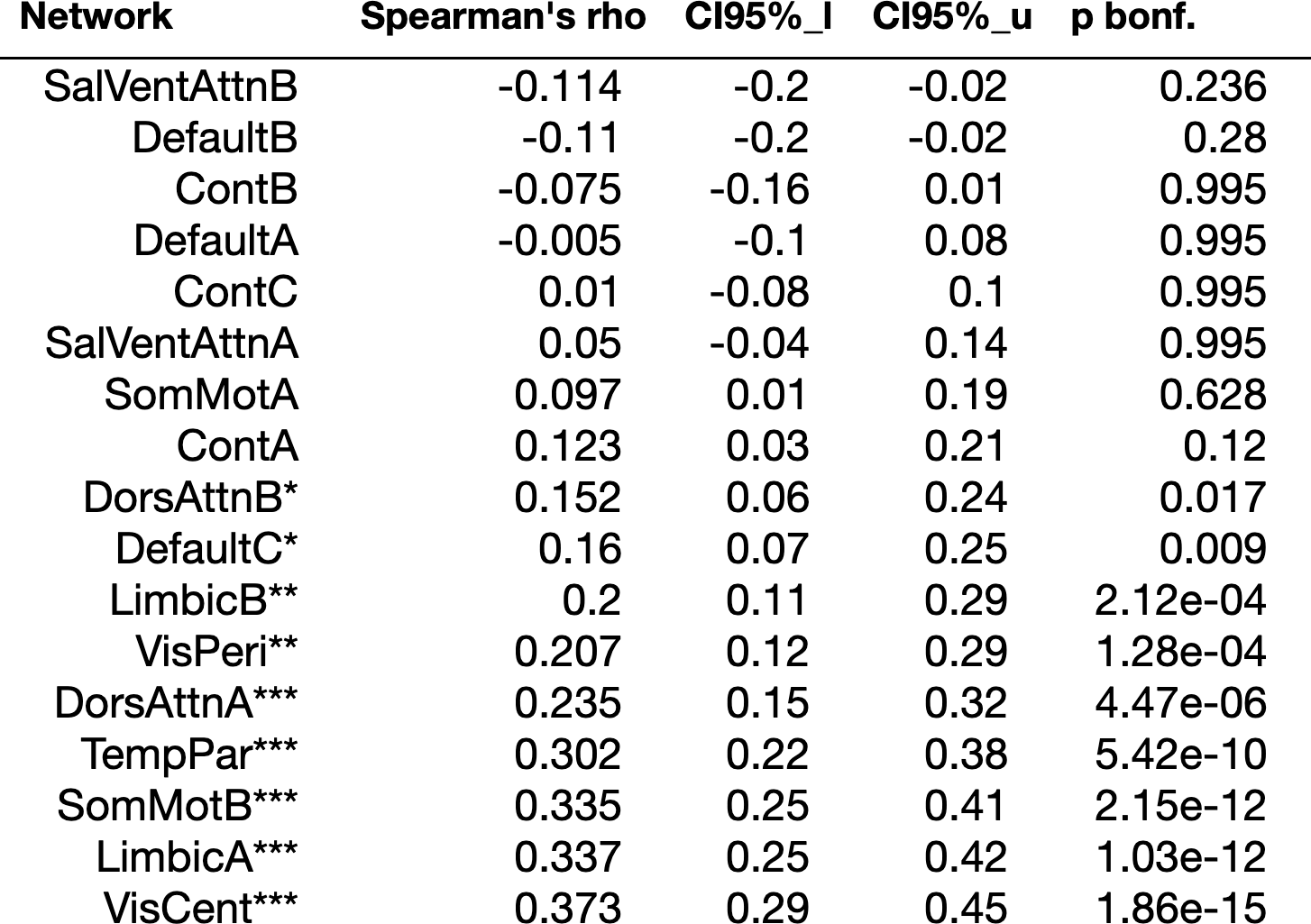


**Table S39. Mean network average controllability associated with average network redundancy, with global network thresholds of 0.010 the maximum streamline count per subject.** Mean network average controllability of 9 of the 17 networks showed significant relationships with average network redundancy. Participant age and education was included as a covariate for all associations. Networks are sorted in ascending order by the calculated Spearman’s ρ’s. The Bonferroni method was used to correct for multiple comparisons. *corrected p bonf. < 0.05, **p bonf*.*  < 0.001, ***p bonf. < 1e-05. Mean network degree was included as a covariate in all associations.

**
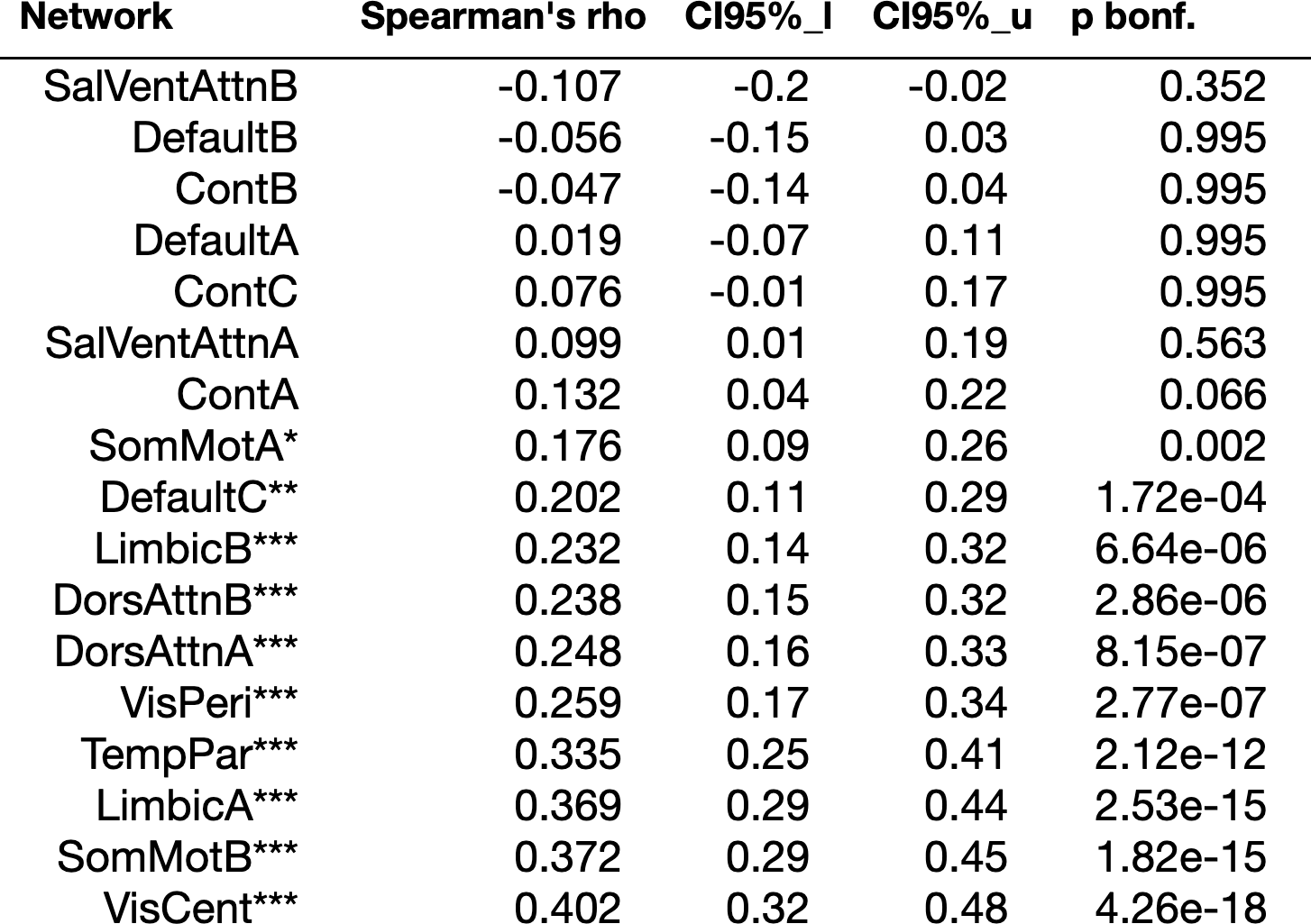
**

**Table S40. Mean network average controllability associated with average network redundancy, with global network thresholds of 0.015 the maximum streamline count per subject.** Mean network average controllability of 10 of the 17 networks showed significant relationships with average network redundancy. Participant age and education was included as a covariate for all associations. Networks are sorted in ascending order by the calculated Spearman’s ρ’s. The Bonferroni method was used to correct for multiple comparisons. *corrected p bonf. < 0.05, **p bonf*.*  < 0.001, ***p bonf. < 1e-05. Mean network degree was included as a covariate in all associations.


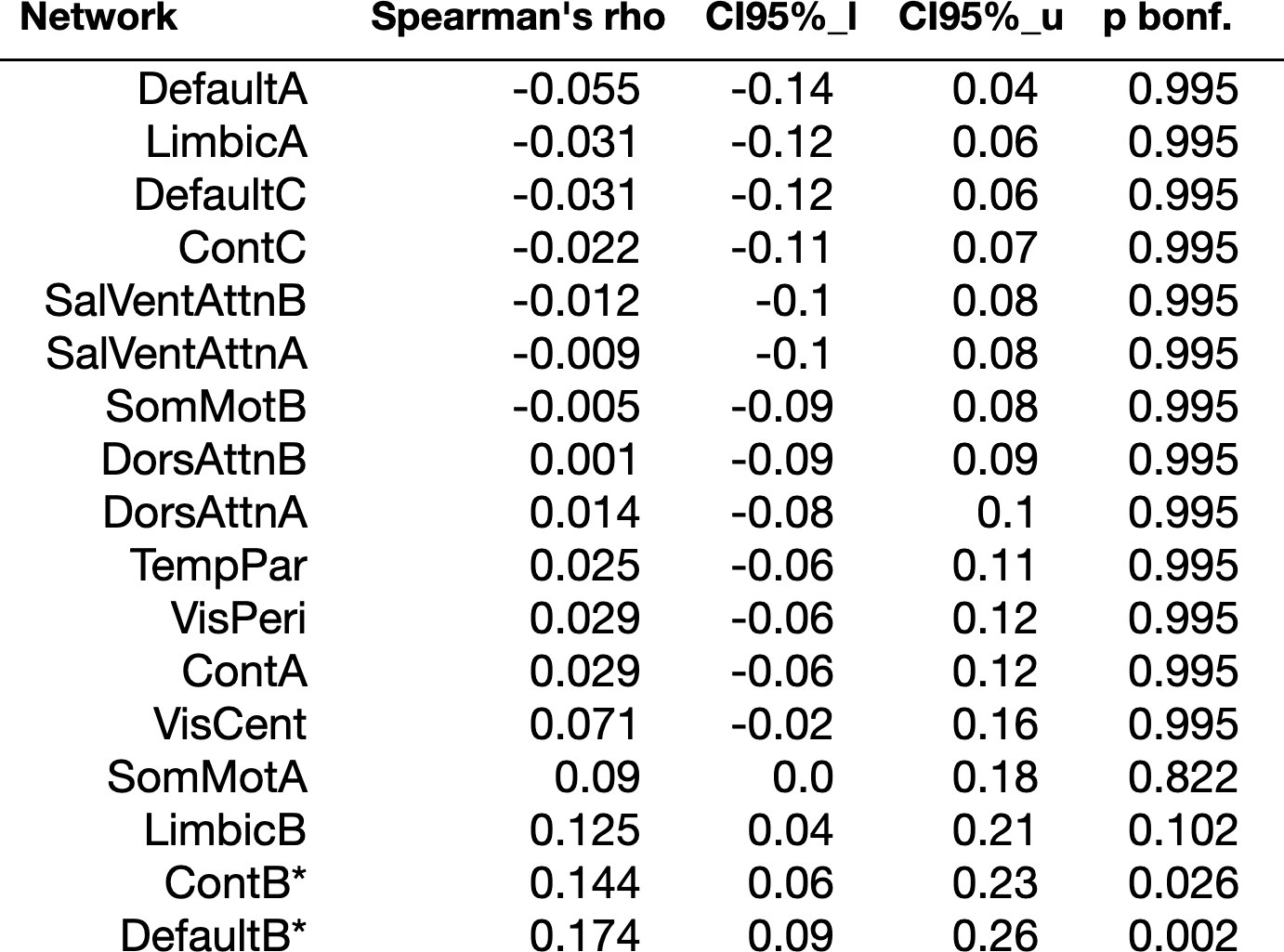


**Table S41. Mean network average controllability associated with processing speed, with global network thresholds of 0.001 the maximum streamline count per subject.** Mean network average controllability of the frontoparietal control (ContB) and default mode (DefaultB) networks showed a positive relationship with processing speed. Education was included as a covariate for all associations. Networks are sorted in ascending order by the calculated Spearman’s ρ’s. The Bonferroni method was used to correct for multiple comparisons. *corrected p bonf. < 0.05, **p bonf*.*  < 0.001, ***p bonf. < 1e-05.


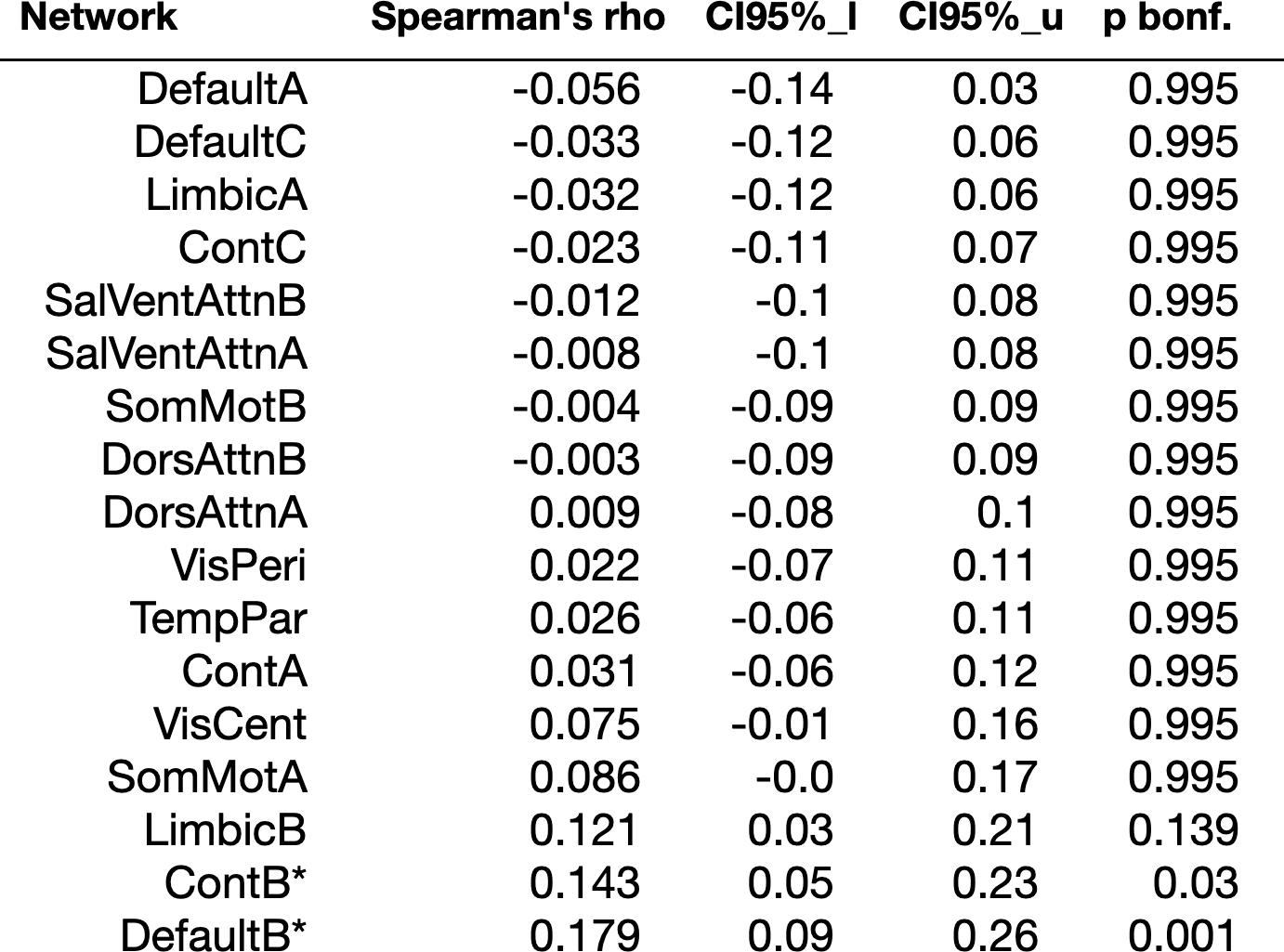


**Table S42. Mean network average controllability associated with processing speed, with global network thresholds of 0.050 the maximum streamline count per subject.** Mean network average controllability of the frontoparietal control (ContB) and default mode (DefaultB) networks showed a positive relationship with processing speed. Education was included as a covariate for all associations. Networks are sorted in ascending order by the calculated Spearman’s ρ’s. The Bonferroni method was used to correct for multiple comparisons. *corrected p bonf. < 0.05, **p bonf*.*  < 0.001, ***p bonf. < 1e-05.


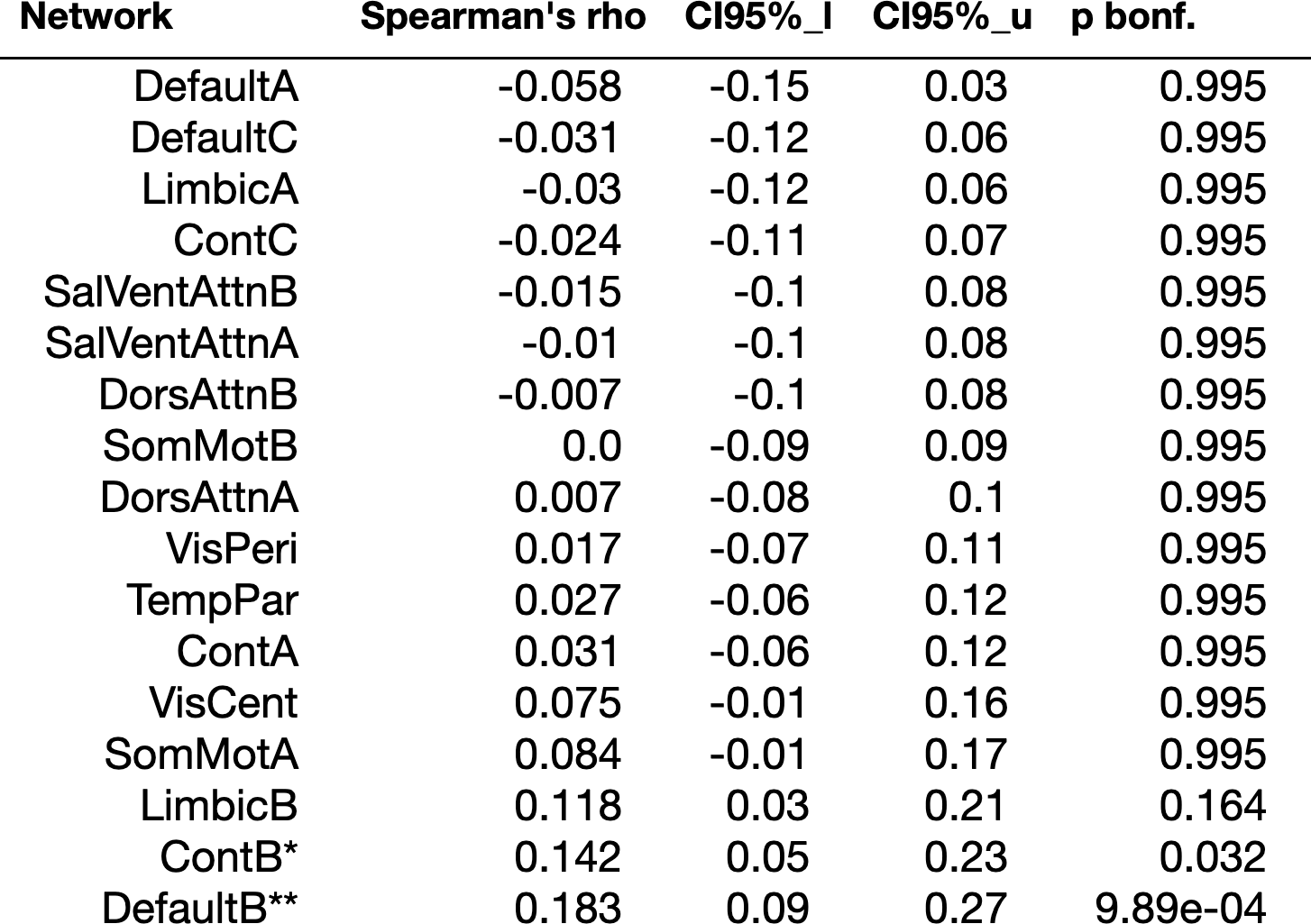


**Table S43. Mean network average controllability associated with processing speed, with global network thresholds of 0.010 the maximum streamline count per subject.** Mean network average controllability of the frontoparietal control (ContB) and default mode (DefaultB) networks showed a positive relationship with processing speed. Education was included as a covariate for all associations. Networks are sorted in ascending order by the calculated Spearman’s ρ’s. The Bonferroni method was used to correct for multiple comparisons. *corrected p bonf. < 0.05, **p bonf*.*  < 0.001, ***p bonf. < 1e-05.


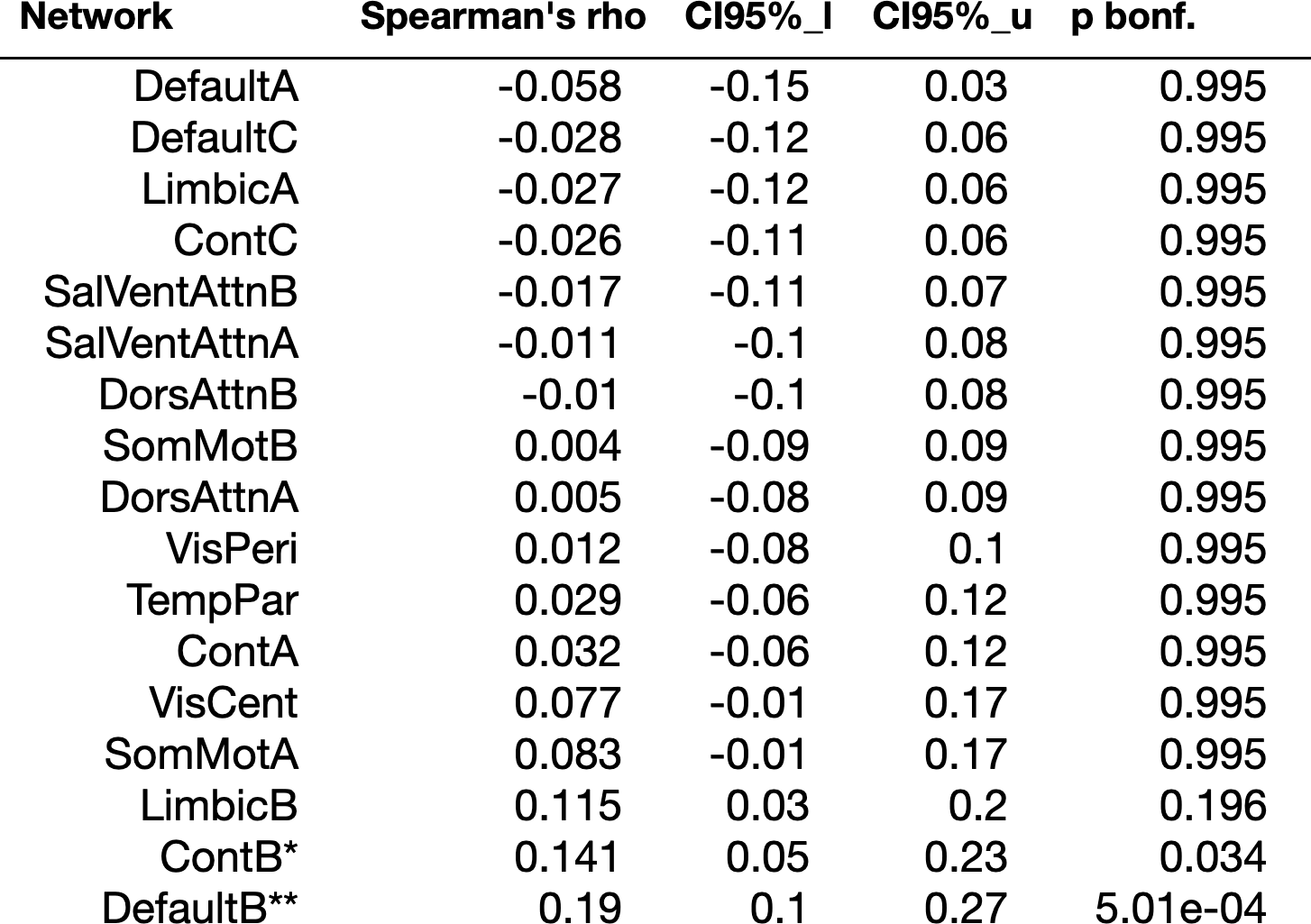


**Table S44. Mean network average controllability associated with processing speed, with global network thresholds of 0.015 the maximum streamline count per subject.** Mean network average controllability of the frontoparietal control (ContB) and default mode (DefaultB) networks showed a positive relationship with processing speed. Education was included as a covariate for all associations. Networks are sorted in ascending order by the calculated Spearman’s ρ’s. The Bonferroni method was used to correct for multiple comparisons. *corrected p bonf. < 0.05, **p bonf*.*  < 0.001, ***p bonf. < 1e-05.

**
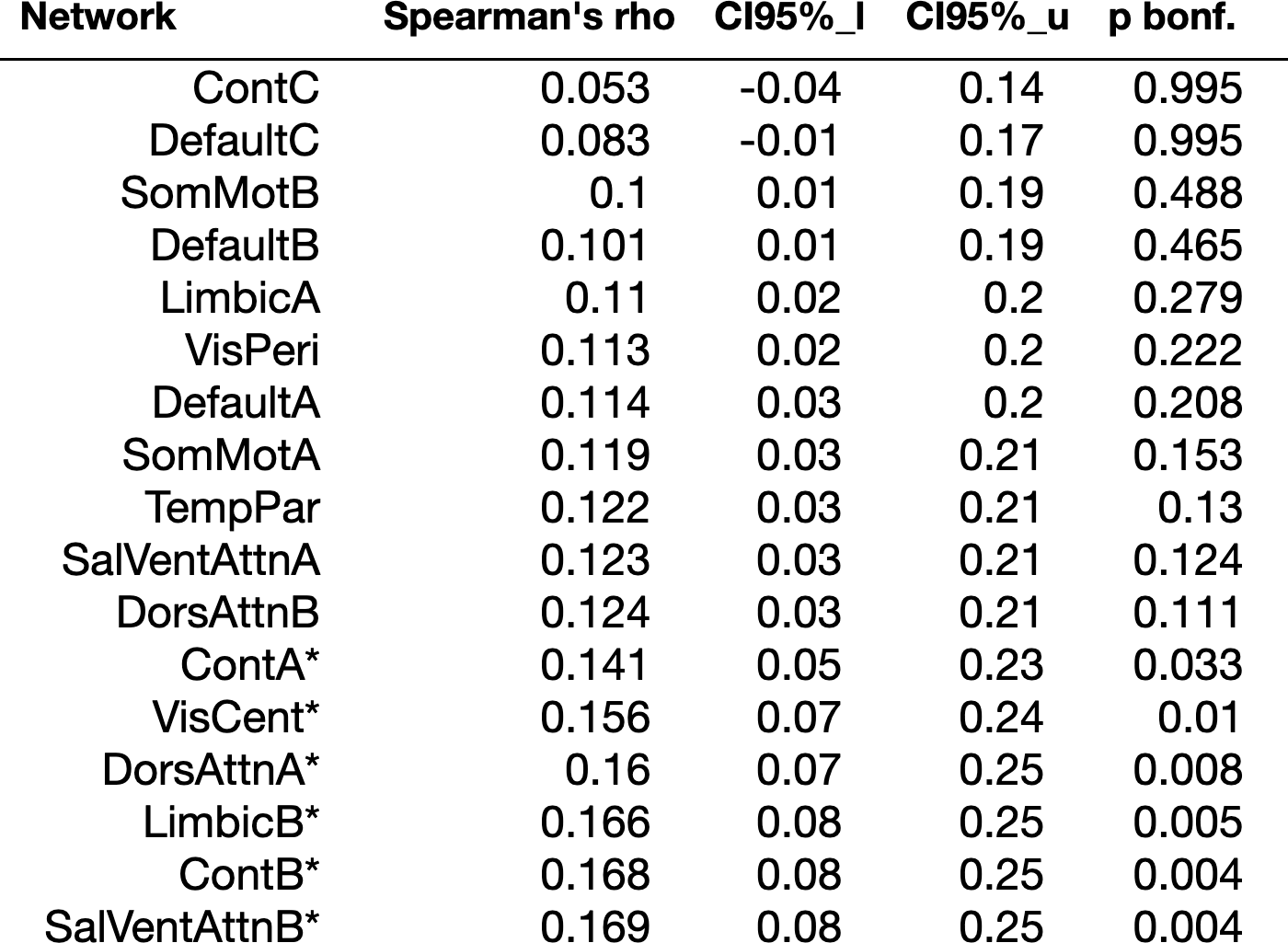
**

**Table S45. Average network redundancy associated with processing speed, with global network thresholds of 0.001 the maximum streamline count per subject.** Mean network average controllability of the salience/ventral attention (SalVentAttnB), limbic (LimbicB), frontoparietal control (ContB) and visual (VisCent), and dorsal attention (DorsAttnA) networks showed a positive relationship with processing speed. Education was included as a covariate for all associations. Networks are sorted in ascending order by the calculated Spearman’s ρ’s. The Bonferroni method was used to correct for multiple comparisons. *corrected p bonf. < 0.05, **p bonf*.*  < 0.001, ***p bonf. < 1e-05.


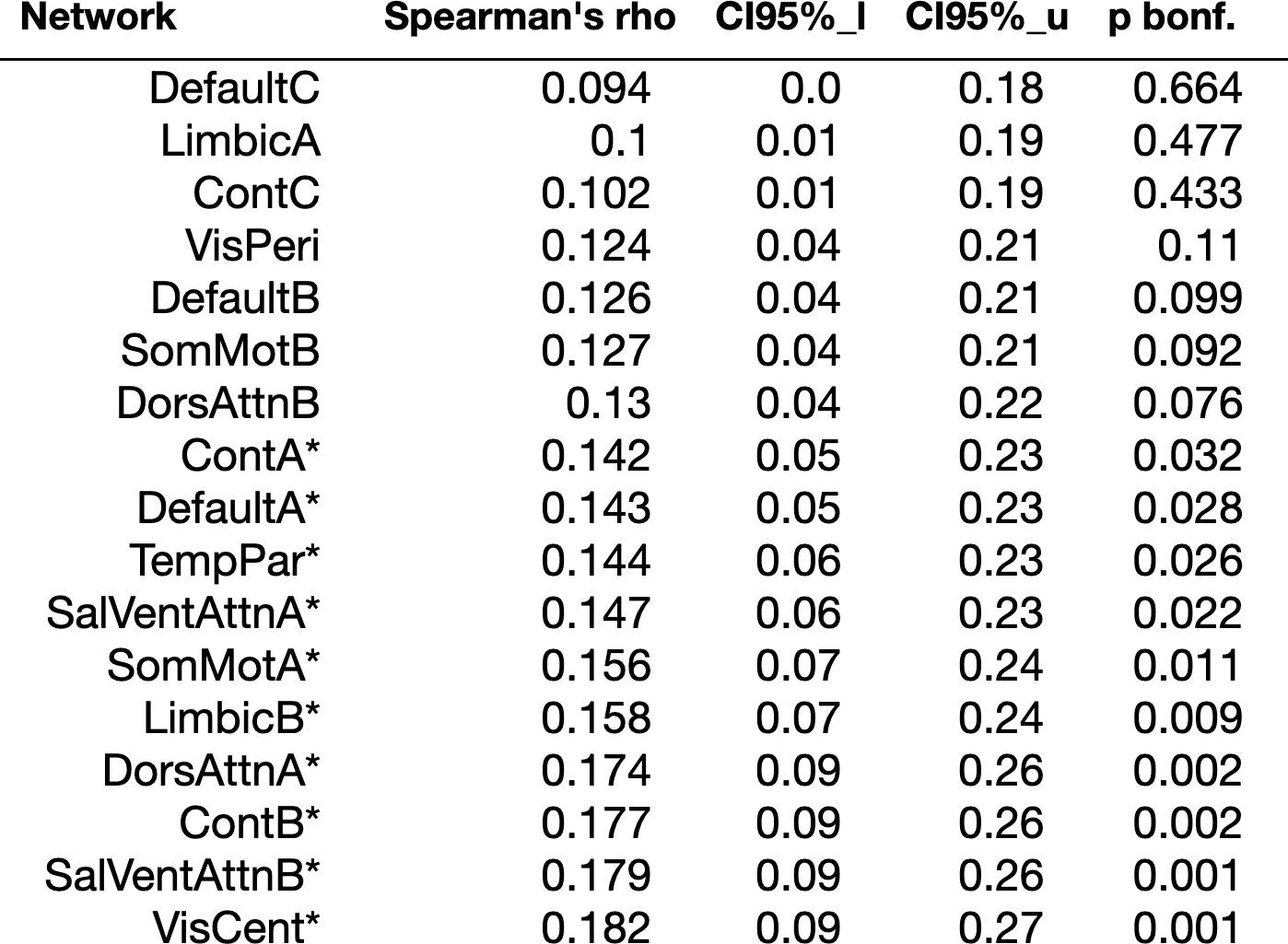


**Table S46. Average network redundancy associated with processing speed, with global network thresholds of 0.005 the maximum streamline count per subject.** For 10 of 17 networks there was a positive relationship with between redundancy and processing speed. Education was included as a covariate for all associations. Networks are sorted in ascending order by the calculated Spearman’s ρ’s. The Bonferroni method was used to correct for multiple comparisons. *corrected p bonf. < 0.05, **p bonf*.*  < 0.001, ***p bonf. < 1e-05.


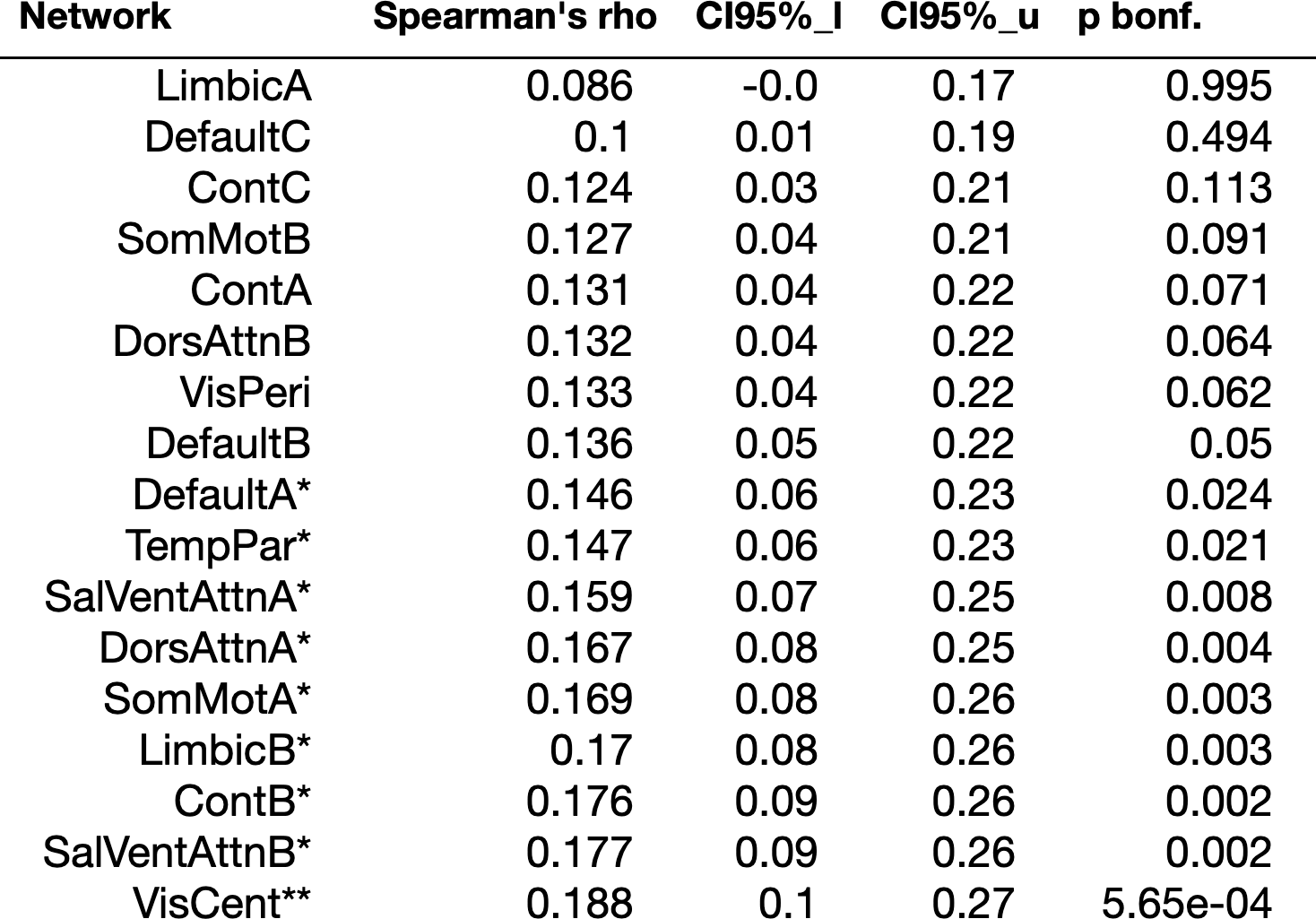


**Table S47. Average network redundancy associated with processing speed, with global network thresholds of 0.010 the maximum streamline count per subject.** Education was included as a covariate for all associations. For 9 of 17 networks there was a positive relationship with between redundancy and processing speed. Education was included as a covariate for all associations. Networks are sorted in ascending order by the calculated Spearman’s ρ’s. The Bonferroni method was used to correct for multiple comparisons. *corrected p bonf. < 0.05, **p bonf*.*  < 0.001, ***p bonf. < 1e-05.


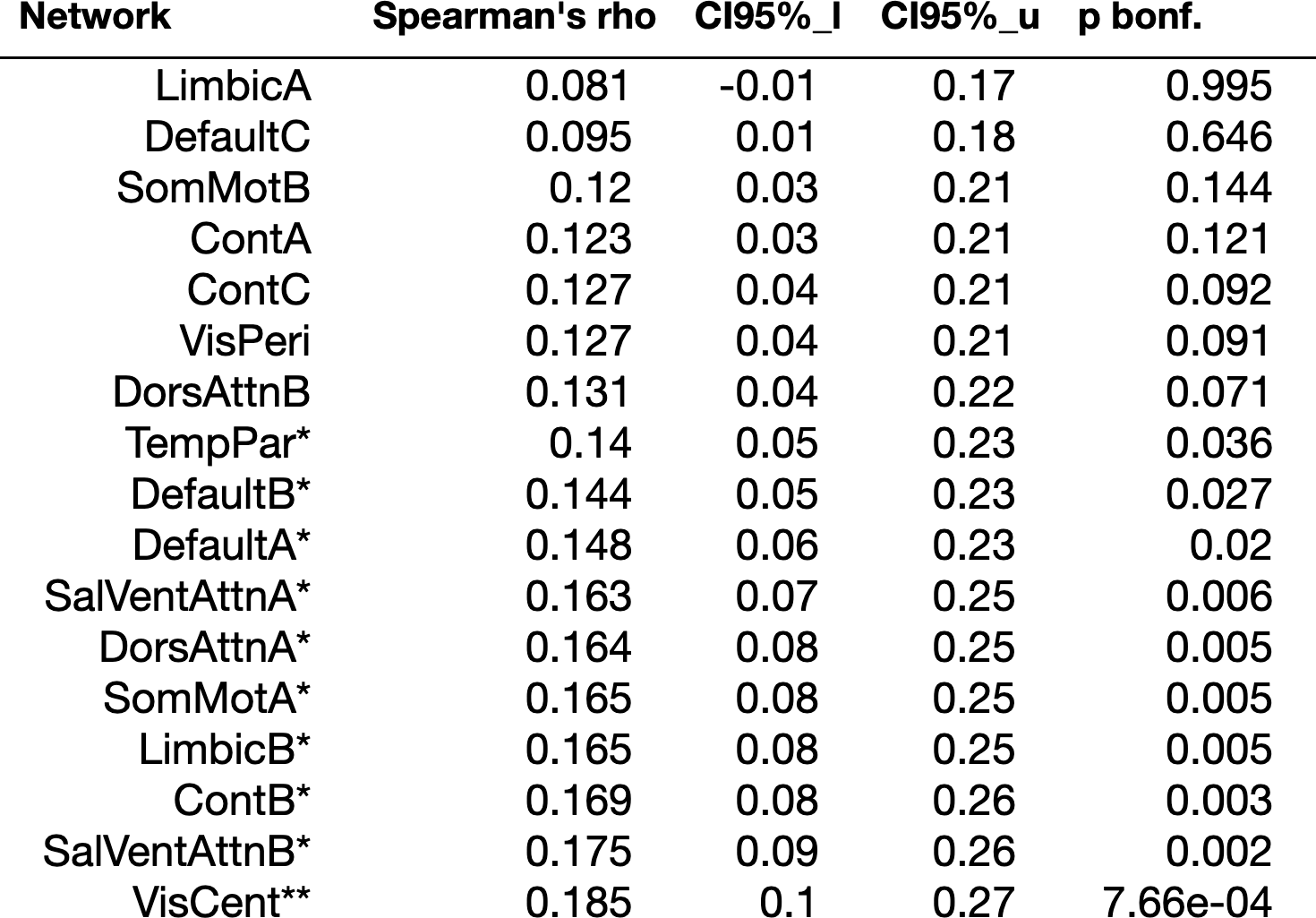


**Table S48. Average network redundancy associated with processing speed, with global network thresholds of 0.015 the maximum streamline count per subject.** For 10 of 17 networks there was a positive relationship with between redundancy and processing speed. Education was included as a covariate for all associations. Networks are sorted in ascending order by the calculated Spearman’s ρ’s. The Bonferroni method was used to correct for multiple comparisons. *corrected p bonf. < 0.05, **p bonf*.*  < 0.001, ***p bonf. < 1e-05.


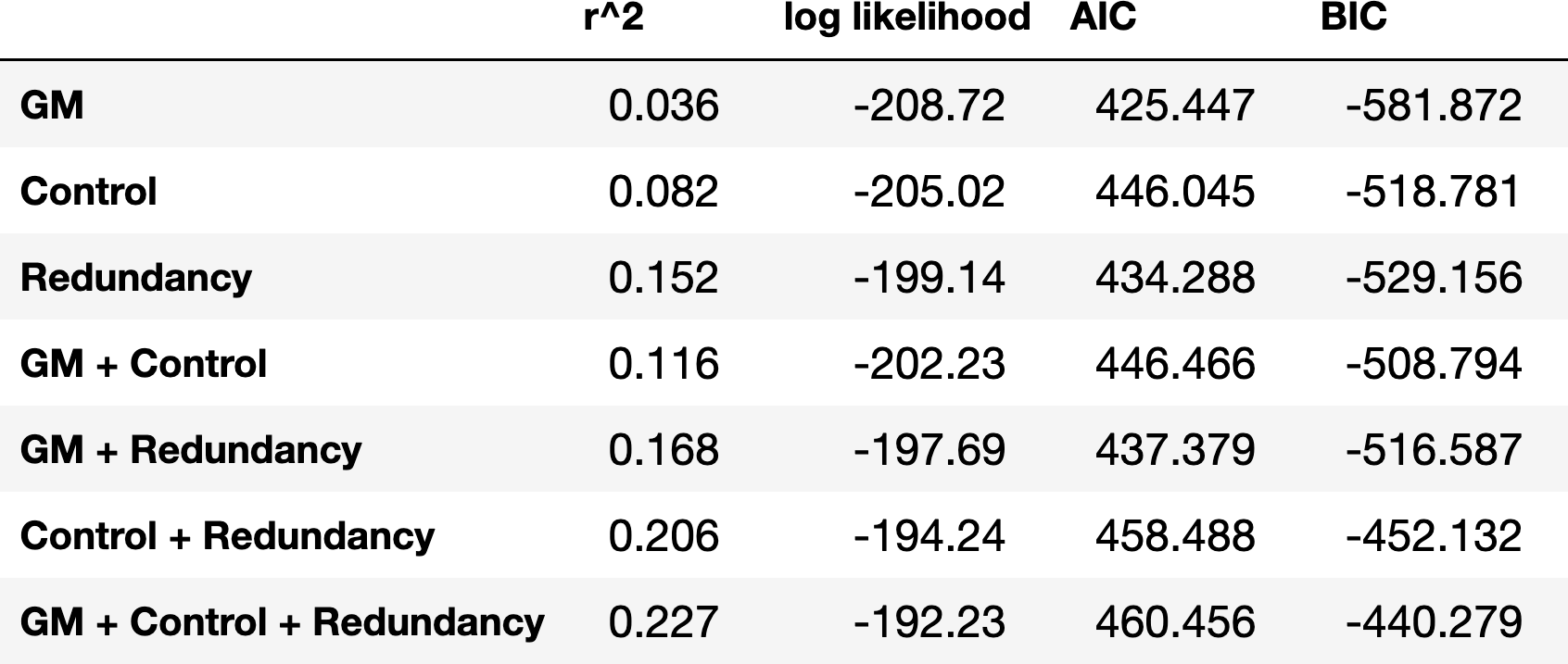


**Table S49**. **GM, mean network average controllability (Control), and average network redundancy (Redundancy), each aid in the prediction of processing speed in older adults, with a global network threshold of 0.005 of the maximum streamline count per participant.** The R^2^, log-likelihood, AIC, and BIC for each GLM trained to predict processing speed in older participants (ages > 66.92).


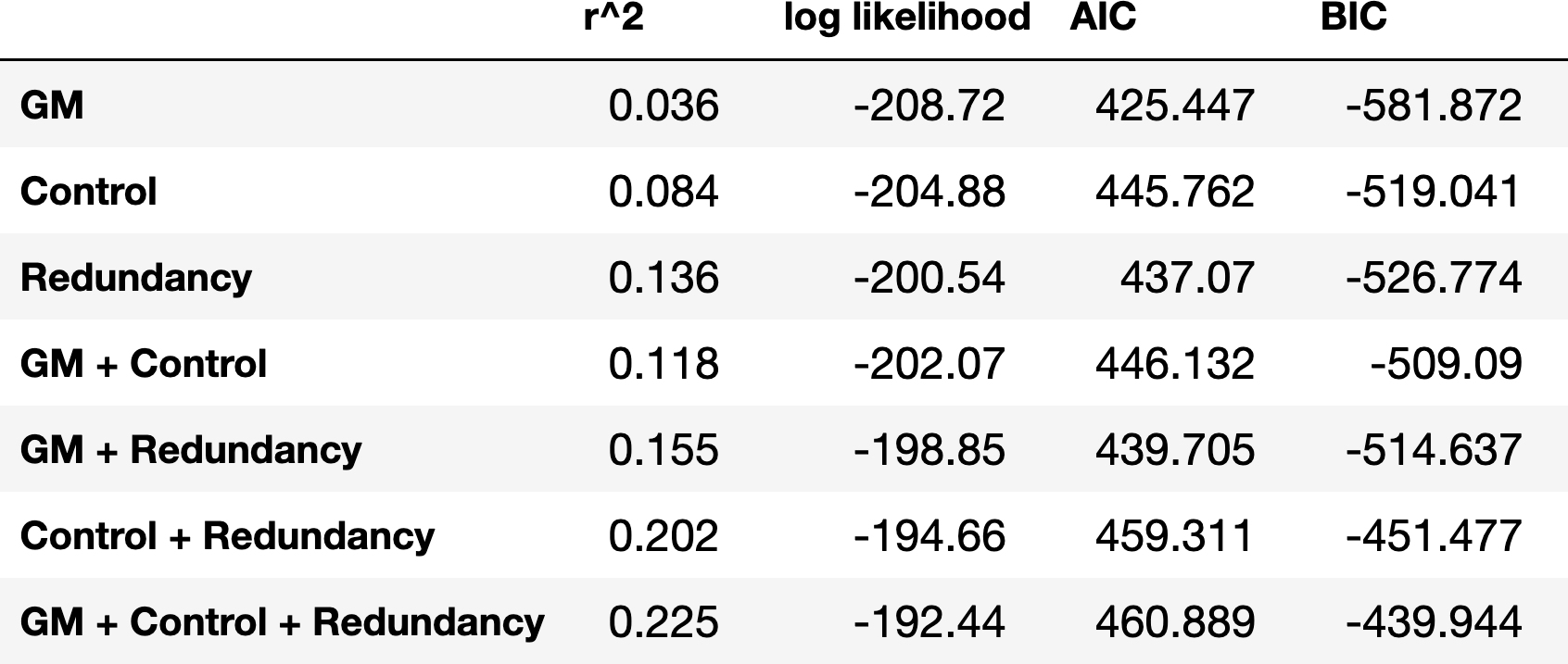


**Table S50**. **GM, mean network average controllability (Control), and average network redundancy (Redundancy), each aid in the prediction of processing speed in older adults, with a global network threshold of 0.010 of the maximum streamline count per participant.** The R^2^, log-likelihood, AIC, and BIC for each GLM trained to predict processing speed in older participants (ages > 66.92).


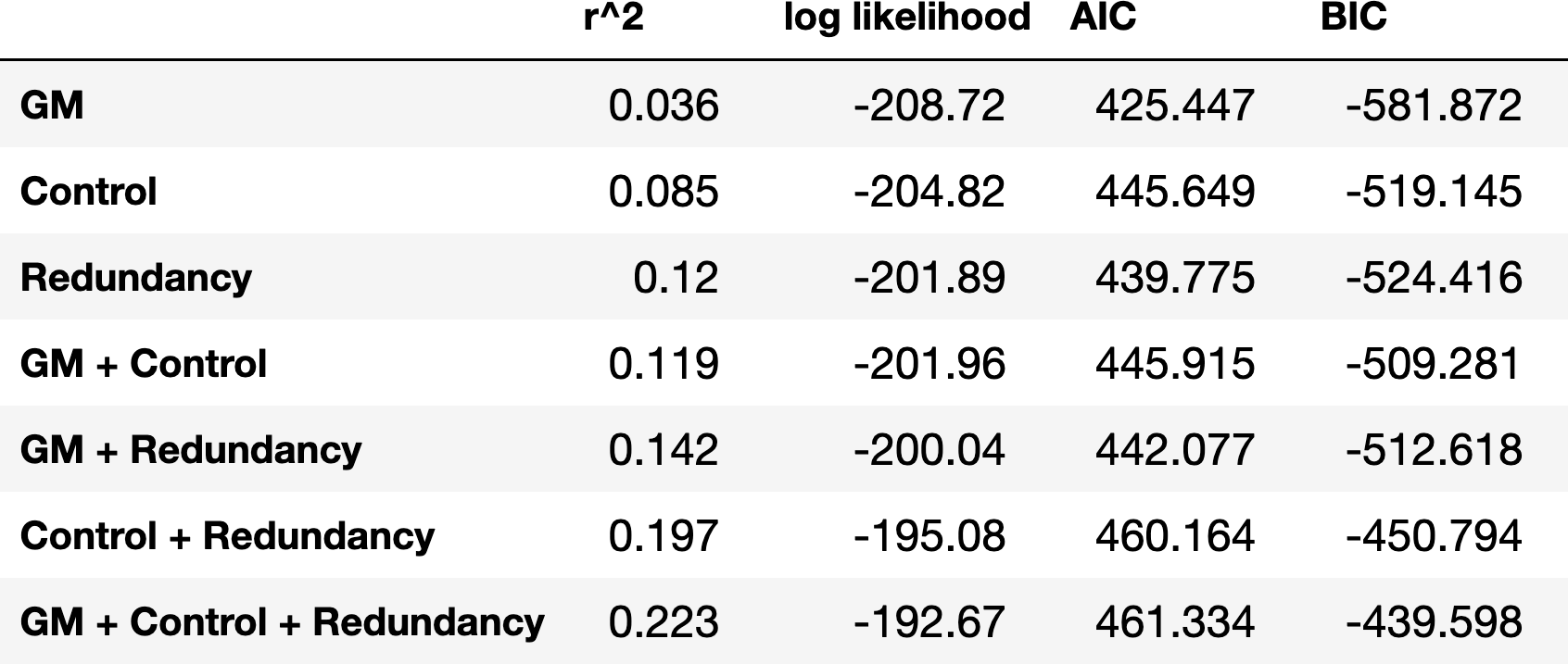


**Table S51**. **GM, mean network average controllability (Control), and average network redundancy (Redundancy), each aid in the prediction of processing speed in older adults, with a global network threshold of 0.015 of the maximum streamline count per participant.** The R^2^, log-likelihood, AIC, and BIC for each GLM trained to predict processing speed in older participants (ages > 66.92).
