# Supplementary material for: Age-related differences in network controllability are mitigated by redundancy in large-scale brain networks
Source: Commun Biol. 2024 Jun 7;7:701. doi: 10.1038/s42003-024-06392-2 (PMC11161655; doi:10.1038/s42003-024-06392-2)
Supplement: Supplementary file 2 — ReportingSummary [file 42003_2024_6392_MOESM2_ESM.pdf]

nature portfolio

Corresponding author(s): Eran Dayan

Last updated by author(s): May 15, 2024

Reporting Summary

Nature Portfolio wishes to improve the reproducibility of the work that we publish. This form provides structure for consistency and transparency in reporting. For further information on Nature Portfolio policies, see our Editorial Policies and the Editorial Policy Checklist.

Statistics

For all statistical analyses, confirm that the following items are present in the figure legend, table legend, main text, or Methods section.

n/a	Confirmed	
☐	☒	The exact sample size (*n*) for each experimental group/condition, given as a discrete number and unit of measurement
☐	☒	A statement on whether measurements were taken from distinct samples or whether the same sample was measured repeatedly
☐	☒	The statistical test(s) used AND whether they are one- or two-sided<br>*Only common tests should be described solely by name; describe more complex techniques in the Methods section.*
☐	☒	A description of all covariates tested
☐	☒	A description of any assumptions or corrections, such as tests of normality and adjustment for multiple comparisons
☐	☒	A full description of the statistical parameters including central tendency (e.g. means) or other basic estimates (e.g. regression coefficient) AND variation (e.g. standard deviation) or associated estimates of uncertainty (e.g. confidence intervals)
☐	☒	For null hypothesis testing, the test statistic (e.g. F, t, r) with confidence intervals, effect sizes, degrees of freedom and P value noted<br>*Give P values as exact values whenever suitable.*
☒	☐	For Bayesian analysis, information on the choice of priors and Markov chain Monte Carlo settings
☒	☐	For hierarchical and complex designs, identification of the appropriate level for tests and full reporting of outcomes
☐	☒	Estimates of effect sizes (e.g. Cohen's *d*, Pearson's *r*), indicating how they were calculated

Our web collection on statistics for biologists contains articles on many of the points above.

Software and code

Policy information about availability of computer code

Data collection	No software was used for data collection.
Data analysis	All code used in our analyses was obtained from publicly available repositories. Controllability metrics were computed using code from: https://github.com/BassettLab/nctpy. Degree and redundancy were computed using the "degree" and "all_simple_paths", respectively, from the python package NetworkX. Plotting was done using the python packages matplotlib and seaborn. Welch's ANOVAs, Spearman's correlations, Pearson's correlations, and mediation analysis, were performed using the python package Pingouin. Piece-wise linear regression to determine breakpoints in rates of change for grey matter volume was performed using the pwlf python package. Our GLMs were constructed using the python package Statsmodels. Additional stats derived from these models (R2, log-likelihood, AIC, BIC) were also computed using the Statsmodels package.<br><br>Support code for data preprocessing and statistical analyses can be found here: https://github.com/WilliamStanford/ControllabilityInAging/

For manuscripts utilizing custom algorithms or software that are central to the research but not yet described in published literature, software must be made available to editors and reviewers. We strongly encourage code deposition in a community repository (e.g. GitHub). See the Nature Portfolio guidelines for submitting code & software for further information.

Data

Policy information about availability of data

All manuscripts must include a data availability statement. This statement should provide the following information, where applicable:

- Accession codes, unique identifiers, or web links for publicly available datasets
- A description of any restrictions on data availability
- For clinical datasets or third party data, please ensure that the statement adheres to our policy

Data from all subjects used in this study is available through the Human Connectome Project: https://www.humanconnectome.org/study/hcp-lifespan-aging

Human research participants

Policy information about studies involving human research participants and Sex and Gender in Research.

Reporting on sex and gender	Sex and gender were not collected separately during initial data collection. Our results apply to both sexes as we did not consider each sex independently in our analyses. However, our dataset does contain more females than males (281 females, 199 males), which is reflective bias present in the HCP-Aging dataset.
Population characteristics	The HCP-Aging dataset is comprised of typically aging adults between the ages of 36-100+. Initial screening was performed to exclude participants with serious ongoing or previous health conditions such as major psychiatric disorders or neurological disorders. Further screening based on cognitive performance using the Telephone Interview for Cognitive Status, and the Montreal Cognitive Assessment Test for dementia.
Recruitment	Data was collected across three scanner sites (Washington University St. Louis, University of Minnesota, Massachusetts General Hospital and University of California, Los Angeles). Participants were recruited through a variety of sources, including advertisements, flyers, public seminars, and retirement homes.
Ethics oversight	All subjects provided written informed consent and all procedures were approved by the local Institutional Review Boards

Note that full information on the approval of the study protocol must also be provided in the manuscript.

Field-specific reporting

Please select the one below that is the best fit for your research. If you are not sure, read the appropriate sections before making your selection.

☒ Life sciences ☐ Behavioural & social sciences ☐ Ecological, evolutionary & environmental sciences

For a reference copy of the document with all sections, see nature.com/documents/nr-reporting-summary-flat.pdf

Life sciences study design

All studies must disclose on these points even when the disclosure is negative.

Sample size	We used all available participants within the HCP-Aging database that satisfied the criteria outlined in our methods (ages 40-90, n=480, 281 females, 199 males).
Data exclusions	Participants were excluded if they exhibited levels of cognitive performance that could be indicative of forms of dementia that were missed in the initial screenings done in the HCP-Aging study. Briefly, this involved excluding subjects between the ages of 65-90 if they performed worse than two standard deviations below the mean on measures of cognitive flexibility, vocabulary comprehension, and executive function.
Replication	Reproducibility of our findings were ensured via Bonferroni correction of multiple comparisons in all analyses, and bootstrapping of mediation analyses.
Randomization	Confidence intervals for the mediation analyses were computed with 10,000 bootstrap iterations.
Blinding	Data analysis was not performed blind. Blinding was unnecessary because the primary experiments performed were based on group membership, associations, and mediation analyses.

Reporting for specific materials, systems and methods

We require information from authors about some types of materials, experimental systems and methods used in many studies. Here, indicate whether each material, system or method listed is relevant to your study. If you are not sure if a list item applies to your research, read the appropriate section before selecting a response.

Materials & experimental systems

n/a	Involved in the study
☒	☐ Antibodies
☒	☐ Eukaryotic cell lines
☒	☐ Palaeontology and archaeology
☒	☐ Animals and other organisms
☒	☐ Clinical data
☒	☐ Dual use research of concern

Methods

n/a	Involved in the study
☒	☐ ChIP-seq
☒	☐ Flow cytometry
☐	☒ MRI-based neuroimaging

Magnetic resonance imaging

Experimental design

Design type	Diffusion MRI
Design specifications	No block-based design was used during scanning sessions.
Behavioral performance measures	Our primary analyses used a measure of processing speed assessed via the Pattern Comparison Processing Speed Test. We also utilized the scores for the Montreal Cognitive Assessment (MoCA), a measure of cognitive flexibility, assessed via the used the Dimensional Card Sort Test, executive control, assessed via the Flanker Inhibitory Control and Attention Test, and vocabulary comprehension assessed via the Picture Vocabulary Test, to define our exclusionary criteria that limited the possibility of including subjects with various forms of dementia.

Acquisition

Imaging type(s)	Structural, Diffusion
Field strength	3 Tesla
Sequence & imaging parameters	A multi-echo magnetization prepared rapid gradient echo (MPRAGE) sequence (voxel size: 0.8x0.8x0.8mm, TE = 1.8/3.6/5.4/7.2ms, TR = 2500ms, flip angle = 8 degrees)
Area of acquisition	Whole Brain

Diffusion MRI ☒ Used ☐ Not used

Parameters: Diffusion MRI (dMRI) images were generated from multi-shell diffusion with b-values of 1500 and 3000 s/mm2, with 93 and 92 sampling directions, a slice thickness of 1.5mm, and an in-plane resolution of 1.5mm

Preprocessing

Preprocessing software	Preprocessed dMRI data was reconstructed in DSI Studio (http://dsi-studio.labsolver.org). T1-weighted images were preprocessed with Freesurfer.
Normalization	T1-weighted images were non-linearly registered to the MNI-152 space. Diffusion MRI data linearly rotated to match the AC-PC line.
Normalization template	T1-weighted images were normalized to MNI-152 space before grey matter volume extraction.
Noise and artifact removal	T1-weighted images were bias corrected using FAST. dMRI data underwent TOPOP artifact susceptibility artifact detection using the Tiny FSL package (http://github.com/frankyeh/TinyFSL).
Volume censoring	None

Statistical modeling & inference

Model type and settings	Mass univariate, predictive, to evaluate cross-sectional relationships between age and network metrics.
Effect(s) tested	Statistical associations between network metrics and age and processing speed. Mediations analyses on the indirect effect of redundancy in the relationship between average network controllability and age. GLM's trained to predict cognitive performance using network metrics.

Specify type of analysis: ☐ Whole brain ☒ ROI-based ☐ Both

Anatomical location(s): The Local-Global Schaefer-Yeo 400 ROI cortical atlas was used.

Statistic type for inference (See Eklund et al. 2016)	Correlation coefficients, mediation analyses

Correction | We used Bonferroni correction for multiple comparisons in every analysis where appropriate.

Models & analysis

n/a	Involved in the study
☒	☐ Functional and/or effective connectivity
☐	☒ Graph analysis
☒	☐ Multivariate modeling or predictive analysis

Graph analysis | Weighted and binarized structural connectivity matrices.
